# A long noncoding RNA sensitizes genotoxic treatment by attenuating ATM activation and homologous recombination repair in cancers

**DOI:** 10.1371/journal.pbio.3000666

**Published:** 2020-03-23

**Authors:** Kunming Zhao, Xingwen Wang, Xuting Xue, Li Li, Ying Hu

**Affiliations:** 1 School of Life Science and Technology, Harbin Institute of Technology, Harbin, Heilongjiang Province, China; 2 The fourth affiliated hospital, Harbin Medical University, Harbin, Heilongjiang Province, China; 3 Shenzhen Graduate School of Harbin Institute of Technology, Shenzhen, China; The University of Texas at Austin, UNITED STATES

## Abstract

Ataxia-telangiectasia mutated (ATM) is an apical kinase of the DNA damage response following DNA double-strand breaks (DSBs); however, the mechanisms of ATM activation are not completely understood. Long noncoding RNAs (lncRNAs) are a class of regulatory molecules whose significant roles in DNA damage response have started to emerge. However, how lncRNA regulates ATM activity remains unknown. Here, we identify an inhibitor of ATM activation, lncRNA HITT (HIF-1α inhibitor at translation level). Mechanistically, HITT directly interacts with ATM at the HEAT repeat domain, blocking MRE11-RAD50-NBS1 complex–dependent ATM recruitment, leading to restrained homologous recombination repair and enhanced chemosensitization. Following DSBs, HITT is elevated mainly by the activation of Early Growth Response 1 (EGR1), resulting in retarded and restricted ATM activation. A reverse association between HITT and ATM activity was also detected in human colon cancer tissues. Furthermore, HITTs sensitize DNA damaging agent–induced cell death both in vitro and in vivo. These findings connect lncRNA directly to ATM activity regulation and reveal potential roles for HITT in sensitizing cancers to genotoxic treatment.

## Abstract 2

ATM is a crucial kinase in the cellular response to double-strand DNA breaks, but its mechanism of activation is not fully understood. This study identifies a novel lncRNA, HITT, that plays an essential role in inhibiting ATM activity and sensitizes cancer cells to DNA-damaging agents.

## Introduction

Cells are inevitably challenged by endogenous or exogenous sources of DNA damage [1]. To preserve genetic integrity, organisms have evolved elegant mechanisms to cope with various forms of DNA damage, collectively known as the DNA damage response (DDR) [2]. Deficiency in the DDR is associated with genomic instability, predisposition to cancer, or cell death in cases when damage is irreparable [3]. DNA damage also represents the backbone of cancer treatment. In this context, activation of DNA damage repair pathways promotes genotoxic resistance, which remains a major obstacle in successful cancer treatment [4,5]. Thus, unveiling the mechanisms underlying the DDR may not only inform our knowledge of tumorigenesis but also provide predictive markers for the patients’ responses to therapeutic DNA damage and offer new opportunities for the improvement of treatment efficiency.

Double-strand breaks (DSBs) are the most toxic DNA lesion that can arise following ionizing radiation (IR) or DNA-based chemotherapy [4]. Cells utilize two prominent pathways to repair DSBs: homologous recombination (HR) and nonhomologous end joining (NHEJ) [6]. Ataxia-telangiectasia (A-T) mutated (ATM) is an apical kinase of phosphorylation cascades in the HR pathway, at the heart of the cellular response to DSBs [7]. One of the most striking features of patients with A-T, a disorder caused by somatic ATM mutation, is their sensitivity to IR and DNA-damaging agents that induce DSBs [8]. Conversely, activation of ATM is linked to the survival of cancer cells following therapy [9,10]. ATM is an attractive target for cancer treatment. Several highly selective small molecule inhibitors of ATM have entered clinical trials in patients with advanced cancers [11,12].

Given its physiological and pathological significance, the regulation of ATM activity remains a major focus of the field. The MRE11-RAD50-NBS1 (MRN) complex is vital for the ATM-dependent DDR [13,14]. MRN is the first complex to be recruited to DSB sites, where it provides a platform for ATM recruitment and facilitates the autophosphorylation of ATM at serine (S) 1981, leading to optimal ATM activation that subsequently triggers the phosphorylation of a variety of ATM effectors essential for the DDR, such as checkpoint kinase 2 (Chk2) and Breast Cancer gene 1 (BRCA1) [15]. Interestingly, mutations in MRN component genes Meiotic Recombination 11 (Mre11) or Nijmegen Breakage Syndrome 1 (NBS1), which are linked to A-T-like disorder and Nijmegen breakage syndrome, respectively, are accompanied by defective ATM activity with symptoms resembling those of A-T patients [16,17], suggesting that MRN is required for the activation of ATM in vivo.

MRN is clearly involved in ATM activation at DSBs; however, how it activates this is still not fully understood. In addition, it has recently become apparent that multiple layers of regulation exist to ensure that the ATM signal is accurate and restricted to appropriate cellular contexts. For example, posttranslational modifications of MRN components, such as NBS1 ubiquitination [18] or MRE11 UFMylation [19], promote MRN-mediated ATM recruitment to DSB sites and thus play important roles in initiating or amplifying the ATM signal. It has also been noted that the rise and fall of ATM activity are equally important for the proper execution of DSB repair [6,20,21]. However, compared with the mechanisms of ATM activation, much less is known about the mechanisms of ATM inhibition.

To complicate the matter, evidence is steadily accumulating that long noncoding RNAs (lncRNAs), a class of transcribed RNA molecules greater than 200 nucleotides (nt) in length with extremely limited protein-coding capabilities, play essential roles in a wide range of physiological and pathological processes [22,23]. Interestingly, lncRNA integrates contextual and environmental cues not only during development but also under multiple stresses [24]. A variety of lncRNAs are DNA damage responsive and able to modulate the DNA repair program in turn, which highlights the essential roles of lncRNAs in the DDR. For example, lncRNAs DINO (Damage Induced Noncoding) [25], PANDA (P21 associated ncRNA DNA damage activated) [26], and linc-p21 [27] are induced upon DNA damage and are engaged in a feedback loop to regulate apoptosis or DNA repair by modulating p53 protein stability or p53’s transcriptional activity. However, despite being a master regulator of the DDR, whether and how ATM activation is directly regulated by lncRNAs remains to be determined.

We recently identified an lncRNA, linc00637, which is a 2,056-nt intergenic lncRNA containing three exons mapped at 14q32, a chromosome region that has been associated with the early onset and metastatic recurrence of colon cancer and many other types of cancers [28–30]. We found that linc00637 is down-regulated in multiple types of cancers. Further functional studies reveal that it is a hypoxia-responsive lncRNA, whose expression is reduced in the face of hypoxic stress and which plays essential roles in inhibiting hypoxia-inducible factor 1α (HIF-1α), mainly by repressing its translation; thus, we named it HITT (HIF-1α inhibitor at translation level) [31]. Besides hypoxia, cancer cells are also constantly insulted by DNA damage, particularly when receiving conventional genotoxic treatments. Thus, we wondered whether HITT is a stress responsive gene that plays roles in regulating the DDR. To this end, we explored the expression, function, and mechanisms of HITT upon DSBs. Interestingly, we identify HITT as a physiological brake of ATM activation, which is induced and maintained at high levels after DSBs and contributes to the promoted chemosensitization. Mechanistically, HITT is physiologically associated with ATM protein, thereby blocking MRN-mediated ATM recruitment. Thus, HITT represents the first RNA molecule that is directly involved in ATM regulation and may be a potential treatment for anticancer chemosensitization.

## Results

### DSB-induced HITT expression represses HR repair

**Fig 1:**
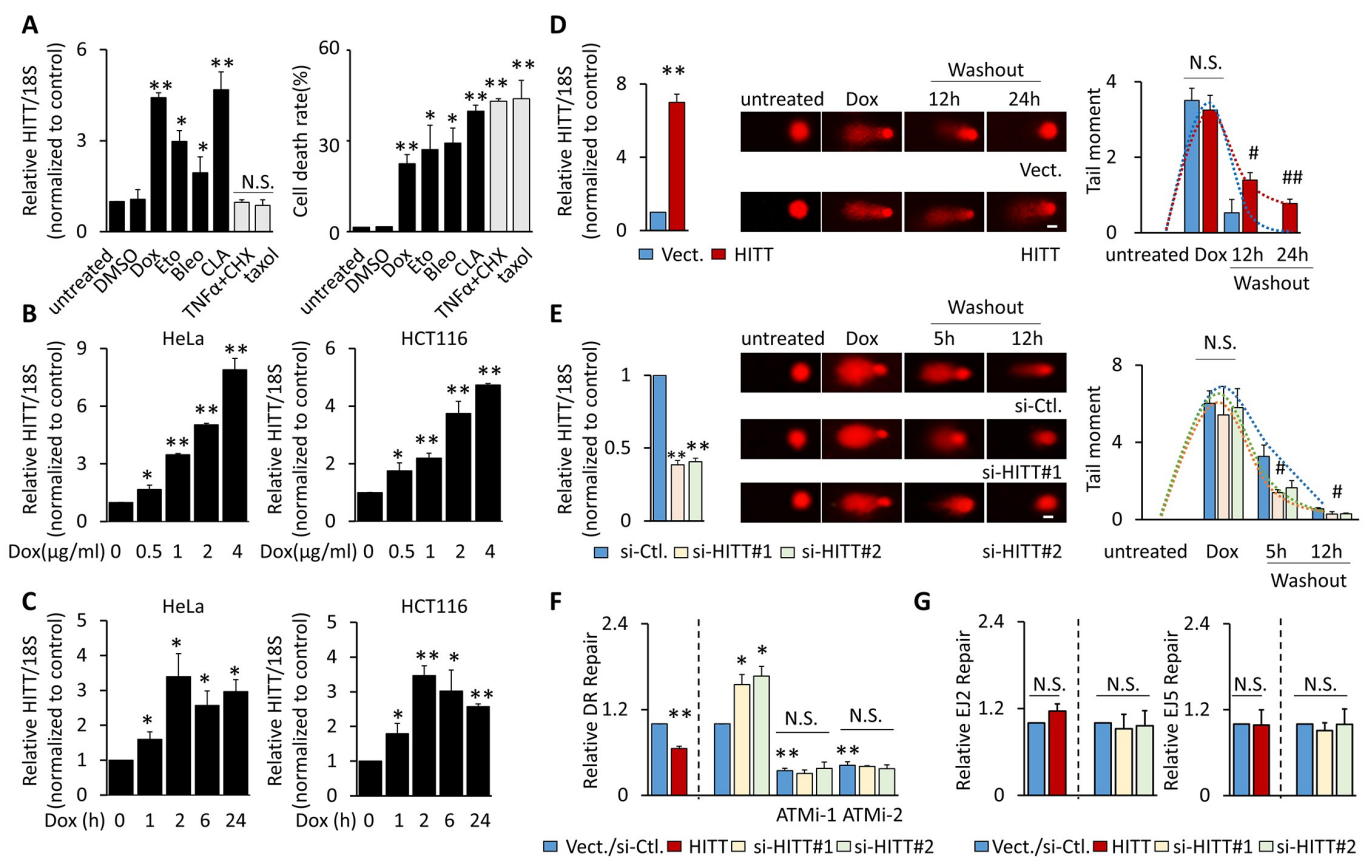
DSB-induced HITT expression attenuates HR repair. **(A)** Expression of HITT (left) and cell death rates (right) were determined by real-time RT-PCR and trypan blue exclusion assay, respectively, in HCT116 cells with the indicated treatments for 24 h: Dox (1 μg/ml), Eto (10 μM), Bleo (1 μg/ml), CLA (10 nM), TNF-α (10 ng/ml) + CHX (10 μM), taxol (10 nM). **(B, C)** Expression of HITT was determined by real-time RT-PCR after treating HeLa and HCT116 cells with different concentrations of Dox for 24 h (B), with 1 μg/ml Dox at different time periods (C). **(D, E)** Representative real-time RT-PCR bar graph showing the efficiency of HITT overexpression (D) or KD (E) in HCT116 cells (left). DNA damage was monitored by comet assay after DMSO or Dox (1 μg/ml) treatment, or at different periods of time following Dox washout (middle). Tail moment per cell is analyzed as described in the Materials and methods and presented in the bar graph (right), scale bar, 10 μm. **(F, G)** HR or NHEJ efficiencies of ISce-I–induced DSBs in U2OS cells containing DR-GFP (HR, F) or EJ2- or EJ5-GFP reporter (NHEJ, G) were determined by measuring GFP-positive cells by flow cytometry (FACS) after overexpression or KD of HITT in the presence or absence of 10 μM ATMi-1 or ATMi-2. Data are derived from three independent experiments and presented as mean ± SEM in the bar graphs. Values of controls were normalized to 1. *P < 0.05; **P < 0.01; relative to the untreated control (“Ctl.”) (A, B, C, F, and G); #*P* < 0.05; ##*P* < 0.01; relative to vector (“vect.”) or si-Ctl. with the indicated treatment (D and E). See also S1 Fig. The raw data see Fig 1A-G in S1 Data. ATMi-1, KU-60019; ATMi-2, KU-55933; Bleo, bleomycin; CHX, cycloheximide; CLA, calicheamicin; Dox, doxorubicin; DR, Direct Repeat; DSB, double-strand break; Eto, etoposide; FACS, fluorescence-activated cell sorting; GFP, green fluorescent protein; HITT, HIF-1α inhibitor at translation level; HR, homologous recombination; KD, knockdown; NHEJ, nonhomologous end joining; N.S., not significant; RT-PCR, reverse transcription PCR; si-, small interfering; TNF-α, tumor necrosis factor-α.

To understand whether HITT is a DNA damage-responsive lncRNA, expression levels of HITT were measured after exposing HCT116 cells to a panel of cytotoxic agents. HITT was significantly increased by drugs that have been reported to induce DSBs, such as doxorubicin (Dox), etoposide (Eto), the radiomimetic compound bleomycin (Bleo), and calicheamicin (CLA) [32–34], but not DSB-independent pro-death treatments, such as tumor necrosis factor-α (TNF-α)/cycloheximide (CHX) and taxol, although similar death rates were induced (Fig 1A). In addition, the induction of HITT was dose-dependent with an approximately 5- to 7-fold increase at 4 μg/ml Dox in both HeLa and HCT116 cells (Fig 1B). Time-course analysis revealed that HITT was increased with prolonged Dox treatment and reached a plateau after 2 h (Fig 1C). Similar time- or dose-dependent HITT expression patterns were detected after CLA, Eto, and Bleo exposure (S1A and S1B Fig). Furthermore, DSB-induced HITT expression occurred in a panel of cancer cells with different tissue origins, regardless of p53 status (S1C and S1D Fig). Therefore, DSB-induced HITT up-regulation is likely a common phenomenon that is independent of p53.

This inspired us to investigate whether HITT plays roles in regulating DSB repair progress. In a DNA comet assay, significant increases in comet tail scores were detected after Dox treatment, and this damage was rapidly repaired in a time-dependent manner after washing out of Dox (Figs 1D, 1E and S1E). Interestingly, stable HITT transfectants markedly attenuated the DDR process (Figs 1D and S1E). Two independent small interfering RNAs (siRNAs) specifically targeting HITT reduced HITT expression by approximately 50%. Accordingly, DNA repair progress was significantly accelerated (Figs 1E and S1E).

HR and NHEJ are two prominent pathways in regulating DSB repair. Thus, the efficiencies of HR and NHEJ were further compared after HITT overexpression or knockdown (KD) using cell lines containing the indicated integrated green fluorescent protein (GFP) reporters upon I-SceI-induced breaks [35]. RAD51 [36] and X-Ray Repair Cross Complementing 4 (XRCC4) [37] are key regulators of HR and NHEJ pathways, respectively. siRNA-mediated RAD51 and XRCC4 KD, respectively, reduced HR and NHEJ as expected (S1F Fig). Under such conditions, we found that HITT overexpression markedly inhibited HR, as indicated by Direct Repeat (DR)-GFP, but not NHEJ, as indicated by EJ2- and EJ5-GFP (Fig 1F and 1G). HITT KD by two independent siRNAs constantly and significantly increased HR but not NHEJ (Fig 1F and 1G). These data demonstrate that HITT is up-regulated upon DSBs independently of p53 and plays important roles in restraining HR repair.

### HITT reduces HR repair by repressing ATM activation

**Fig 2:**
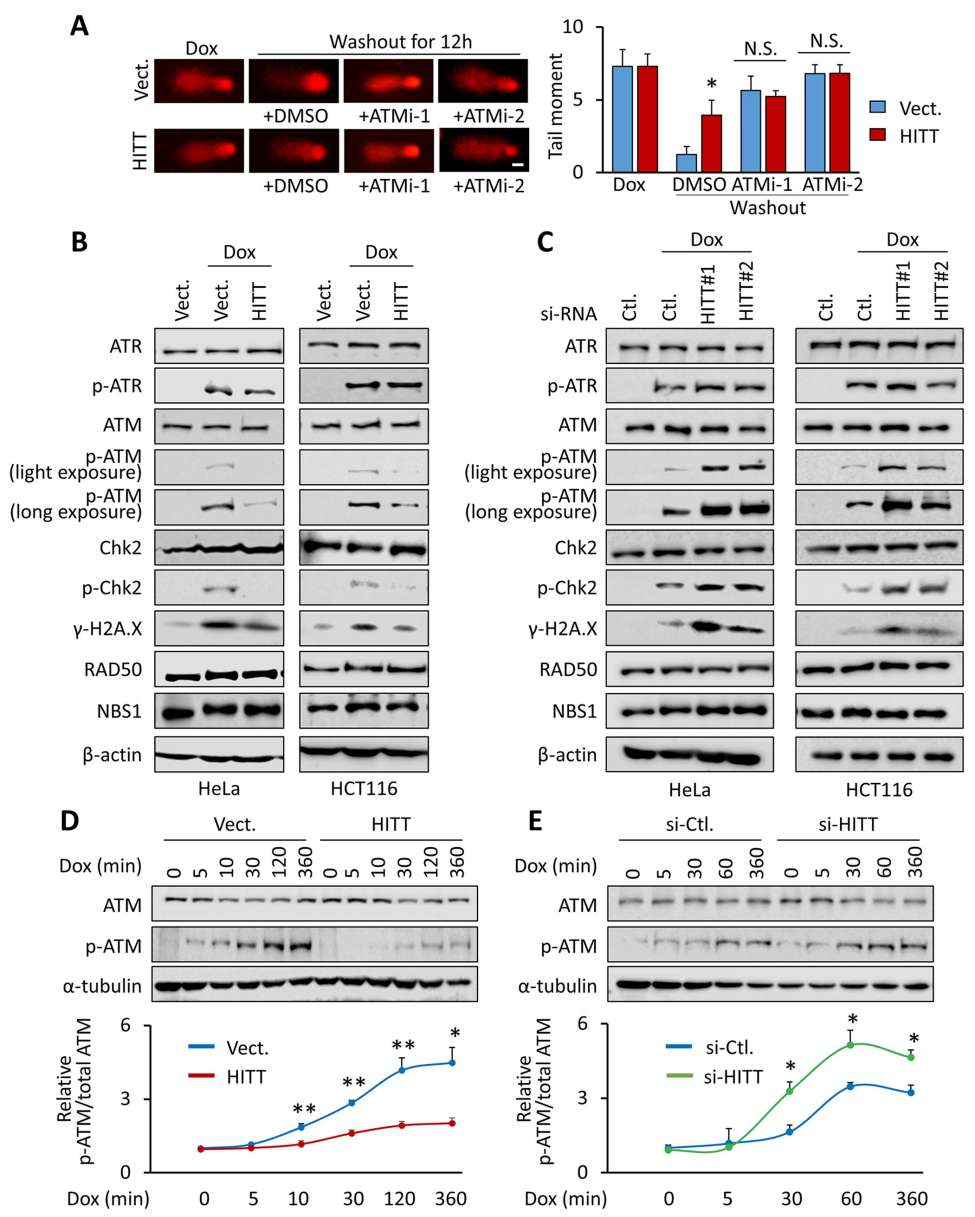
HITT reduces HR repair by inhibiting ATM activation. **(A)** DNA damage was monitored by comet assay after Dox treatment (1 μg/ml, 30 min) or 12 h after Dox (1 μg/ml) washout in the presence or absence of ATMi-1 (10 μM) and ATMi-2 (10 μM) (left). Tail moment per cell is analyzed as described in the Materials and methods and presented in the bar graph (right), scale bar, 10 μm. **(B, C)** The expression levels of the indicated proteins were determined by WB in HITT stable lines (B) or HITT KD cells (C) after Dox treatment (1 μg/ml, 24 h). **(D, E)** The expression levels of p-ATM and ATM were detected by WB in HITT stable lines Hela cells (D) or HITT KD cells (E) with 1 μg/ml Dox the indicated time courses. Data are derived from three independent experiments and presented as mean ± SEM in the bar graphs (A, D, and E). Values of controls (“Ctl.”) were normalized to 1. *P < 0.05; **P < 0.01 relative to the untreated control (A, D, and E); see also S2 and S3 Figs. For the raw data, see Fig 2A, 2D, and 2E in S1 Data, Fig 2B–2E in S1 Raw Images. ATM, Ataxia-telangiectasia mutated; ATMi-1, KU-60019; ATMi-2, KU-55933; ATR, Ataxia Telangiectasia And Rad3-Related Protein; Chk2, checkpoint kinase 2; Dox, doxorubicin; HITT, HIF-1α inhibitor at translation level; HR, homologous recombination; KD, knockdown; N.S., not significant; NBS1, Nijmegen Breakage Syndrome 1; si-, small interfering; Vect., vector; WB, western blot.

We next sought to understand the mechanisms by which HITT impairs HR. ATM activation is needed for the initiation of DSB repair by HR [38]. In line with this widely accepted notion, two independent ATM inhibitors (ATMi), KU-60019 (ATMi-1) and KU-55933 (ATMi-2), significantly reduced DNA repair in general (Fig 2A) and HR (Fig 1F). Interestingly, HITT completely lost its ability to regulate HR repair in the presence of ATMi (Figs 1F and 2A). ATMi had no obvious impacts on HITT expression levels (S2A Fig). In addition, it has been recently proposed that ATM inhibits HR by repressing DNA resection [39]. In line with this idea, we found that HITT inhibits DNA resection in an ATM activity–dependent manner by assessing the accumulation of RPA2 into nuclear foci, a well-established resection marker [40] (S2B Fig). Therefore, we reasoned that HITT inhibits DSB repair by interfering with HR through blocking ATM-dependent pathways.

To this end, the activity of ATM, as indicated by the autophosphorylation of ATM at S1981 and the phosphorylation of the well-documented ATM target Chk2, was first compared in control and HITT overexpression cells. The specificity of anti-p-ATM antibody was verified by treating cells with ATMi (S2C Fig). p-ATM and p-Chk2 were induced after Dox treatment, which was largely abolished in HITT overexpression in a HITT dose-dependent manner (Figs 2B and S2D). In contrast, HITT KD mediated by two independent HITT siRNAs or HITT CRISPR/Cas9 elevated Dox-induced p-ATM and p-Chk2 (Figs 2C and S2E). The effect of HITT on ATM activity was also validated by detecting the expression levels of another ATM substrate γ-H2A.X (Fig 2B and 2C). Inhibition of HITT in HITT overexpression cells rescued Dox-induced p-ATM and p-Chk2 (S2F Fig). Neither HITT expression nor KD affected total protein levels of ATM and Chk2 (Fig 2B and 2C). In contrast to ATM, the activation of another phosphatidylinositol 3-kinase–related protein kinase family member, Ataxia Telangiectasia And Rad3-Related Protein (ATR), did not change with HITT expression (Fig 2B and 2C). Therefore, HITT specifically inhibits ATM activity.

A time-course experiment was further performed. A mild induction of p-ATM occurred as early as 5 min after Dox treatment and kept rising in a time-dependent manner with the prolonged treatment, reaching plateau 2 h after treatment (Fig 2D), whereas HITT was increased relative late, 1 h after Dox treatment (S3A Fig). Dynamic induction of p-ATM was significantly delayed and attenuated by HITT overexpression (Fig 2D) and accelerated and elevated by HITT KD (Fig 2E).

It is also noteworthy that inhibition of HITT-mediated ATM activation was not specific to cell type or p53 status, with similar phenomena observed in p53 null H1299 and SW620 cells (S3B Fig). Similar to what was observed upon Dox treatment, HITT-inhibited ATM activation was further confirmed in Bleo-treated HeLa and HCT116 cells (S3C and S3D Fig) and Eto-treated HeLa cells (S3E Fig). Therefore, HITT restrains p-ATM upon DSBs, and it is a common phenomenon independent of p53 status.

It is known that HR is more prevalent after DNA replication [6]; however, HITT expression was not related to cell cycle (S3F Fig). HITT overexpression did not alter the levels of bromodeoxyuridine (BrdU) (S3G Fig) or cell-cycle distribution under basal conditions (S3H Fig), suggesting that HITT suppresses HR by affecting signaling in HR pathway per se, rather than by arresting the cell cycle. Despite detecting delayed entrance into S and G2/M phases in HITT overexpression cells upon Dox treatment compared with control cells, ATMi completely abolished this effect (S3H Fig). Thus, we deduced that the retarded S and G2/M phase entrance in HITT overexpression cells was due to decreased ATM activation, which is consistent with previous reports that ATM regulates cell-cycle progression [41].

These data collectively suggest that HITT is an important inhibitor of ATM activation and therefore plays essential roles in attenuating HR repair upon DSBs.

### HITT inhibits MRN-mediated ATM recruitment to the DSB sites

**Fig 3:**
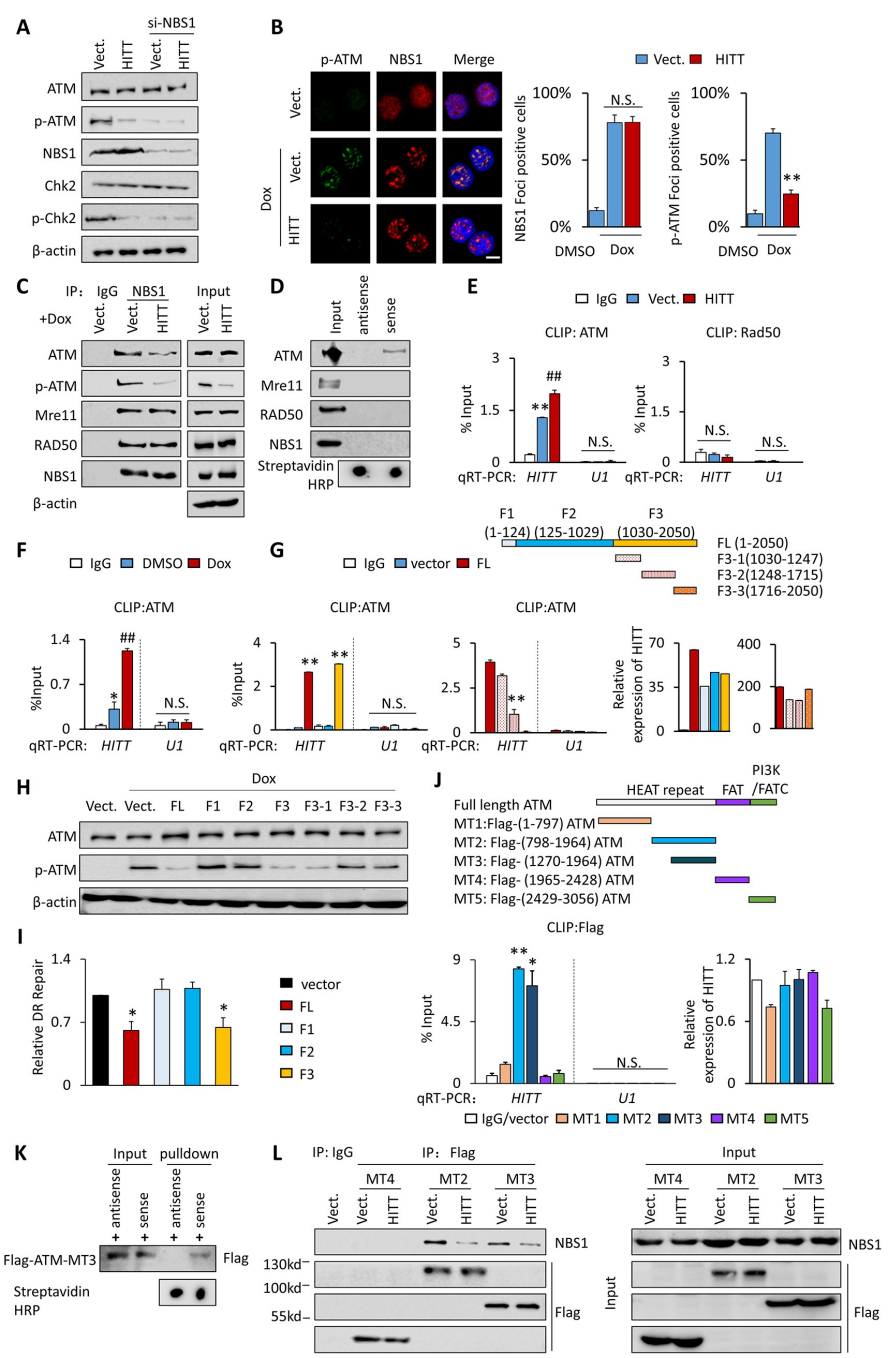
HITT binds to ATM and prevents its association with NBS1. **(A)**, ATM, p-ATM, Chk2, p-Chk2, and NBS1 protein levels were analyzed in HITT stable HeLa cells after si-mediated NBS1 KD in the presence of 1 μg/ml Dox. **(B)** The expression levels and patterns of NBS1 and p-ATM were determined by immunofluorescence staining after treatment of Dox in HITT stable HeLa cells. Representative images are presented (left). The average rates of NBS1 or p-ATM nuclear foci-positive cells were counted and are presented in the bar graph (right), scale bar, 10 μm. **(C)** The interactions of NBS1 with ATM, p-ATM, and MRN components Mre11 and RAD50 were determined by co-IP assay in vector (“Vect.”) control and HITT stable HCT116 cells upon treatment with Dox (1 μg/ml, 24 h). **(D)** ATM, Mre11, RAD50, and NBS1 levels in protein complexes pulled down by Biotin-HITT or Biotin-antisense-HITT from whole-cell extracts of HeLa cells were determined by in vitro RNA pull-down assay combined with WB. **(E, F)** HITT levels were determined by real-time RT-PCR following ATM or RAD50 CLIP in the control and HITT stable HeLa cells (E), or in HeLa cells before and after Dox (1 μg/ml, 24 h) treatment (F). IgG and U1 RNA were used as controls. **(G)** A CLIP assay was used to detect the binding of ATM with ectopically expressed full-length or HITT fragments, as indicated in the diagram, in 4T1 (a cell line without endogenous HITT expression). **(H)** ATM and p-ATM expression levels were analyzed by WB after introducing HITT or HITT fragments, as indicated, into HeLa cells followed by Dox (1 μg/ml, 24 h) treatment. **(I)** HR efficiency of ISce-I-induced DSBs in U2OS cells containing DR-GFP was determined by measuring GFP-positive cells by flow cytometry (FACS) after overexpression of full-length or fragmented HITT in HeLa cells. **(J)** HITT levels were determined by real-time RT-PCR following Flag CLIP in HeLa cells after transfection of Flag-tagged MT1–MT5, as indicated in the diagram shown above. IgG and U1 RNA were used as controls. **(K)** Direct interactions between ATM and HITT were verified by in vitro RNA pull-down assay using in vitro–translated Flag-ATM-MT3 and in vitro–synthesized HITT. **(L)** The interaction between ATM truncates and NBS1 was determined by a co-IP assay in HITT stable HeLa cells and corresponding controls after introducing the indicated ATM truncates. Data are derived from three independent experiments and represented as mean ± SEM in the bar graphs. *P < 0.05; **P < 0.01 (B, E-G, I, and J). ##*P* < 0.01; relative to vector control or DMSO control (E and F). See also S4 Fig. For the raw data, see Fig 3B, 3E-3G, 3I, and 3J in S1 Data and Fig 3A, 3C, 3D, 3H, 3K, and 3L in S1 Raw Images. ATM, Ataxia-telangiectasia mutated; ATR, Ataxia Telangiectasia And Rad3-Related Protein; Chk2, checkpoint kinase 2; CLIP, UV cross-linking and immunoprecipitation; Dox, doxorubicin; DR, Direct Repeat; DSB, double-strand break; FACS, fluorescence-activated cell sorting; GFP, green fluorescent protein; HITT, HIF-1α inhibitor at translation level; HR, homologous recombination; HRP, horseradish peroxidase; IgG, immunoglobulin G; IP, immunoprecipitation; KD, knockdown; Mre11, Meiotic Recombination 11; MRN, MRE11-RAD50-NBS1; MT, mutant type; N.S., not significant; NBS1, Nijmegen Breakage Syndrome 1; PI3K, phosphatidylinositol 3-kinase; qRT-PCR, quantitative reverse transcription PCR; si-, small interfering; Vect., vector; WB, western blot.

We next investigated the underlying mechanisms by which HITT inhibits ATM activity. MRN is a sensor of DSBs and integral to ATM-dependent DNA repair signaling [42]. NBS1 is an MRN component that plays key roles to recruit ATM to the DSB sites. As expected, NBS1 KD reduced ATM activity. Interestingly, despite no obvious impacts on HITT levels (S4A Fig), NBS1 KD completely abolished the effect of HITT on ATM activity (Fig 3A). These data suggest that HITT inhibition of ATM is dependent on MRN action. However, neither HITT overexpression nor KD produced a significant impact on MRN proteins (Fig 2B and 2C). In addition, cell-staining and chromatin fractionation assays revealed that HITT does not influence the formation of nuclear foci by NBS1 (Fig 3B) and RAD50 (S4B Fig) or the association of MRN components (NBS1 and RAD50) with chromatin (S4C Fig). In contrast, DNA damage–induced p-ATM foci were barely detectable in HITT overexpression HeLa cells (Figs 3B and S4B). In agreement, the chromatin-associated ATM was decreased with HITT overexpression and increased with HITT KD, whereas the association of chromatin with ATR was not affected by either HITT expression or KD (S4C Fig). These data suggest that HITT may inhibit ATM activation by regulating MRN-mediated ATM recruitment to sites of DNA damage. Indeed, a coimmunoprecipitation (co-IP) assay revealed that the association of p-ATM or ATM with NBS1, a key component of MRN that has been reported to be directly associated with ATM [43], was detected and was dramatically decreased upon ectopic HITT expression; however, the association of NBS1 with another MRN component, RAD50, remained unaffected (Fig 3C). Therefore, HITT appears to specifically interfere with the association of ATM with MRN upon DSBs.

### HITT is physically associated with ATM at an essential NBS1-binding site

We next asked how HITT inhibits the association between ATM and NBS1. lncRNA may act by interacting with proteins. We have previously shown that HITT is distributed both in the cytoplasm and in the nucleus [31] and therefore wondered whether HITT binds with ATM or MRN. To this end, an in vitro RNA-binding assay was first applied. As shown, the assay revealed that ATM coprecipitated with Biotin-HITT but not Biotin-antisense-HITT (Fig 3D), whereas none of the MRN components bound with HITT or antisense-HITT (Fig 3D). UV cross-linking and immunoprecipitation (CLIP) is standard in RNA research used to determine the direct interactions between proteins and nucleic acids. As revealed by CLIP assay, HITT was present in ATM, but not immunoglobulin G (IgG) or RAD50, immunoprecipitates (Fig 3E). The association between ATM and HITT was not only significantly enhanced with ectopic HITT expression but also increased with Dox treatment (Fig 3E and 3F). These data suggest that HITT is physically and specifically associated with ATM, which may be biologically significant in regulating the DDR.

We further investigated what the molecular mechanism by which HITT binds with ATM is. HITT does not have a homolog in mice, which thus provides a convenient tool to map the HITT sequence that contributes to its association with ATM, without interference from endogenous HITT. Different HITT fragments (F) were generated and similar levels of HITT were introduced into the mouse cell line 4T1. CLIP of endogenous ATM revealed that comparable rates of FL and F3 were present in ATM immunoprecipitates, whereas F1 and F2 were unlikely to contribute to the interaction (Fig 3G). The binding sites within F3 were further narrowed down to F3–1 (1,030–1,247 bp) (Fig 3G). In line with this result, we found that F3 (F3–1), but not the other fragments, inhibited ATM activity (Fig 3H). Full-length HITT and F3 elicits similar effect in inhibiting HR efficiency (Fig 3I).

We also examined which domain in ATM contributes to the interaction with HITT. To answer this question, we generated different ATM truncations as indicated in Fig 3I. ATM mutant types (MTs) that lost the MT3 sequence within the HEAT repeat domain failed to bind with HITT (Fig 3J). The direct interaction between HITT and ATM-MT3 was further verified by in vitro RNA pull-down assay using in vitro–translated ATM-MT3 protein and in vitro–synthesized sense-HITT (Fig 3K). Interestingly, MT3 has been identified to be an essential NBS1-binding site [43], suggesting HITT may mask the NBS1-binding sites, leading to the repressed association between ATM and NBS1. In agreement, we found that NBS1 bound with MT2 and MT3, which contain NBS1-binding domain, but not with MT4. Such interaction was dramatically reduced in cells overexpressing HITT compared with control cells (Fig 3L).

It is thus logical to propose that HITT binds the NBS1-binding site in ATM (MT3) via F3–1 (1,030–1,247 bp) and prevents NBS1-mediated ATM recruitment to DSBs.

### HITT is transcriptionally activated by the induction of EGR1 upon DSBs

**Fig 4:**
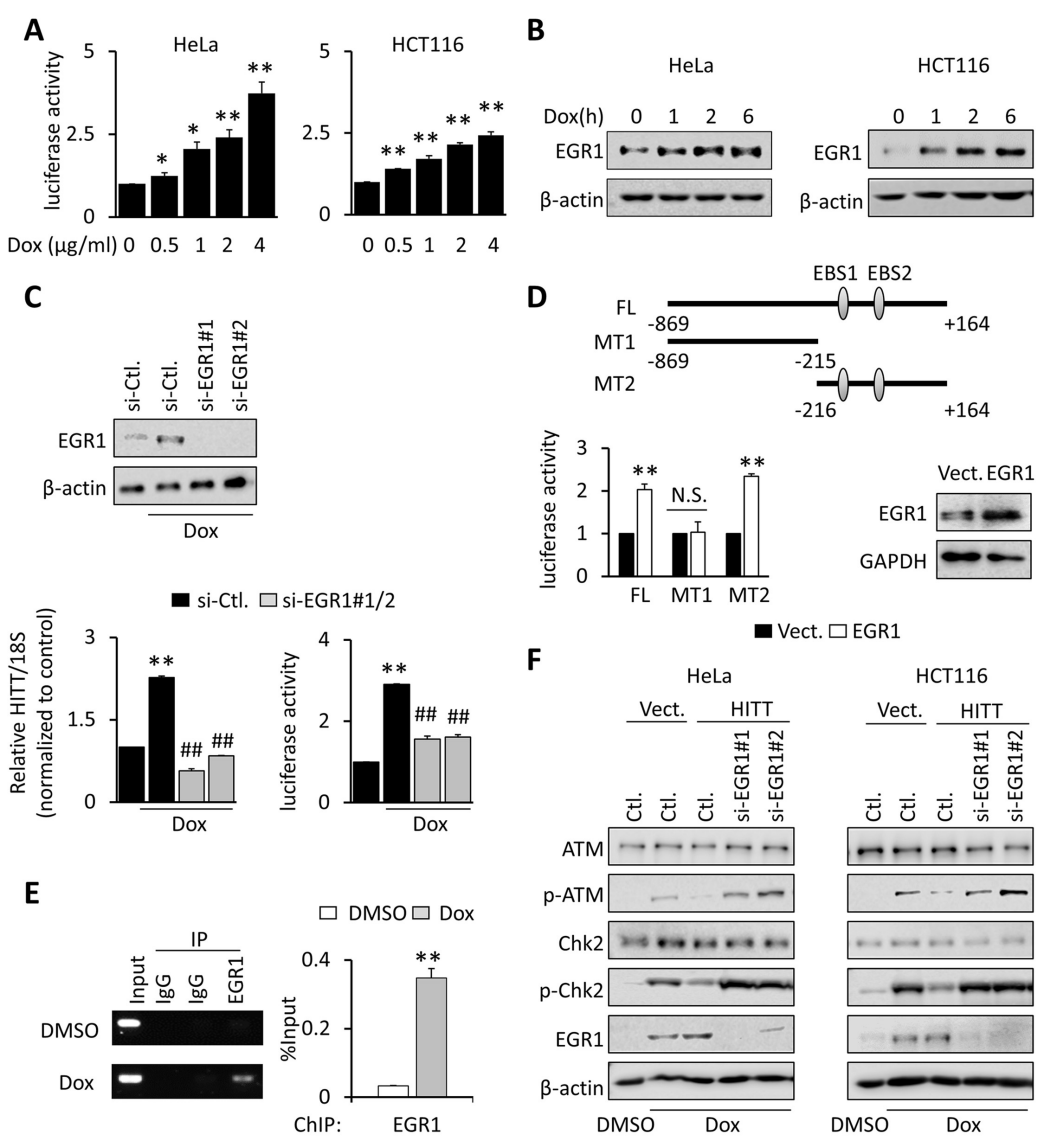
HITT is transcriptionally activated by EGR1 upon DSBs. **(A)** Relative pGL3-HITT-promoter-luc activities were determined by luciferase reporter assay in HeLa and HCT116 cells treated with different concentrations of Dox as indicated in the figure. **(B)** EGR1 levels were analyzed by WB in HeLa and HCT116 cells after Dox (1 μg/ml) treatment for the indicated time periods. **(C)** HITT expression level and pGL3-HITT-promoter-luc activity were determined by real-time RT-PCR or luciferase reporter assay in EGR1 KD cells. EGR1 KD efficiency were validated by WB. **(D)** Relative pGL3-HITT (FL or MT)-promoter-luc activity was determined by luciferase reporter assay after transfection with the indicated reporter plasmids together with EGR1 constructs. **(E)** Interaction of EGR1 with the HITT promoter was determined by ChIP assay after treating HeLa cells with or without Dox (1 μg/ml, 6 h). PCR band intensities were quantified using ImageJ and presented in the bar graph (right). (**F)** p-ATM and EGR1 levels were analyzed by WB in HITT stable lines or vector (“Vect.”) controls (“Ctl.”) (HeLa, left and HCT116, right) with the indicated transfection and/or Dox (1 μg/ml, 24 h) treatment. Data are derived from three independent experiments and represented as means ± SEM in the bar graphs. Values in control cells were normalized to 1. *P < 0.05; **P < 0.01; compared with vector or siRNA control. (A, C, D, and E). ##*P* < 0.01; relative to the Dox-treated control (C). See also S5 Fig. For the raw data, see Fig 4A, 4C–4E in S1 Data, Fig 4B–4D, 4F in S1 Raw Images. ATM, Ataxia-telangiectasia mutated; ChIP, chromatin immunoprecipitation; Chk2, checkpoint kinase 2; Dox, doxorubicin; DSB, double-strand break; EBS, EGR1 Binding Site; EGR1, Early Growth Response 1; GAPDH, glyceraldehyde 3-phosphate dehydrogenase; HITT, HIF-1α inhibitor at translation level; KD, knockdown; MT, mutant type; N.S., not significant; RT-PCR, reverse transcription PCR; si-, small interfering; WB, western blot.

Considering the functional significance of HITT in regulating the response to DSBs, we next explored the underlying mechanisms of HITT up-regulation in response to chemotherapeutic treatment. Actinomycin D, an RNA synthesis inhibitor, completely abolished Dox-induced HITT expression, suggesting that HITT up-regulation is not due to RNA stabilization (S5A Fig). Interestingly, an approximately 2- to 3-fold increase of HITT luciferase activity was detected after Dox treatment in a dose-dependent manner (Fig 4A), which was similar to the extent of HITT induction, suggesting that the up-regulation of HITT is predominately due to the promoted RNA synthesis.

By searching potential binding motifs of transcription factors located at promoter regions of HITT in the UCSC Genome Browser chromatin immunoprecipitation (ChIP) sequencing (ChIP-seq) database, the most potent transcription factors were Early Growth Response 1 (EGR1) and TATA-box binding protein associated factor 1 (TAF1) (S5B Fig), both of which have been implicated in the DDR previously [44,45]. However, the expression of EGR1, but not TAF1, was found to be elevated by Dox (Figs 4B and S5C); thus, EGR1 was subjected to the further analysis. As shown, induction of EGR1 appeared 1 h after Dox treatment, was further elevated with prolonged incubation until 6 h (Fig 4B). EGR1 by specific siRNAs abrogated Dox-induced HITT luciferase reporter activity and HITT expression (Fig 4C). These data support the idea that EGR1 is required for Dox-induced HITT transcription.

We further investigated whether HITT is a direct target of EGR1. First of all, we found that transfection with increasing doses of EGR1-expressing plasmids led to increasing HITT expression (S5D Fig). Two independent siRNAs specifically targeting EGR1 diminished the expression of the target and also reduced HITT expression (S5E Fig). Additionally, consistent with bioinformatics prediction, the MT1 reporter, which did not have the predicted EGR1-binding sites, failed to respond to EGR1 expression (Fig 4D). In contrast, the activity of the MT2 mutant reporter was as effective as the wild-type reporter in response to EGR1 (Fig 4D). A ChIP assay further revealed that EGR1 was associated with the HITT promoter at the corresponding sites, and such interaction was significantly promoted by Dox (Fig 4E). Therefore, HITT is a direct target of EGR1, which is critical for maintaining high levels of HITT after exposure to DNA damage. In line with this notion, siRNA-mediated EGR1 KD abolished Dox-induced HITT expression and facilitated ATM activation (Figs 4F and S5F).

Collectively, EGR1 is required for HITT induction upon DSBs.

### HITT down-regulation may contribute to ATM activation in vivo in human colon cancers

Given the significance of HITT in regulating ATM’s activity, we further investigated their association in vivo in human colon cancers. HITT levels were commonly decreased in colon cancer tissues to approximately 32% of their paired adjacent normal controls (*n* = 40 pairs, S6A Fig). p-ATM was increased in 27 out of 40 colon cancer tissues when compared with their matched controls (S6B and S6C Fig), whereas total ATM was not changed in cancer (S6D Fig). Although NBS1 levels were also increased in a subset of colon cancer tissues (29/40, S6E Fig), no significant association between NBS1 overexpression and p-ATM enhancement was observed (S6F Fig). Interestingly, the fold of p-ATM induction was significantly higher in the lower HITT expression group than in the higher expression group (S6G Fig). p-ATR level, also an indicator of DNA damage, was increased in colon cancers, whereas it was not related to HITT fold-change (S6B, S6H, S6I and S6J Fig). These clinical data, combined with those of our in vitro studies, suggest that HITT down-regulation may contribute to the activation of ATM in vivo.

### HITT sensitizes Dox-induced apoptosis by inhibiting ATM activation both in vitro and in vivo

**Fig 5:**
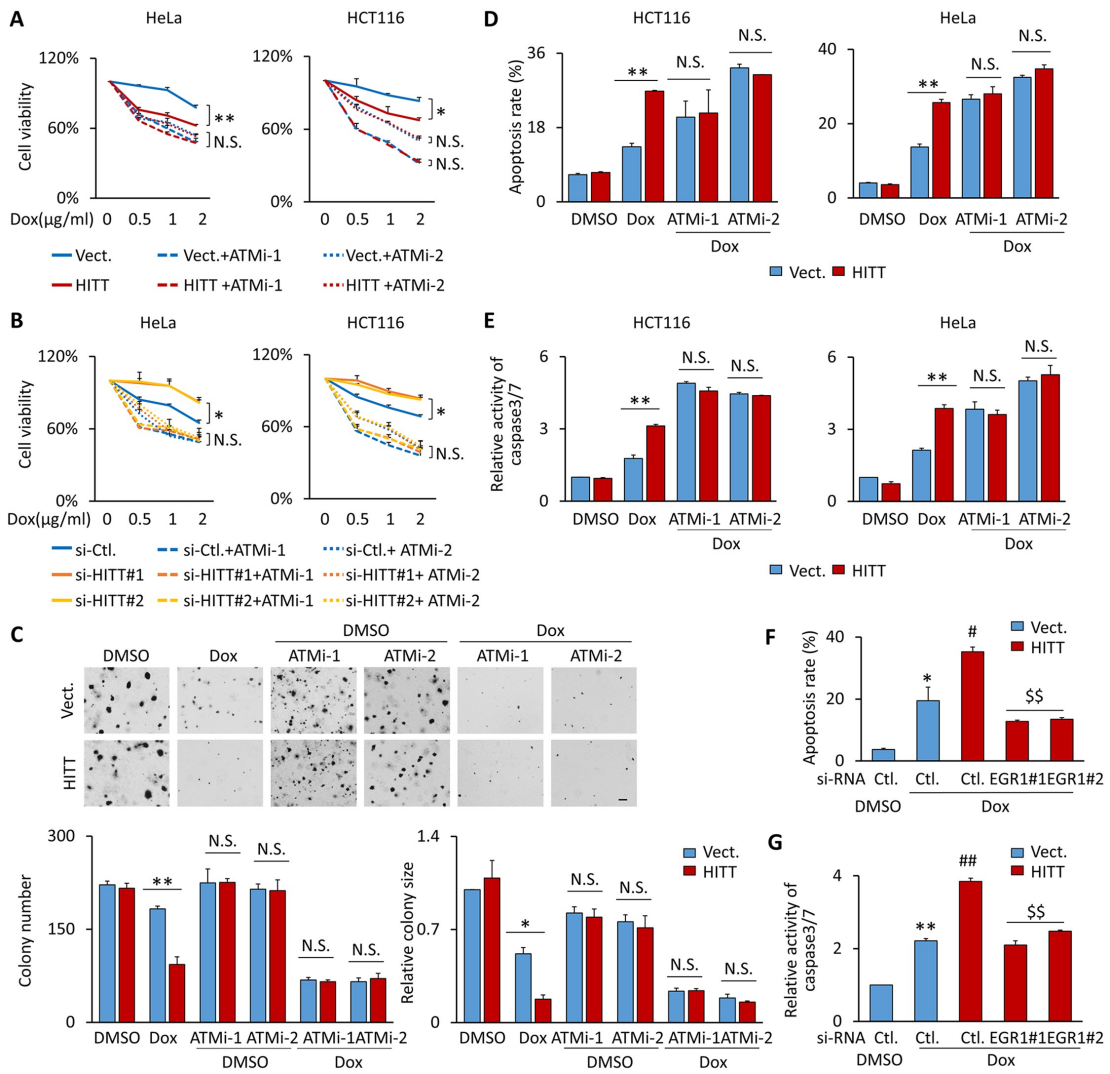
HITT sensitizes Dox-induced apoptosis by inhibiting ATM in vitro. **(A, B)** The survival rates of cells with the indicated treatments with or without 10 μM ATMi-1 and ATMi-2 were determined by MTT assay. **(C)** The survival of cells grown on soft agar was determined by clonogenic survival assay with the indicated treatments in control (“Ctl.”) and HITT stable HeLa cells, scale bar, 100 μm. **(D-G)** Apoptosis levels revealed by Annexin V staining (D, F) or caspase 3/7 activity assay (E, G) in vector (“Vect.”) and HITT stable HCT116 and HeLa cells after the indicated treatments with Dox (1 μg/ml) or 10 μM ATMi-1 and ATMi-2. Data are derived from three independent experiments and represented as mean ± SEM in the bar graphs. Values in control cells were normalized to 1 (A, B, E, G). *P < 0.05; **P < 0.01 (A-G); #*P* < 0.05; ##*P* < 0.01; relative to Dox-treated control (F, G); $ $*P* < 0.01; compared with Dox-treatment HITT stable line (F, G). See also S7 Fig. For the raw data, see Fig 5A–5G in S1 Data. ATM, Ataxia-telangiectasia mutated; ATMi-1, KU-60019; ATMi-2, KU-55933; Dox, doxorubicin; EGR1, Early Growth Response 1; HITT, HIF-1α inhibitor at translation level; MTT, 3-(4,5-dimethylthiazol-2-yl)-2,5-diphenyltetrazolium bromide; N.S., not significant; si-, small interfering.

Many chemotherapeutic drugs remove cancer cells by inducing severe DNA damage. The activation of ATM and the subsequent DNA damage repair can limit the effectiveness of DNA-damaging reagents [32]. Considering the important function of HITT in regulating ATM activity and the DDR, its effects on chemotherapy-induced cell death were explored using the 3-(4,5-dimethylthiazol-2-yl)-2,5-diphenyltetrazolium bromide (MTT) assay. Our results show that cell viability was reduced by Dox in a much more dramatic manner in HITT stable lines than in corresponding vector controls (Fig 5A), whereas HITT KD dramatically alleviated Dox’s inhibitory effect on cell viability (Fig 5B). ATMi-1/2 reduced cell viability in the presence of Dox and also completely abolished the impact of HITT (Fig 5A and 5B). Supportively, HITT F3, which is shown to contribute to ATM activity and HR inhibition, produced similar effect to increase Dox-induced cell death (S7A Fig). Under unstressed basal conditions, HITT had no obvious effect on clonal number and size when cells were grown on soft agar, whereas Dox treatment produced a much more dramatic effect on the inhibition of clonal growth in HITT-overexpressing cells than in controls (Fig 5C). Furthermore, HITT significantly elevated apoptosis rates, as indicated by Annexin V–positive staining and cleaved caspase 3/7 after Dox treatment in both HeLa and HCT116 cells (Fig 5D and 5E). The effects of HITT on clonal formation and apoptosis were completely abolished by ATMi (Fig 5C and 5E). KD HITT regulators, EGR1, inhibited Dox-induced apoptosis (Fig 5F and 5G). Similar results were obtained in Bleo- and Eto-treated HCT116 and HeLa cells (S7B–S7E Fig) and Dox-treated p53-null H1299 cells (S7F and S7G Fig). These data collectively suggest that HITT facilitates DNA damaging agent–induced apoptosis by inhibiting ATM in a p53-independent manner.

**Fig 6:**
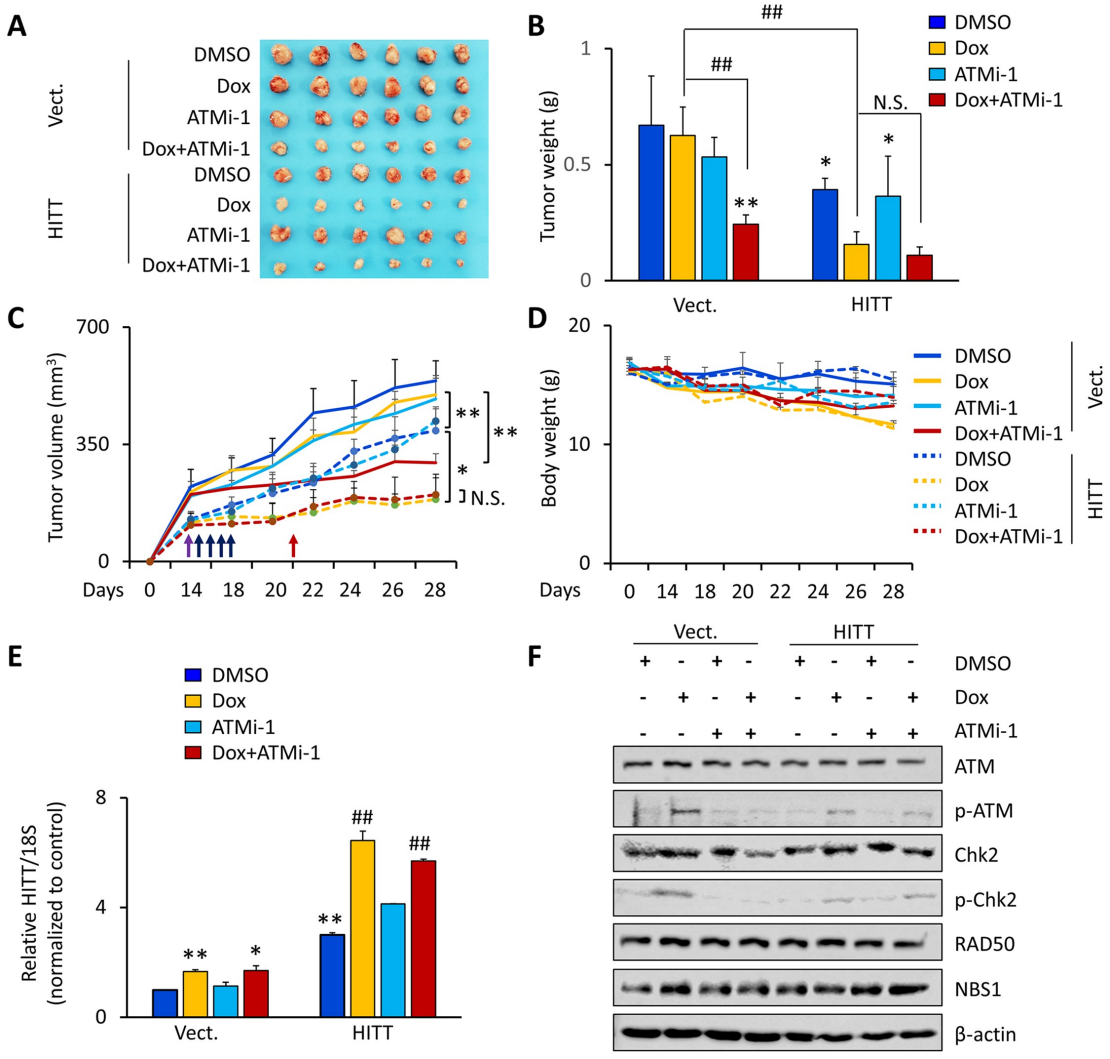
HITT axis sensitizes Dox-induced apoptosis by inhibiting ATM in vivo. **(A-C)** Tumor images (A) and tumor weight at day 28 (B), as well as tumor volumes at the indicated time points (C) of HCT116 vector (“Vect.”) and HITT stable cells were injected to the nude mice; when the tumor volume reached about 200 mm^3^, the mice were randomly divided into four groups and subjected to the treatment of DMSO and Dox alone (10 mg/kg, intraperitoneal) once per week for 2 wk or in combination with ATMi-1 (60 mg/kg, intraperitoneal) daily for 5 d. The average values are present in the bar graphs (mean ± SD) (J) (*n =* 6 for each group). **(D)** The body weights of different groups of mice measured at the time points when tumor volumes were measured. **(E, F)** HITT levels were measured by real-time RT-PCR (E) and expression levels of the indicated proteins were determined by WB (F) in samples derived from HCT116 xenografts. *P < 0.05; **P < 0.01 (B, C, and E). ##*P* < 0.01; compared with HITT stable xenograft DMSO group (E). For the raw data, see Fig 6B-E in S1 Data, Fig 6F in S1 Raw Images. ATM, Ataxia-telangiectasia mutated; ATMi-1, KU-60019; Chk2, checkpoint kinase 2; Dox, doxorubicin; HITT, HIF-1α inhibitor at translation level; N.S., not significant; NBS1, xxxx; RT-PCR, reverse transcription PCR; si-, small interfering; WB, western blot.

The impact of HITT on the effect of Dox was also evaluated in vivo using a nude mice xenograft model. To avoid big initial tumor volume differences on HITT overexpression and control xenografts before Dox treatment, more HITT-overexpressing HCT116 cells than vector controls were inoculated into nude mice. The mice with similar tumor volumes (about 200 mm^3^) were subjected to the indicated treatments (*n* = 6 for each group). Even more HCT116/HITT cells were injected, with the growth rates of HITT xenografts still slightly lower than those of vector controls (Fig 6A–6C). Dox treatment only slightly reduced tumor growth in the vector control xenografts, and no statistical significance was obtained in comparison with the untreated vector controls (Fig 6A–6C). Remarkably, Dox reduced tumor weight by approximately 4-fold in HITT overexpression xenografts compared with vector xenografts (Fig 6A and 6B). Furthermore, ATMi-1, which has been tested in preclinical [46], produced a mild inhibitory effect on tumor growth (Fig 6A–6C). Consistent with previous reports, the effect of ATMi-1 was more evident when combined with the DNA-damaging agent Dox. Interestingly, no synergistic effect was observed with the combination of HITT overexpression and ATMi-1 (Fig 6A–6C), which further supports the idea that HITT inhibits tumor growth by preventing the activation of ATM. Dox treatment led to a mild decrease of body weight in mice (Fig 6D).

In agreement with the in vitro data, HITT was elevated by Dox in vivo (Fig 6E). Western blot (WB) assays revealed that ATM activity was increased by Dox and was diminished by HITT (Fig 6F). ATMi-1 produced a similar effect with ectopic HITT expression on ATM activity. Neither HITT expression nor ATMi-1 had an obvious effect on the expression of total ATM and MRN components.

## Discussion

DNA repair inhibitors have been developed as clinical agents to reverse radiation or genotoxic drug resistance [32]. Basic research into understanding the regulatory mechanisms underlying DNA damage repair is crucial for the identification of tumor markers, to allow for more-effective targeted cancer treatment.

**Fig 7:**
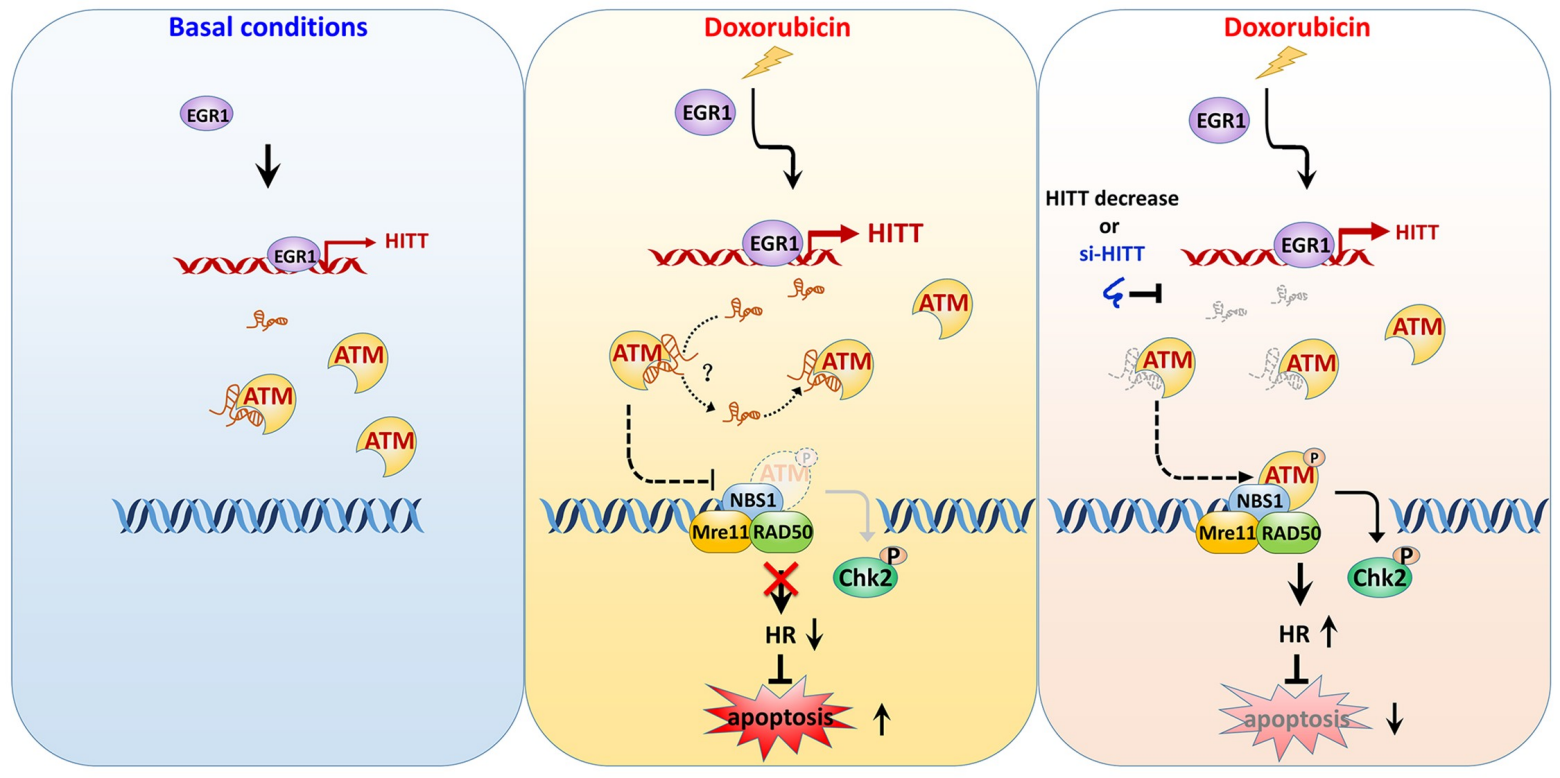
Proposed model of HITT regulation of ATM activity in cancer cells. Under unstressed conditions, EGR1 contributes to the basal expression levels of HITT, which can be directly associated with ATM. In response to DSBs—e.g., following Dox treatment—EGR1 is elevated and then play roles in promoting HITT expression by binding at particular regions of the HITT promoter. Elevated HITT may transiently bind ATM at the HEAT repeats domain at the typical NBS1-binding sites, via bases 1,030–1,247. Such activity of HITT changes the ability of ATM in binding with MRN complex, leading to reduced HR and the promotion of apoptosis. si-HITT-mediated HITT inhibition or HITT down-regulation facilitates ATM recruitment by NBS1 to the sites of DSBs, leading to ATM autophosphorylation and optimal activation followed by effective HR, which prevents server DNA damage and apoptosis. ATM, Ataxia-telangiectasia mutated; Chk2, checkpoint kinase 2; Dox, doxorubicin; DSB, double-strand break; EGR1, Early Growth Response 1; HITT, HIF-1α inhibitor at translation level; HR, homologous recombination; Mre11, Meiotic Recombination 11; MRN, MRE11-RAD50-NBS1; N.S., not significant; NBS1, Nijmegen Breakage Syndrome 1; si-, small interfering.

Here, we identify a novel intrinsic inhibitor of ATM activation that is a key and apical event in initiating DDR cascades. Unlike previous reported ATM regulators, this molecule brake is an RNA, namely HITT, that specifically and physiologically binds with ATM. Upon DSBs, HITT expression is elevated and its association with ATM is increased accordingly, thereby retraining MRN-mediated recruitment of ATM to the DNA damage sites and compromising DNA repair mediated by HR (Fig 7). Two independent ATMi completely abrogates HITT’s effect on the DDR program and HR. HITT does not further chemosensitize cells treated with ATMi, supporting the idea that HITT mainly interferes with the same prosurvival signals as ATMi.

Of note, no less important than the activation of the ATM-mediated DDR network is to limit its activation after DNA damage repair. This process may also be complex and highly structured. The mechanisms underlying ATM inhibition have emerged recently. It has been shown that H1.2 and Bridging Integrator 1 (BIN1) attenuate ATM activation by directly interacting with ATM [47] or by indirect mechanisms involving E2F1 [48]. Notably, HITT expression is elevated 1 h after Dox treatment, which is later than ATM activation is detected (5 min). It is reasonable to propose that cells limit ATM from overactivation by enhancing HITT after DNA damage repair. HITT may represent an important brake in controlling the ATM-mediated DDR network, which is a key cell fate determinant, particularly upon prolonged exposure to DNA-damaging agents in cancer treatment. We also made an interesting observation that HITT binds with ATM under basal conditions (Fig 7), suggesting that it may provide quality control to counteract toxic HR. Indeed, a deficiency in ATM inhibition has been associated with carcinogenesis, resistance to DNA damage–induced cancer treatment, and loss of mitotic checkpoint [49–51]. It is reasonable to propose that the expression of HITT may alleviate and loss of HITT expression may reinforce such effects. In addition, it has become apparent that ATM is active in response to multiple conditions involved in the maintenance of cellular homeostasis [7]. It would be interesting to investigate the physiological significance of the newly identified HITT-ATM interaction in a DNA damage–independent context.

MRN is a sensor for cells to recognize DSBs [52]. Recruitment and optimal activation of ATM is mainly dependent on its interaction with the MRN component NBS1 [43]. Consistent with this notion, the mutation of ATM-binding sites in NBS1 completely abrogates ATM accumulation at DSB sites [43]. Interestingly, the regulation of ATM by HITT is MRN(NBS1)-dependent. We further defined the ATM site that facilitates HITT binding to a previously identified NBS1-binding site (the HEAT repeats domain), raising the possibility that HITT competes with NBS1 in binding with ATM. Indeed, ectopic HITT expression led to increased association of HITT/ATM and reduced association of NBS1/ATM, without influencing total ATM or NBS1 protein levels. Also notably, NBS1 is essential to induce ATM monomerization and activation [13,53]. HITT and NBS1 bind with ATM at the same sites, although eliciting opposite effects on ATM activity. It remains to be investigated whether HITT plays roles in influencing ATM monomerization and, if so, whether such activity of HITT is dependent on its inhibitory effect on NBS1/ATM interaction.

Notably, lncRNA is normally expressed at lower levels than protein-coding genes [54]. One potential explanation for the robust effect of HITT on ATM activity is that HITT may transiently interact with ATM to modulate ATM modification to render it inactive. Indeed, it has been reported that ATM modification may influence its activation [20,55]. In addition, although HITT itself may have no catalytic activity, its effect on ATM downstream signaling can be amplified and is not necessarily linearly associated with its expression levels. It will be interesting to test the above models in future studies.

We also suggest that HITT’s inhibition of ATM may be of clinical significance. A small peptide containing the evolutionarily conserved region of NBS1, which binds with ATM, disrupts the interaction between NBS1 and ATM. This peptide also prevents DNA damage signaling and radiosensitization of cells, underscoring the notion that blocking the NBS1/ATM interaction is a potential target for anticancer treatment [56]. Indeed, we found that HITT-mediated ATM inhibition leads to increased cell death when cells are treated with Dox both in vitro and in vivo. Because of its significant effects regarding improving chemosensitivity, our data encourage the future development of lncRNA-based cancer therapies for patients resistant to genotoxic treatments.

Furthermore, p53 is an important gene in regulating genome stability and the DDR [57]. However, we made an interesting observation that the induction of HITT upon DSB- and HITT-dependent DDR activity is p53-independent. This is important, as p53 is the most commonly mutated tumor suppressor gene in human cancers [58]. The discovery of a p53-independent function of HITT implies that HITT could be utilized to sensitize cells to cytotoxic drug treatments that are applicable to a wide range of human cancers, regardless of their p53 status.

Moreover, HITT expression is commonly decreased, whereas p-ATM levels are increased, in colon cancer tissues. Despite being elevated in a subset of cancer samples, NBS1 overexpression is not likely to be important for ATM activation, which suggests that additional layers of regulation exist for NBS1-mediated ATM activation. Importantly, the extent of the reduction of HITT expression is significantly associated with increasing p-ATM, which suggests that HITT down-regulation may contribute to the activation of p-ATM in vivo. This is important, because ATM activation has been associated with treatment failure in multiple cancers. Detection of ATM variance or its protein expression has been suggested to be a potential predictive marker for drug response [5], and our findings provide an effective marker, HITT, for the prediction of ATM activity. In addition, BRCA1- and BRCA2-mutated cells are defective in HR repair and are thus highly sensitive to poly ADP-ribose polymerase (PARP) inhibitor treatment [12]. In addition, ATM deficiency has been shown to increase sensitivity to PARP and ATR inhibitors [59]. HITT specifically inhibits the ATM-dependent HR pathway. The synergistic effects of HITT and PARP inhibitors warrant future investigation. ATMi has also been shown to be a promising single agent for the treatment of human cancers with defects in particular oncogene signals, such as a loss of PTEN (phosphate and tension homology deleted on chromosome ten) [60]. Further work is thus necessary to decipher whether HITT could be applied alone in subtypes of cancers.

We have also found that Dox-induced HITT up-regulation is predominately controlled by EGR1. EGR1 can be rapidly up-regulated by a wide variety of extracellular stimuli, including the activation of growth and differentiation signals, tissue injury, and apoptotic signals, such as IR. Despite its transient activation, the expression of EGR1 is likely required for HITT up-regulation, since EGR1 KD completely abolishes Dox-induced HITT. These data are consistent with the fact that HITT is involved in regulating the initiation steps of the DDR. In addition, EGR1 KD is accompanied by impaired DDR and p53 activation [61]. Here, we provide the first evidence that EGR1 exerts its effect on the DDR by modulating lncRNA expression. However, HITT is maintained at high levels, even when EGR1 expression drops back down to unstressed levels, suggesting that additional transcriptional regulation of HITT also exists. Indeed, a number of additional transcription factors have been indicated to bind with HITT promoter as shown in UCSC Genome Browser ChIP-seq database. It will be interesting to investigate the impacts of these candidate transcription factors on HITT expression in the context of DSB.

Altogether, our results demonstrate that HITT plays essential roles in inhibiting the HR pathway by restraining ATM activity and thus may be an effective adjuvant therapy for genotoxic or radiomimetic compounds or a predictive marker for treatment efficiency in cancer.

## Materials and methods

### Ethics statement

Human colorectal cancer tissues and their corresponding adjacent normal controls (40 pairs) were collected from the Fourth Affiliated Hospital of Harbin Medical University in China. Written informed consent was obtained from all patients. The study has been approved by the Research Ethics Committee of Harbin Medical University, China. All animal procedures were performed according to protocols approved by the Rules for Animal Experiments published by the Chinese Government (Beijing, China) and approved by the Research Ethics Committee of Harbin Institute of Technology, China (IACUC number: IACUC-2018005).

### Cell culture and chemicals

HeLa, SiHa, T24, and 293T cells were grown in Dulbecco’s modified Eagle’s medium (DMEM) (Gibco, Carlsbad, CA, United States of America), supplemented with 10% (v/v) fetal bovine serum (Biological Industries). HCT116, HCT116 (p53-/-), H1299, and SW620 were maintained in RPMI-1640 medium (Gibco, Carlsbad, CA, USA) supplemented with the 10% FBS. HITT sequence was subcloned into a lent-virus vector, pLnc-KP. Virus was packaged in HEK-293T cells and then infected HCT116 and HeLa cells. Single clones were selected by puromycin and the selected clones with HITT overexpressed similar to the normal cells were used to do further experiments. DR-U2OS, EJ2-U2OS, and EJ5-U2OS were kindly provided by Prof. Jeremy M. Stark [35]. All cells were grown at 37°C in the humidified incubator (Thermo Scientific) with 5% CO_2_. Cell lines were routinely tested to exclude mycoplasma contamination, and all cells have never been passaged longer than 1 mo. All drugs used in this study were as follows: Doxorubicin (Selleck, #S1208); Calicheamicin (MedChemExpress, #HY-19609); Bleomycin (Apexbio, #A8331); Etoposide (Apexbio, A1971); ATM inhibitor Ku60019 (Apexbio, #A8336); ATM inhibitor Ku55933 (MedChemExpress, #HY-12016); and Actinomycin D (MedChemExpress, #HY-17559).

### Colorectal cancer tissues samples

The specimens were collected and stored in liquid nitrogen immediately after surgery. The total proteins and RNAs were extracted and then subjected to WB and real-time RT-PCR analysis.

### In vivo xenograft mouse study

HITT expression was stably restored in HCT116 cells, by using empty vector as a control. The female nude mice between 4 and 5 wk old were purchased from Beijing HFK Bioscience. To avoid the initial big volume difference between the HITT overexpression and control groups, the 1 × 10^7^ vector cells and 1.5 × 10^7^ HITT stable HCT116 cells were inoculated subcutaneously. The tumor volumes were measured every week and calculated as length × width^2^ × 0.5. When the tumor volume reached about 200 mm^3^, the mice were randomly divided into four groups and subjected to the treatment of DMSO and Dox alone (10 mg/kg, intraperitoneal) once per week for 2 wk or in combination with ATMi-1 (60 mg/kg, intraperitoneal) daily for 5 d (*n* = 6 for each group). The tumor size and the body weights of the mice were measured every 2 d. After treatment for 2 wk, the mice were anesthetized and culled. The tumor was carefully removed, photographed, and weighed.

### WB assay

Cells or tissue specimens were lysed in UREA buffer (8 M Urea, 1 M Thiourea, 0.5% CHAPS, 50 mM DTT, and 24 mM Spermine). Same amount of proteins were separated by SDS-PAGE. After being incubated with the indicated antibodies, the immune complex on membrane was detected by an ECL kit (Thermo Scientific, #32106). Antibodies used for WB were shown as follows: Antibodies against p-ATM (S1981) (#Ab81292), ATM (#Ab32420), NBS1 (#Ab32074), and RAD50 (#Ab89) were from Abcam. Antibodies against p-Chk2 (T68) (#2197), Mre11 (#4847), ATR (#13934), and p-ATR (S428) (#2853) were from Cell Signaling Technology. Antibodies against Chk2 (#13954-1-AP), FLAG (#20543-1-AP, for WB), RAD51 (#14961-1-AP), XRCC4 (#15817-1-AP), α-tubulin (#66031-I-Ig), β-actin (#60008-1-Ig), and GAPDH (#60004-1-Ig) were obtained from Proteintech. Additional antibodies included EGR1 (sc-189, Santa Cruz), Histone3.1 (#KM9005T, Sungene), FLAG (#MCA4764, Bio-Rad, for IP), mouse IgG (#A7028, Beyotime), and Rabbit IgG (#A7016, Beyotime).

### siRNA and transfection

siRNA specifically targeting HITT, NBS1, EGR1, or nonspecific control (Ctl.) were synthesized by GenePharm and their sequences are as follows: si-Ctl (UUUUCCGAACGUGUCACGUTT); si-HITT#1 (CCAGGAAGGCGAUUUACAATT); si-HITT#2 (CCUCAUGAAUGGGAUUAAUTT); si-NBS1 (GGAGGAAGAUGUCAAUGUU); si-EGR1#1 (GGCAUACCAAGAUCCACUU); si-EGR1#2 (AGAGGCAUACCAAGAUCCA). HITT promoter sequence was amplified by PCR using HEK-293 genomic DNA as a template. DNA sequence was verified and subcloned into pGL3 vector, generating pGL3-HITT-promoter (−896 to +164), pGL3-HITT-promoter (−896 to −215), and pGL3-HITT-promoter (−216 to +164) plasmids. ATM plasmid was bought from Addgene, and all truncated ATM MTs were generated by PCR using full-length ATM as a template. The truncated ATM sequence were subcloned into pCDNA3.1-3xFlag vector. ISce-I plasmid was kindly provided by Prof. Jun Huang, Zhejiang University. siRNA and plasmids were transfected into cells by Lipofectamine 2000 (Invitrogen, #11668027).

### RNA extraction and real-time RT-PCR

Total RNA was extracted using Trizol Reagent according to the manufacturer’s instructions (Invitrogen, #15596018). cDNA was synthesized by using PrimeScript reverse transcription (RT) reagent kit (TaKaRa, #RR047A) in presence of gDNA Eriser. Quantitative real-time PCR was carried out in the ViiA7 real-time PCR (Applied Biosystems) using SYBR Premix Ex Taq II kit for Real-Time (Takara, #RR820L). The primer sequences used in RT-PCR are as follows: HITT forward 5′-ACACAAATGCTGGCCTCTGTCA-3′, reverse 5′-GGCAAGTGGCAAAGCCTCTC-3′, 18 s forward 5′-AACTTTCGATGGTAGTCGCCG-3′, reverse 5′-CCTTGGATGTGGTAGCCGTTT-3′, U1 forward 5′-GGGAGATACCATGATCACGAAGGT-3′, reverse 5′-CCACAAATTATGCAGTCGAGTTTCCC-3′.

### IP

Cells were lysed in NETN buffer (50 mM Tris-HCl [pH 8.0], 150 mM NaCl, 1% NP-40, 1 mM EDTA), with Proteinase Inhibitor Cocktail (MedChemExpress, #HY-K0010) added freshly before use. After being precleaned by protein G sepharose beads 4 Fast Flow (GE Healthcare, #17061802), specific antibodies or control IgG was added to the supernatant, which was incubated with FBS blocked beads for at least 20 h at 4°C. Beads with the bound immunoprecipitates were collected following four washes with cold NETN. The subsequent immunoprecipitates were extracted for WB assay.

### Luciferase reporter assay

After the indicated transfection, the luciferase activities were detected with the luciferase assay system (Promega, #E1910) according to the manufacture’s introduction. The relative luciferase activities were normalized with the Renilla luciferase activities.

### Apoptosis assay

After the indicated treatments, both suspended and attached cells were collected. Cell suspension in binding buffer were incubated with 5 μL Annexin V/FITC for 10 min and then with 5 μL propidium iodide (PI) (Sungene) for 5 min at room temperature in the dark. The rate of apoptosis was determined by flow cytometry.

### Caspase3/7 activity assay

Following the indicated treatments, cells were subjected to the caspase 3/7 activity assay by Caspase-Glo_3/7 Assay Systems (Promega, #G8091) according to the manufacturer’s instructions. The assay was conducted in triplicates and repeated independently for three times, which was represented as a fold increase of fluorescence calculated by comparing cells with untreated control cells.

### Comet assays

Following the indicated treatments, comet assays with EB (Sigma) staining were performed as reported previously [62]. The quantification of comet rate and tail moment were performed with CASP software (http://www.casp.of.pl).

### NHEJ assay and HR assay

DR-U2OS, EJ2-U2OS, and EJ5-U2OS cells were transfected with HITT expression plasmids or two independent siRNA oligos specifically targeted HITT, together with ISce-I plasmid. Forty-eight to 72 h after transfection, cells were harvested and resuspended in 0.5 ml of PBS (pH 7.4). GFP signal was analyzed by flow cytometry (FACS).

### ChIP

Cells were incubated with formaldehyde to yield protein-DNA cross-link complexes, which were purified and sheared by sonication. The chromatin was divided equally into two groups for further IP reaction with anti-EGR1 antibody or IgG control. The immunoprecipitates were pelleted by centrifugation and then incubated at 65°C to reverse the protein-DNA cross-linking. The DNA was extracted by the Axygen product purification kit and subjected to PCR analysis.

### CLIP

UV-irradiated cells were collected in lysis buffer (5 mM PIPES [pH 8.0], 85 mM KCl, 0.5% NP40 and 1% SDS, 10 mM EDTA, 50 mM Tris-HCl [pH 8.1]) and supplemented with Protease Inhibitor Cocktail and RNase inhibitor (Thermo Fisher). The cell lysates were precleaned with protein G sepharose beads and then incubated with the indicated antibodies or IgG control, rotating at 4°C overnight. The antibody-RNA complexes were collected. The immunoprecipitated RNA was eluted and extracted for further real-time RT-PCR analysis.

### In vitro RNA pull-down assay

Biotin-labeled HITT and its antisense were in vitro synthesized by Biotin RNA Labeling Mix (Roche, 11685597910). After treatment with RNase-free DNase I, secondary structure of Biotin-labeled RNA was recovered and incubated with streptavidin agarose beads (Invitrogen) overnight. The fresh cell lysates or in vitro–translated protein (TNT T7 Quick coupled transcription/translation system, Promega) were collected and incubated with RNA-captured beads at 4°C for 1 h. The associated proteins were detected by WB.

### Clonogenic survival assay

HITT stable HeLa cells were treated with Dox (1 μg/ml) with or without Ku60019 (10 μM) or Ku55933 (10 μM) for 24 h. The untreated and the treated cells were trypsinized and subjected to a typical clonogenic survival assay. A total of 5 × 10^4^ cells were mixed with 2×DMEM medium with 20% FBS containing 0.7% agar and then spread on the top of a bottom agar layer (1% agar in drug-free DMEM full growth medium) in a 6-well plate. DMEM medium (2 mL) was added and refreshed every 3–4 d. Cells were grown for 1 mo. Colonies were counted and photographed after stained with 1% Crystal Violet. The colony numbers and relative colony size were analyzed by ImageJ.

### Chromatin fraction

Cells were fractionated as previously described [63]. As brief, cells were resuspended in buffer A (10 mM HEPES [pH 7.9], 10 mM KCl, 1.5 mM MgCl_2_, 0.34 M sucrose, 10% glycerol, 1 mM DTT). Triton X-100 (0.1%) was added and incubated on ice for 5 min. Cell lysates were centrifuged at 1,300*g* for 4 min, and the remaining pellet (enriched with nuclei) was washed with buffer A (10 mM HEPES [pH 7.9], 10 mM KCl, 1.5 mM MgCl_2_, 0.34 M sucrose, 10% glycerol, 1 mM DTT) once and then lysed in buffer B (3 mM EDTA, 0.2 mM EGTA, 1 mM DTT) on ice for 5 min. The nuclei lysate was then centrifuged at 1,700*g* for 4 min and the supernatant (soluble nuclear fraction) was removed. The final pellet is the chromatin fraction that is ready for further analysis.

### MTT assay

Cell viabilities after the treatment were assessed by the colorimetric MTT (Sigma-Aldrich) assay. The absorbance was measured with a spectrometer at 490 nm. Each experiment was conducted in triplicates and repeated independently for three times.

### Cell-cycle synchronization assay

To synchronize the cell cultures, HeLa cells were exposed to 2.5 mM TdR (Sigma) for 16 h (first block) and then placed on fresh medium supplemented with 10% FBS for 12 h (first release). After that, cells were subjected to another round of TdR treatment (second block) before incubation with fresh medium for an additional 0, 6, 8, and 10.5 h, respectively. The cells were collected at different time points for cell-cycle analysis by flow cytometer, and HITT expression was measured by real-time RT-PCR.

### Immunofluorescence staining and BrdU incorporation assay

Cells grown on cover slips in a 24-well plate were fixed in 4% paraformaldehyde for 20 min and then treated with 0.1% Triton X-100 solution on ice for 4 min. Cells were then blocked by 3% BSA for 1 h followed by the antibody incubation at 4°C overnight. After that, cells were washed 3 × 5 min in PBS and then incubated with the fluorescently labeled secondary antibody for 1 h. DAPI was stained for 3 min for visualizing the nucleus. The slices were mounted by 90% glycerinum and images were captured by a Zeiss Axio Observer confocal microscope.

BrdU (Sigma, #B5002) incorporation assay was carried out by following the protocol provided by Cell Signaling Technology. Briefly, BrdU was diluted to a final concentration of 0.03 mg/mL with fresh DMEM and then applied onto the cells grown on slices. Cells were incubated with 1.5 M HCl followed by 5-min fixation in 70% cold ethanol. Immunostained with anti-BrdU antibody was then conducted as shown above. The antibody dilution ratio are as follows: p-ATM (#Ab81292, 1:100), p-ATM (10H11.E12) (#NB100-306, 1:1,000), NBS1 (#Ab32074, 1:100), RAD50 (#Ab89, 1:100), RPA2 (#10412-1-AP, 1:200), Goat Anti-Mouse IgG H&L (Alexa Fluor 488) (#Ab150113, 1:400), and Goat Anti-Rabbit IgG H&L (Alexa Fluor 647) (#Ab150079, 1:400).

### Trypan blue staining

After different drug treatment for 24 h, both survival and dead cells were collected and subjected to trypan blue staining and counted by hemocytometer. The percentage of dead cells (stained)/total cells was determined by counting an average of 200–500 total cells, three times for each sample. The experiments were repeated independently three times.

### Statistical analysis

Statistical analysis was done by GraphPad software, version 5. Data are presented as the means ± SEM or SD. Student *t* test was applied to assess the statistical significance. *P* values < 0.05 were considered significant.

## Supporting information

S1 Fig HITT is induced by DNA damage. **(A)** HITT levels were determined by real-time RT-PCR in HeLa cells with 10 nM CLA at different time periods (left) or with different concentrations of CLA for 24 h (right). **(B)** HITT levels were analyzed by real-time RT-PCR in HCT116 cells with the indicated time periods of Eto (10 μM, left) or Bleo (1 μg/ml, right) treatment. **(C)** Cell lines, p53 status, and the corresponding tissue origins were listed. **(D)** Expression of HITT was determined by real-time RT-PCR after treating additional cancer cell lines with 1 μg/ml Dox for 24 h. **(E)** Representative real-time RT-PCR bar graph showing the efficiency of HITT overexpression (up) or KD (middle) in HeLa cells. DNA damage was monitored by comet assay after DMSO or Dox treatment or at different periods of time following Dox washout. Tail moment per cell are presented in the bar graph (bottom), scale bar, 10 μm. **(F)** HR or NHEJ efficiencies of ISce-I-induced DSBs in U2OS cells containing DR-GFP (HR, left), or EJ2-GFP reporter (NHEJ, right), were determined by measuring GFP-positive cells by flow cytometry (FACS) after KD of RAD51 and XRCC4, respectively. RAD51 and XRCC4 KD efficiency were detected by WB. Data are derived from three independent experiments and presented as means ± SEM in the bar graphs (A-B and D-F). Values of controls were normalized to 1. *P < 0.05; **P < 0.01 (A, B, D, E, F); #*P* < 0.05, ##*P* < 0.01, compared with vector (“Vect.”) or si-scramble control (“Si-Ctl.”) with the same indicated treatment (E). For the raw data, see S1A and S1B Figs and S1 Fig D-F in S2 Data, S1F in S1 Raw Images. Bleo, bleomycin; CLA, calicheamicin; Dox, doxorubicin; DR, Direct Repeat; DSB, double-strand break; FACS, fluorescence-activated cell sorting; Eto, etoposide; GFP, green fluorescent protein; HITT, HIF-1α inhibitor at translation level; HR, homologous recombination; KD, knockdown; NHEJ, nonhomologous end joining; RT-PCR, reverse transcription PCR; si-, small interfering; WB, western blot; XRCC4, X-Ray Repair Cross Complementing 4.
(TIF)

S2 Fig HITT inhibits ATM activity. **(A)** Expression of HITT was determined by real-time RT-PCR after the treatments of 1 μg/ml Dox with or without 10 μM ATMi-1/2 for 24 h. **(B)** Representative images of RPA2 foci accumulation in the nuclei upon CPT treatment for 1 h or 1 h after CPT was removed with or without ATMi-2 (10 μM) treatment. **(C)** p-ATM and ATM protein levels were determined by WB in HITT stable cells with or without ATMi-1 in the presence of 1 μg/ml Dox for 24 h. **(D)** The expression levels of p-ATM, ATM, p-Chk2, and Chk2 were detected by WB in different HITT stable clones of Hela cells with 1 μg/ml Dox treatment. The expression levels of HITT in three different clones were determined by qRT-PCR. **(E)** The expression levels of p-ATM, ATM, p-Chk2, and Chk2 were detected by WB in HeLa cells transfected with CRISPR/Cas9-HITT plasmids upon treatment with 1 μg/ml Dox. **(F)** p-ATM and p-Chk2 protein levels were determined by WB in HITT KD HeLa and HCT116 cells with or without HITT recovery in the presence of 1 μg/ml Dox for 24 h. Data are derived from three independent experiments and presented as means ± SEM in the bar graphs (A, B, D, E). Values of controls were normalized to 1. *P < 0.05. For the raw data, see S2A, S2B, S2D and S2E Fig in S2 Data, S2C–S2F Fig in S1 Raw Images. ATM, Ataxia-telangiectasia mutated; ATMi-1, KU-60019; ATMi-2, KU-55933; Chk2, checkpoint kinase 2; CPT, Camptothecin; Dox, doxorubicin; KD, knockdown; HITT, HIF-1α inhibitor at translation level; N.S., no significance; qRT-PCR, quantitative reverse transcription PCR; RPA2, Replication Protein A2; Vect., vector control; WB, western blot.
(TIF)

S3 Fig HITT inhibits ATM activity. **(A)** HITT levels were analyzed by real-time RT-PCR in HeLa cells with the indicate time periods of Dox (1 μg/ml) treatment. **(B)** The expression levels of the indicated proteins were detected by WB after HITT overexpression in H1299 and SW620 treated with the indicated concentrations of Dox for 24 h. **(C, D)** The expression levels of the indicated proteins were detected by WB after HITT overexpression or KD in HeLa (C) and HCT116 (D) cells treated with 1 μg/ml Bleo for 24 h. (**E)** The expression levels of the indicated proteins were detected by WB after HITT overexpression or KD in HeLa cells treated with 10 μM Eto for 24 h. **(F)** HITT levels were analyzed by real-time RT-PCR in a different cell-cycle phase of HeLa cells after TdR double-block method induced synchrony. The cell-cycle distribution was determined by PI staining combined with flow cytometer analysis. **(G)** Cell proliferation was measured by BrdU incorporation assay in the Vect. and HITT stable HeLa cells. Representative images were presented (left). The average rates of BrdU positive cells were counted and presented in the bar graph (right). **(H)** Cell-cycle distribution was analyzed by PI staining in the Vect. and HITT stable HeLa lines with the indicated treatments for 24 h. Data are derived from three independent experiments and presented as means ± SEM in the bar graphs (A, F-H). Values of controls were normalized to 1. *P < 0.05. For the raw data, see S3A Fig and S3F–S3H Fig in S2 Data, S3B–S3E Fig in S1 Raw Images. ATM, Ataxia-telangiectasia mutated; Bleo, bleomycin; BrdU, bromodeoxyuridine; Dox, doxorubicin; Eto, etoposide; KD, knockdown; HITT, HIF-1α inhibitor at translation level; N.S., no significance; PI, propidium iodide; RT-PCR, reverse transcription PCR; Vect., vector control; WB, western blot.
(TIF)

S4 Fig HITT inhibits ATM recruitment to the chromatin upon DSBs. **(A)** Expression of HITT was analyzed by real-time RT-PCR after treating cells with Dox (1 μg/ml) and/or KD NBS1 for 24 h. **(B)** The expression levels and patterns of RAD50 and p-ATM were determined by immunofluorescence staining after treatment of Dox in HITT stable HeLa cells. Representative images are presented (left). The average rates of RAD50 or p-ATM nuclear foci-positive cells were counted and are presented in the bar graph (right). **(C)** Chromatin-associated RAD50, NBS1, ATM, and ATR were determined by chromatin-fraction assay in two independent stable HITT HeLa sublines (left) or in HITT KD cells (right) in the presence of Dox (1 μg/ml, 24 h). Data are derived from three independent experiments and presented as means ± SEM in the bar graph; **P < 0.01; ##*P* < 0.01; relative to Dox-treated control (B). For the raw data, see S4A and S4B Fig in S2 Data, S4C in S1 Raw Images. ATM, Ataxia-telangiectasia mutated; ATR, Ataxia Telangiectasia And Rad3-Related Protein; Dox, doxorubicin; DSB, double-strand break; KD, knockdown; HITT, HIF-1α inhibitor at translation level; NBS1, Nijmegen Breakage Syndrome 1; N.S., no significance; RT-PCR, reverse transcription PCR.
(TIF)

S5 Fig EGR1 regulates HITT expression. **(A)** HITT levels were determined by real-time RT-PCR in HCT116 and HeLa cells after treatment of different time periods of Dox (1 μg/ml) in the presence or absence of RNA synthesis inhibitor ActD. **(B)** The relative binding strength between the indicated transcription factors and HITT promoter region were obtained by UCSC ChIP sequence data. **(C)** The expression levels of TAF1 were determined by WB assay in cancer cell lines after exposure to Dox (1 μg/ml) (left) or Bleo (1 μg/ml) (right) for 24 h. **(D, E)** HITT levels and pGL3-HITT-promoter-luc activity were analyzed after transfection with different concentrations of EGR1 (D) expressing plasmids or siRNA-mediated EGR1 KD (E) in both HCT116 and HeLa cells. The efficiency of EGR1 overexpression or KD were determined by WB (bottom). **(F)** The expression level of HITT was determined by real-time RT-PCR in the control or HITT stable HeLa cell line after treatment with 1 μg/ml Dox for 24 h. EGR1 KD efficiency was confirmed by WB (right). Data are derived from three independent experiments and presented as means ± SEM. Values of controls were normalized to 1 (A, D-F). *P < 0.05; **P < 0.01; #*P* < 0.05, compared with Dox-treated control cells; $ $, *P* < 0.01, compared with Dox-treated HITT overexpression cells. For the raw data, see S5A and S5B Fig and S5D–S5F Fig in S2 Data, S5C–S5F Fig in S1 Raw Images. ActD, actinomycin D; ATM, Ataxia-telangiectasia mutated; Bleo, bleomycin; ChIP, chromatin immunoprecipitation; Dox, doxorubicin; EGR1, Early Growth Response 1; HITT, HIF-1α inhibitor at translation level; KD, knockdown; siRNA, small interfering RNA; TAF1, TATA-box binding protein associated factor 1; WB, western blot.
(TIF)

S6 Fig HITT is down-regulated and may contribute to ATM activation in human colon cancers. **(A)** HITT levels in 40 human colon cancers (“T”) and paired adjacent normal controls (“N”). **(B-E)** Representative WB (B) and quantification (40 pairs, C-E) of p-ATM, ATM, p-ATR, ATR, and NBS1 in human colon cancers (“T”) and paired adjacent normal controls (“N”). **(F)** Samples were divided into “NBS1 low” and “NBS1 high” groups according to the median of NBS1 (T/N). The fold changes of p-ATM (T/N) in the two groups is shown on the y-axis. **(G)** Samples were divided into “HITT low,” “HITT middle,” and “HITT high” groups according to the median of HITT (T/N). The fold changes of p-ATM (T/N) in the three groups is shown on the y-axis. **(H-I)** Quantification (40 pairs) of p-ATR, (29 pairs) of ATR, in human colon cancers (T) and paired adjacent normal controls (N). **(J)** Samples were divided into “HITT low,” “HITT middle,” and “HITT high” groups according to the median of HITT (T/N). The fold changes of p-ATR (T/N) in the three groups are shown on the y-axis. *P < 0.05; **P < 0.01 (A, C, G, and H). For the raw data, see S6A Fig and S6C–S6J Fig in S2 Data, S6B in S1 Raw Images. ATM, Ataxia-telangiectasia mutated; ATR, Ataxia Telangiectasia And Rad3-Related Protein; HITT, HIF-1α inhibitor at translation level; NBS1, Nijmegen Breakage Syndrome 1; N.S., not significant; WB, western blot.
(TIF)

S7 Fig HITT sensitizes cells’ response to genotoxic treatment in an ATM activity–dependent manner. **(A)** The survival rates of cells with the indicated after overexpression different fragments HITT were determined by MTT assay (left). The expression level of FL and fragments were detected by RT-PCR (right). **(B, C)** The survival rates were evaluated by MTT assay in HeLa (B) and HCT116 (C) cells treated with indicated concentrations of Bleo for 24 h. **(D, E)** The survival rates was evaluated by MTT assay in HeLa cells after HITT overexpression (D) or knockdown (E) after the treatment of the indicated concentrations of Eto and/or 10 μM ATMi-2 for 24 h. **(F, G)** The survival rates were evaluated by MTT assay in H1299 cells after HITT overexpression (F) or knockdown (G) after the treatment of the indicated concentrations of Dox and/or 10 μM ATMi-1 for 24 h. Data are derived from three independent experiments and presented as means ± SEM. Values of controls were normalized to 1 (A-G). *P < 0.05; **P < 0.01 (A-G). For the raw data, see S7A–S7G Fig in S2 Data. ATM, Ataxia-telangiectasia mutated; ATMi-2, KU-55933; Bleo, bleomycin; Dox, doxorubicin; Eto, etoposide; HITT, HIF-1α inhibitor at translation level; MTT, 3-(4,5-dimethylthiazol-2-yl)-2,5-diphenyltetrazolium bromide; N.S., no significance; RT-PCR, reverse transcription PCR; Vect., vector control.
(TIF)

S1 Data Numerical values underlying main figures. From Figs 1A–1G, 2A, 2D, 2E, 3B, 3E–3G, 3I, 3J, 4A, 4C–4E, 5A–5G and 6B–6E.
(XLSX)

S2 Data Numerical values underlying supporting figures. From S1A and S1B Fig, S1D–S1F Fig, S2A, S2B, S2D, S2E and S3A Fig, S3F–S3H Fig, S4A, S4B, S5A and S5B Fig, S5D–S5F Fig, S6A Fig, S6C–S6J Fig, and S7A–S7G Fig.
(XLSX)

S1 Raw Images Raw images underlying figures. From Figs 2B–2E, 3A, 3C, 3D, 3H, 3K, 3L, 4B–4D, 4F and 6F and S1F, S2C–S2F Fig, S3B–S3E Fig and S4C, S5C–S5F Fig and S6B Fig.
(PDF)

## Abbreviations

A-T: ataxia-telangiectasia
ATM: Ataxia-telangiectasia mutated
ATMi: ATM inhibitor
ATMi-1: KU-60019
ATMi-2: KU-55933
ATR: Ataxia Telangiectasia And Rad3-Related Protein
BIN1: Bridging Integrator 1
Bleo: bleomycin
BRCA1: Breast Cancer gene 1
BrdU: bromodeoxyuridine
ChIP: chromatin immunoprecipitation
ChIP-seq: ChIP sequencing
Chk2: checkpoint kinase 2
CHX: cycloheximide
CLA: calicheamicin
CLIP: UV cross-linking and immunoprecipitation
DDR: DNA damage response
DINO: Damage Induced Noncoding
Dox: doxorubicin
DR: Direct Repeat
DSB: double-strand break
EGR1: Early Growth Response 1
Eto: etoposide
GFP: green fluorescent protein
HIF-1α: hypoxia-inducible factor 1α
HITT: HIF-1α inhibitor at translation level
HR: homologous recombination
lncRNA: long noncoding RNA
IgG: immunoglobulin G
IP: immunoprecipitation
IR: ionizing radiation
KD: knockdown
Mre11: Meiotic Recombination 11
MRN: MRE11-RAD50-NBS1
MT: mutant type
MTT: 3-(4,5-dimethylthiazol-2-yl)-2,5-diphenyltetrazolium bromide
NBS1: Nijmegen Breakage Syndrome 1
NHEJ: nonhomologous end joining
N.S.: not significant
nt: nucleotides
PANDA: P21 associated ncRNA DNA damage activated
PARP: poly ADP-ribose polymerase
PTEN: phosphate and tension homology deleted on chromosome ten
siRNA: small interfering RNA
TAF1: TATA-Box Binding Protein Associated Factor 1
TNF-α: tumor necrosis factor-α
WB: western blot
XRCC4: X-Ray Repair Cross Complementing 4.
