# Supplementary figures and images for: A long noncoding RNA sensitizes genotoxic treatment by attenuating ATM activation and homologous recombination repair in cancers
Source: PLoS Biol. 2020 Mar 23;18(3):e3000666. doi: 10.1371/journal.pbio.3000666 (PMC7138317; doi:10.1371/journal.pbio.3000666)

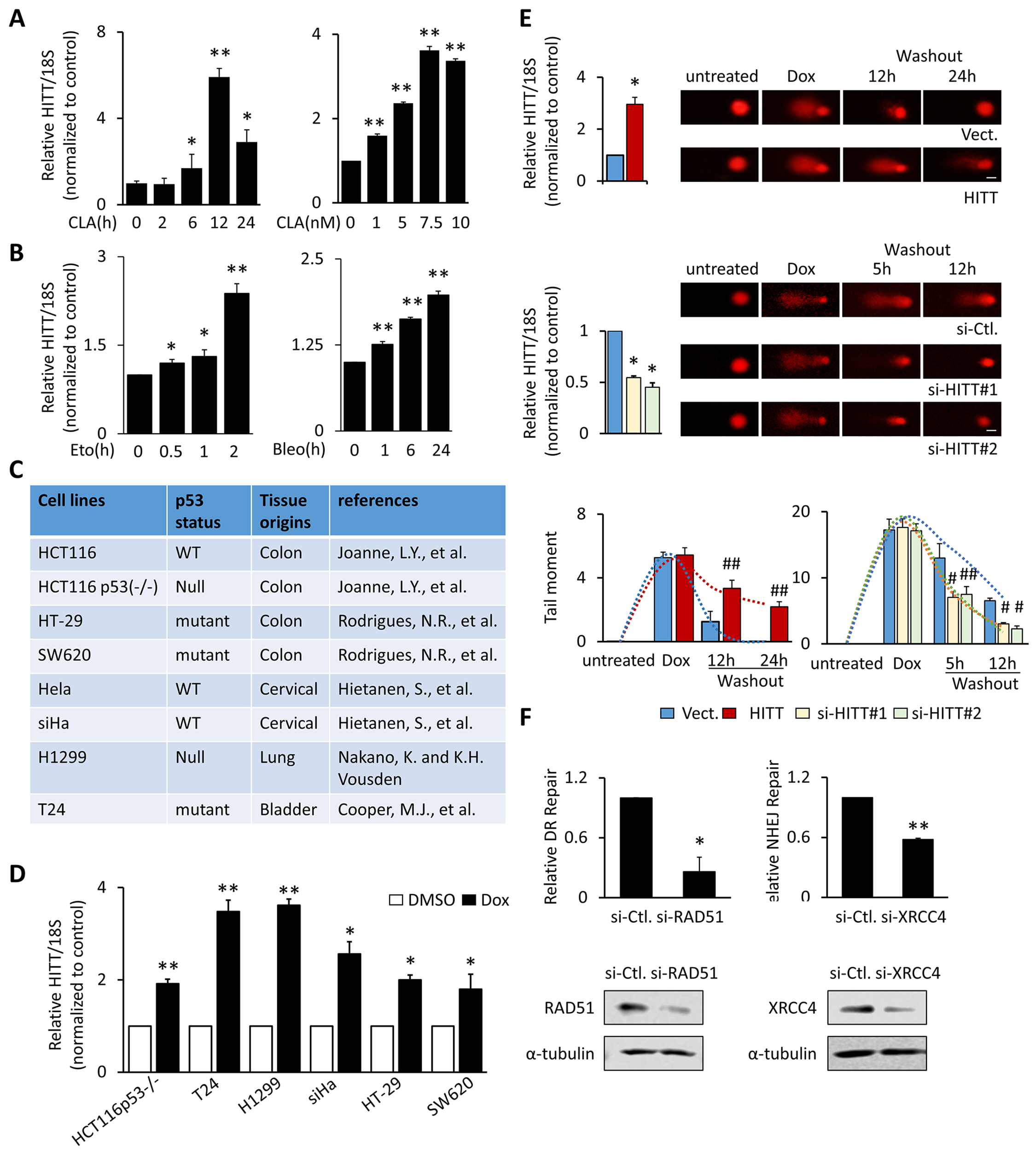

Supplement: S1 Fig — (A) HITT levels were determined by real-time RT-PCR in HeLa cells with 10 nM CLA at different time periods (left) or with different concentrations of CLA for 24 h (right). (B) HITT levels were analyzed by real-time RT-PCR in HCT116 cells with the indicated time periods of Eto (10 μM, left) or Bleo (1 μg/ml, right) treatment. (C) Cell lines, p53 status, and the corresponding tissue origins were listed. (D) Expression of HITT was determined by real-time RT-PCR after treating additional cancer cell lines with 1 μg/ml Dox for 24 h. (E) Representative real-time RT-PCR bar graph showing the efficiency of HITT overexpression (up) or KD (middle) in HeLa cells. DNA damage was monitored by comet assay after DMSO or Dox treatment or at different periods of time following Dox washout. Tail moment per cell are presented in the bar graph (bottom), scale bar, 10 μm. (F) HR or NHEJ efficiencies of ISce-I-induced DSBs in U2OS cells containing DR-GFP (HR, left), or EJ2-GFP reporter (NHEJ, right), were determined by measuring GFP-positive cells by flow cytometry (FACS) after KD of RAD51 and XRCC4, respectively. RAD51 and XRCC4 KD efficiency were detected by WB. Data are derived from three independent experiments and presented as means ± SEM in the bar graphs (A-B and D-F). Values of controls were normalized to 1. *P < 0.05; **P < 0.01 (A, B, D, E, F); #P < 0.05, ##P < 0.01, compared with vector (“Vect.”) or si-scramble control (“Si-Ctl.”) with the same indicated treatment (E). For the raw data, see S1A and S1B Figs and S1 Fig D-F in S2 Data, S1F in S1 Raw Images. Bleo, bleomycin; CLA, calicheamicin; Dox, doxorubicin; DR, Direct Repeat; DSB, double-strand break; FACS, fluorescence-activated cell sorting; Eto, etoposide; GFP, green fluorescent protein; HITT, HIF-1α inhibitor at translation level; HR, homologous recombination; KD, knockdown; NHEJ, nonhomologous end joining; RT-PCR, reverse transcription PCR; si-, small interfering; WB, western blot; XRCC4, X-Ray Repair Cross Complementin [file pbio.3000666.s001.TIF]

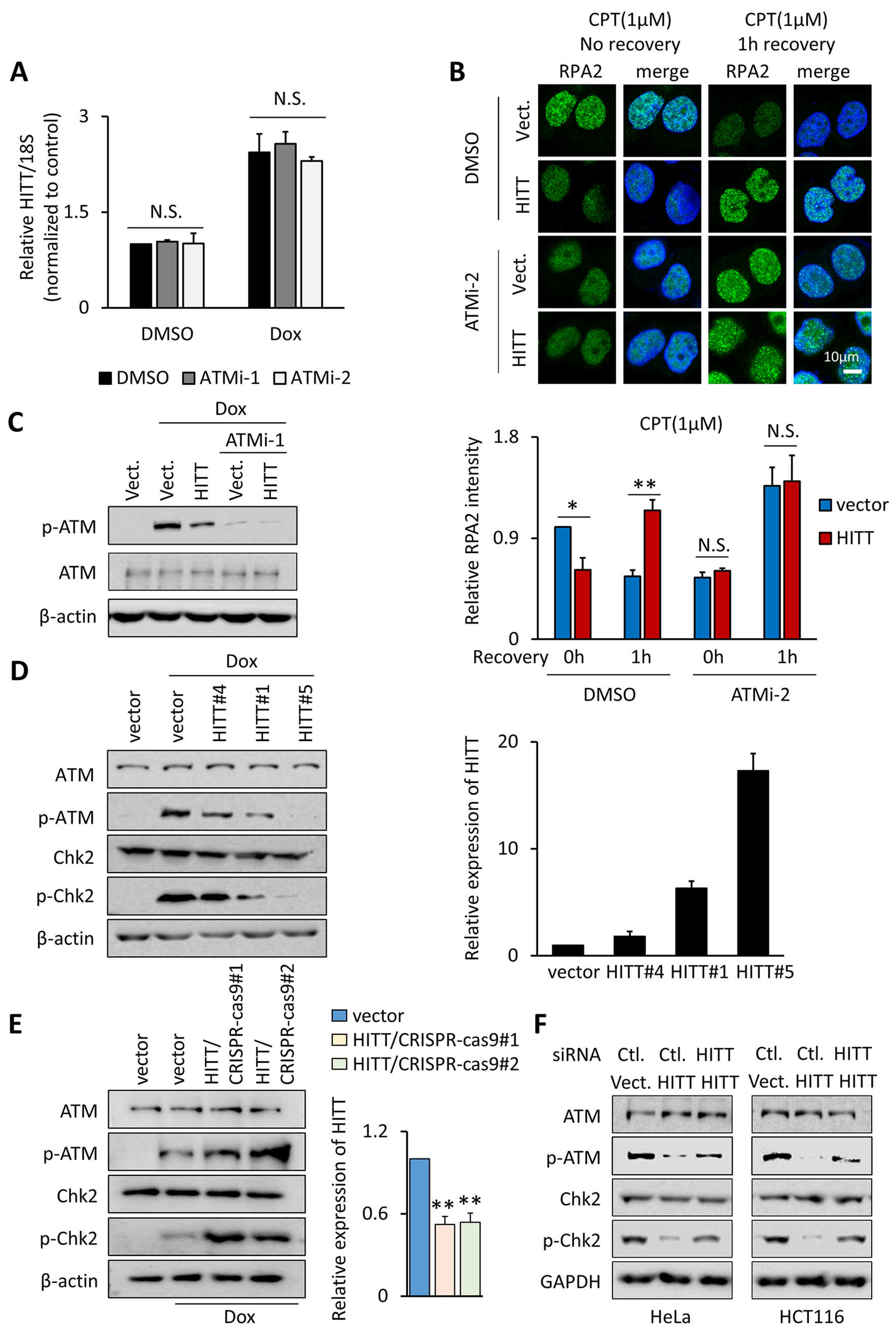

Supplement: S2 Fig — (A) Expression of HITT was determined by real-time RT-PCR after the treatments of 1 μg/ml Dox with or without 10 μM ATMi-1/2 for 24 h. (B) Representative images of RPA2 foci accumulation in the nuclei upon CPT treatment for 1 h or 1 h after CPT was removed with or without ATMi-2 (10 μM) treatment. (C) p-ATM and ATM protein levels were determined by WB in HITT stable cells with or without ATMi-1 in the presence of 1 μg/ml Dox for 24 h. (D) The expression levels of p-ATM, ATM, p-Chk2, and Chk2 were detected by WB in different HITT stable clones of Hela cells with 1 μg/ml Dox treatment. The expression levels of HITT in three different clones were determined by qRT-PCR. (E) The expression levels of p-ATM, ATM, p-Chk2, and Chk2 were detected by WB in HeLa cells transfected with CRISPR/Cas9-HITT plasmids upon treatment with 1 μg/ml Dox. (F) p-ATM and p-Chk2 protein levels were determined by WB in HITT KD HeLa and HCT116 cells with or without HITT recovery in the presence of 1 μg/ml Dox for 24 h. Data are derived from three independent experiments and presented as means ± SEM in the bar graphs (A, B, D, E). Values of controls were normalized to 1. *P < 0.05. For the raw data, see S2A, S2B, S2D and S2E Fig in S2 Data, S2C–S2F Fig in S1 Raw Images. ATM, Ataxia-telangiectasia mutated; ATMi-1, KU-60019; ATMi-2, KU-55933; Chk2, checkpoint kinase 2; CPT, Camptothecin; Dox, doxorubicin; KD, knockdown; HITT, HIF-1α inhibitor at translation level; N.S., no significance; qRT-PCR, quantitative reverse transcription PCR; RPA2, Replication Protein A2; Vect., vector control; WB, western blot. (TIF) [file pbio.3000666.s002.TIF]

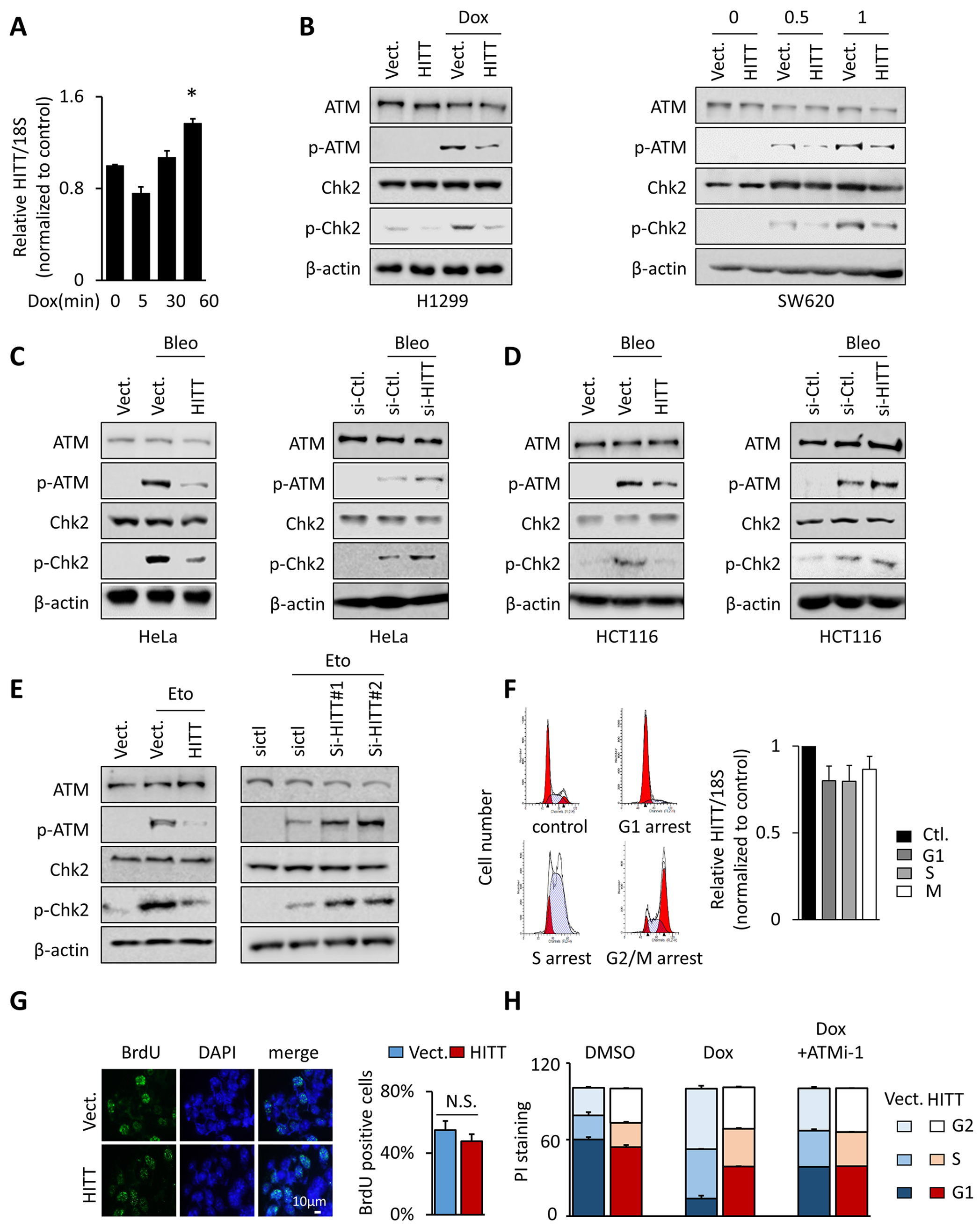

Supplement: S3 Fig — (A) HITT levels were analyzed by real-time RT-PCR in HeLa cells with the indicate time periods of Dox (1 μg/ml) treatment. (B) The expression levels of the indicated proteins were detected by WB after HITT overexpression in H1299 and SW620 treated with the indicated concentrations of Dox for 24 h. (C, D) The expression levels of the indicated proteins were detected by WB after HITT overexpression or KD in HeLa (C) and HCT116 (D) cells treated with 1 μg/ml Bleo for 24 h. (E) The expression levels of the indicated proteins were detected by WB after HITT overexpression or KD in HeLa cells treated with 10 μM Eto for 24 h. (F) HITT levels were analyzed by real-time RT-PCR in a different cell-cycle phase of HeLa cells after TdR double-block method induced synchrony. The cell-cycle distribution was determined by PI staining combined with flow cytometer analysis. (G) Cell proliferation was measured by BrdU incorporation assay in the Vect. and HITT stable HeLa cells. Representative images were presented (left). The average rates of BrdU positive cells were counted and presented in the bar graph (right). (H) Cell-cycle distribution was analyzed by PI staining in the Vect. and HITT stable HeLa lines with the indicated treatments for 24 h. Data are derived from three independent experiments and presented as means ± SEM in the bar graphs (A, F-H). Values of controls were normalized to 1. *P < 0.05. For the raw data, see S3A Fig and S3F–S3H Fig in S2 Data, S3B–S3E Fig in S1 Raw Images. ATM, Ataxia-telangiectasia mutated; Bleo, bleomycin; BrdU, bromodeoxyuridine; Dox, doxorubicin; Eto, etoposide; KD, knockdown; HITT, HIF-1α inhibitor at translation level; N.S., no significance; PI, propidium iodide; RT-PCR, reverse transcription PCR; Vect., vector control; WB, western blot. (TIF) [file pbio.3000666.s003.TIF]

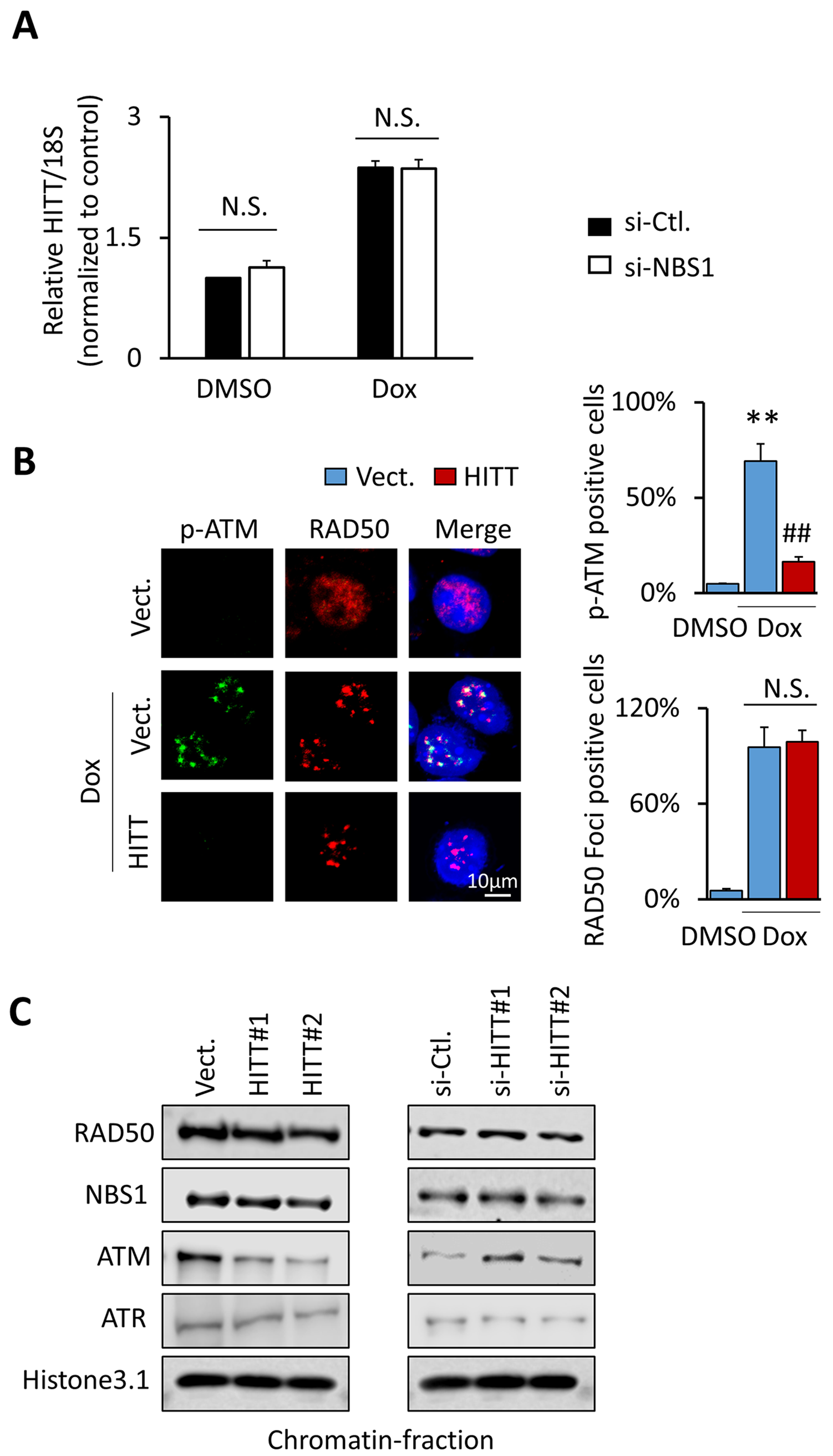

Supplement: S4 Fig — (A) Expression of HITT was analyzed by real-time RT-PCR after treating cells with Dox (1 μg/ml) and/or KD NBS1 for 24 h. (B) The expression levels and patterns of RAD50 and p-ATM were determined by immunofluorescence staining after treatment of Dox in HITT stable HeLa cells. Representative images are presented (left). The average rates of RAD50 or p-ATM nuclear foci-positive cells were counted and are presented in the bar graph (right). (C) Chromatin-associated RAD50, NBS1, ATM, and ATR were determined by chromatin-fraction assay in two independent stable HITT HeLa sublines (left) or in HITT KD cells (right) in the presence of Dox (1 μg/ml, 24 h). Data are derived from three independent experiments and presented as means ± SEM in the bar graph; **P < 0.01; ##P < 0.01; relative to Dox-treated control (B). For the raw data, see S4A and S4B Fig in S2 Data, S4C in S1 Raw Images. ATM, Ataxia-telangiectasia mutated; ATR, Ataxia Telangiectasia And Rad3-Related Protein; Dox, doxorubicin; DSB, double-strand break; KD, knockdown; HITT, HIF-1α inhibitor at translation level; NBS1, Nijmegen Breakage Syndrome 1; N.S., no significance; RT-PCR, reverse transcription PCR. (TIF) [file pbio.3000666.s004.TIF]

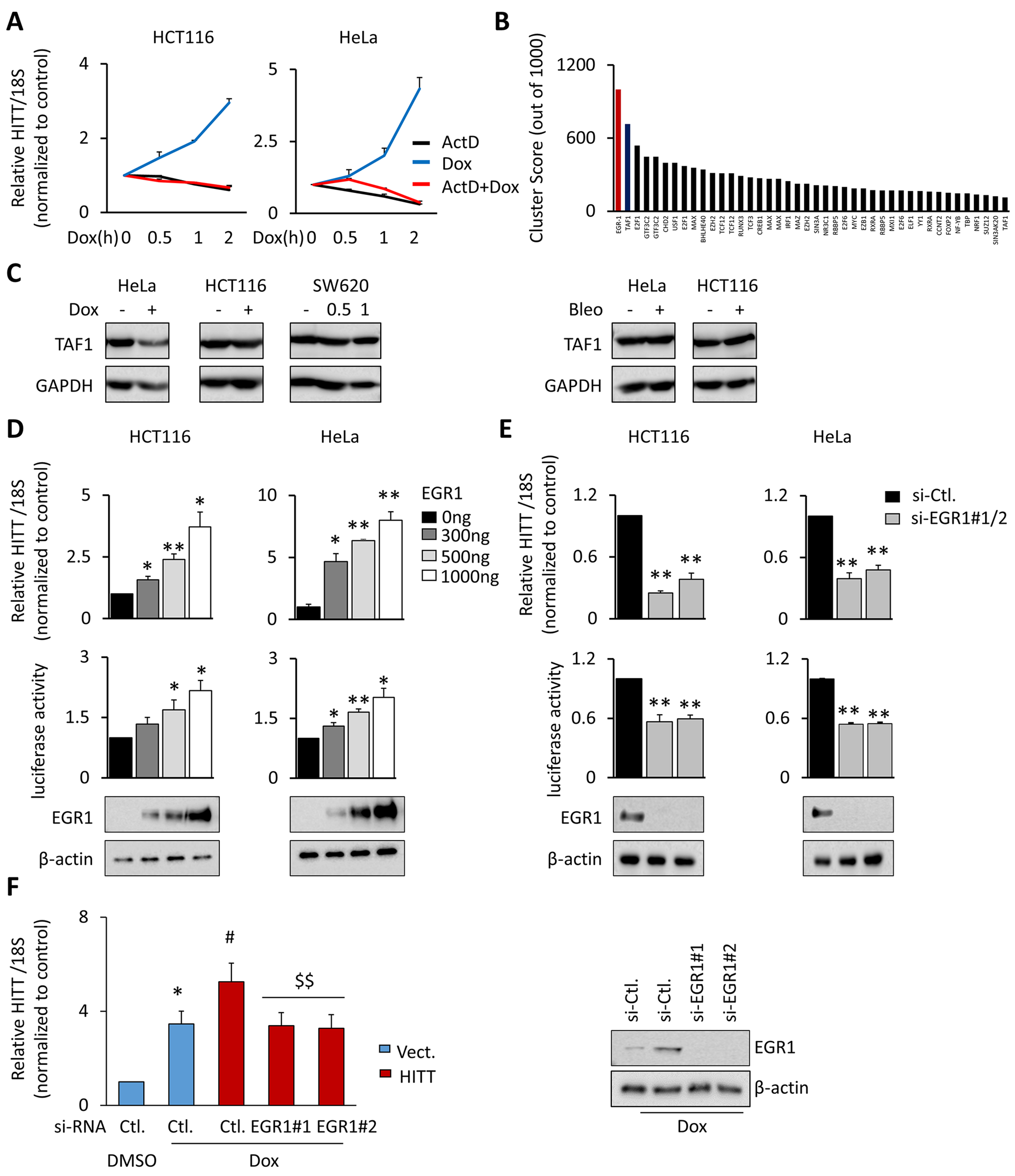

Supplement: S5 Fig — (A) HITT levels were determined by real-time RT-PCR in HCT116 and HeLa cells after treatment of different time periods of Dox (1 μg/ml) in the presence or absence of RNA synthesis inhibitor ActD. (B) The relative binding strength between the indicated transcription factors and HITT promoter region were obtained by UCSC ChIP sequence data. (C) The expression levels of TAF1 were determined by WB assay in cancer cell lines after exposure to Dox (1 μg/ml) (left) or Bleo (1 μg/ml) (right) for 24 h. (D, E) HITT levels and pGL3-HITT-promoter-luc activity were analyzed after transfection with different concentrations of EGR1 (D) expressing plasmids or siRNA-mediated EGR1 KD (E) in both HCT116 and HeLa cells. The efficiency of EGR1 overexpression or KD were determined by WB (bottom). (F) The expression level of HITT was determined by real-time RT-PCR in the control or HITT stable HeLa cell line after treatment with 1 μg/ml Dox for 24 h. EGR1 KD efficiency was confirmed by WB (right). Data are derived from three independent experiments and presented as means ± SEM. Values of controls were normalized to 1 (A, D-F). *P < 0.05; **P < 0.01; #P < 0.05, compared with Dox-treated control cells; $ $, P < 0.01, compared with Dox-treated HITT overexpression cells. For the raw data, see S5A and S5B Fig and S5D–S5F Fig in S2 Data, S5C–S5F Fig in S1 Raw Images. ActD, actinomycin D; ATM, Ataxia-telangiectasia mutated; Bleo, bleomycin; ChIP, chromatin immunoprecipitation; Dox, doxorubicin; EGR1, Early Growth Response 1; HITT, HIF-1α inhibitor at translation level; KD, knockdown; siRNA, small interfering RNA; TAF1, TATA-box binding protein associated factor 1; WB, western blot. (TIF) [file pbio.3000666.s005.TIF]

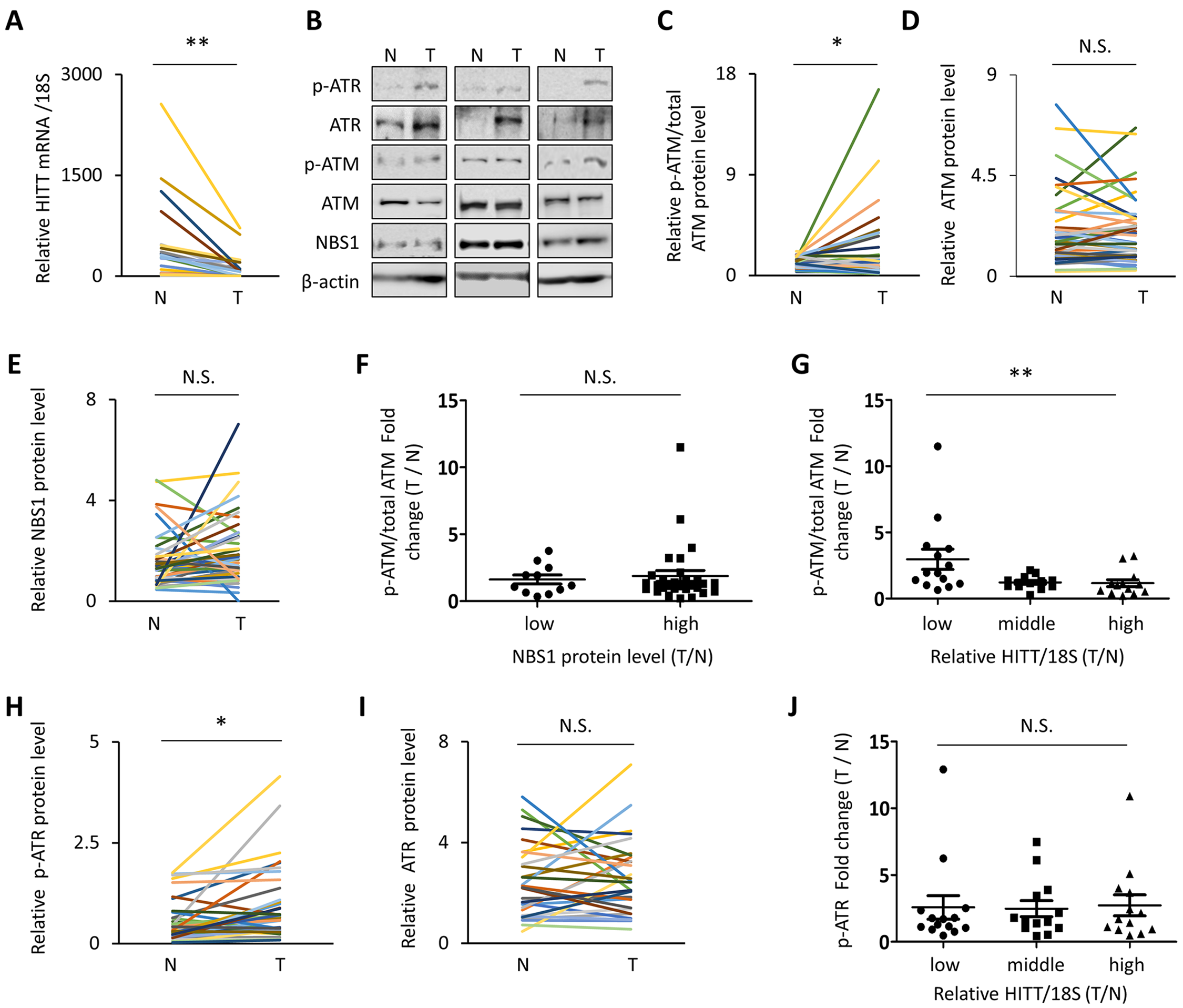

Supplement: S6 Fig — (A) HITT levels in 40 human colon cancers (“T”) and paired adjacent normal controls (“N”). (B-E) Representative WB (B) and quantification (40 pairs, C-E) of p-ATM, ATM, p-ATR, ATR, and NBS1 in human colon cancers (“T”) and paired adjacent normal controls (“N”). (F) Samples were divided into “NBS1 low” and “NBS1 high” groups according to the median of NBS1 (T/N). The fold changes of p-ATM (T/N) in the two groups is shown on the y-axis. (G) Samples were divided into “HITT low,” “HITT middle,” and “HITT high” groups according to the median of HITT (T/N). The fold changes of p-ATM (T/N) in the three groups is shown on the y-axis. (H-I) Quantification (40 pairs) of p-ATR, (29 pairs) of ATR, in human colon cancers (T) and paired adjacent normal controls (N). (J) Samples were divided into “HITT low,” “HITT middle,” and “HITT high” groups according to the median of HITT (T/N). The fold changes of p-ATR (T/N) in the three groups are shown on the y-axis. *P < 0.05; **P < 0.01 (A, C, G, and H). For the raw data, see S6A Fig and S6C–S6J Fig in S2 Data, S6B in S1 Raw Images. ATM, Ataxia-telangiectasia mutated; ATR, Ataxia Telangiectasia And Rad3-Related Protein; HITT, HIF-1α inhibitor at translation level; NBS1, Nijmegen Breakage Syndrome 1; N.S., not significant; WB, western blot. (TIF) [file pbio.3000666.s006.TIF]

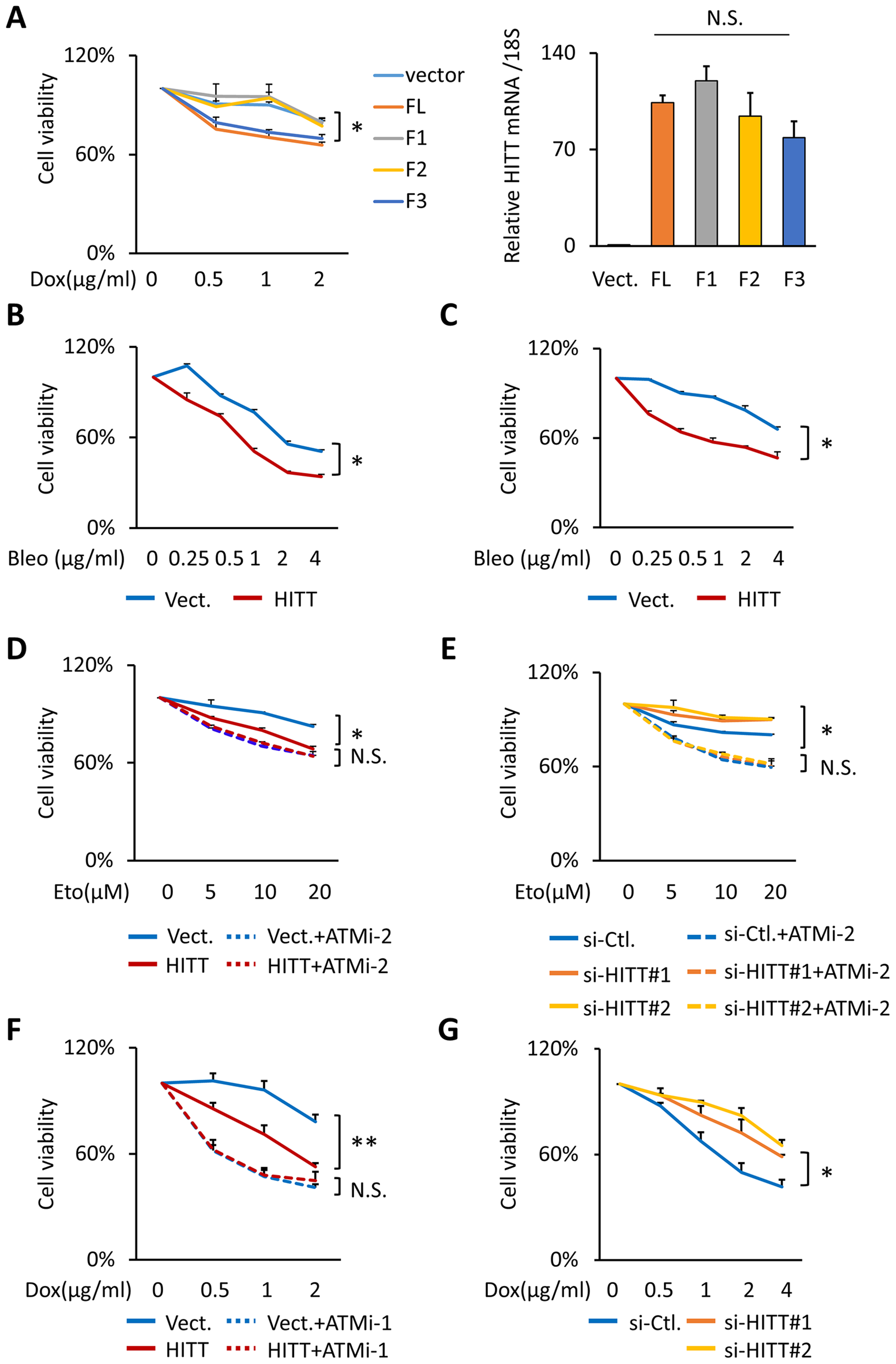

Supplement: S7 Fig — (A) The survival rates of cells with the indicated after overexpression different fragments HITT were determined by MTT assay (left). The expression level of FL and fragments were detected by RT-PCR (right). (B, C) The survival rates were evaluated by MTT assay in HeLa (B) and HCT116 (C) cells treated with indicated concentrations of Bleo for 24 h. (D, E) The survival rates was evaluated by MTT assay in HeLa cells after HITT overexpression (D) or knockdown (E) after the treatment of the indicated concentrations of Eto and/or 10 μM ATMi-2 for 24 h. (F, G) The survival rates were evaluated by MTT assay in H1299 cells after HITT overexpression (F) or knockdown (G) after the treatment of the indicated concentrations of Dox and/or 10 μM ATMi-1 for 24 h. Data are derived from three independent experiments and presented as means ± SEM. Values of controls were normalized to 1 (A-G). *P < 0.05; **P < 0.01 (A-G). For the raw data, see S7A–S7G Fig in S2 Data. ATM, Ataxia-telangiectasia mutated; ATMi-2, KU-55933; Bleo, bleomycin; Dox, doxorubicin; Eto, etoposide; HITT, HIF-1α inhibitor at translation level; MTT, 3-(4,5-dimethylthiazol-2-yl)-2,5-diphenyltetrazolium bromide; N.S., no significance; RT-PCR, reverse transcription PCR; Vect., vector control. (TIF) [file pbio.3000666.s007.TIF]
