# Supplementary material for: A long noncoding RNA sensitizes genotoxic treatment by attenuating ATM activation and homologous recombination repair in cancers
Source: PLoS Biol. 2020 Mar 23;18(3):e3000666. doi: 10.1371/journal.pbio.3000666 (PMC7138317; doi:10.1371/journal.pbio.3000666)
Supplement: S1 Raw Images — From Figs 2B–2E, 3A, 3C, 3D, 3H, 3K, 3L, 4B–4D, 4F and 6F and S1F, S2C–S2F Fig, S3B–S3E Fig and S4C, S5C–S5F Fig and S6B Fig. (PDF) [file pbio.3000666.s010.pdf]

Fig.2B

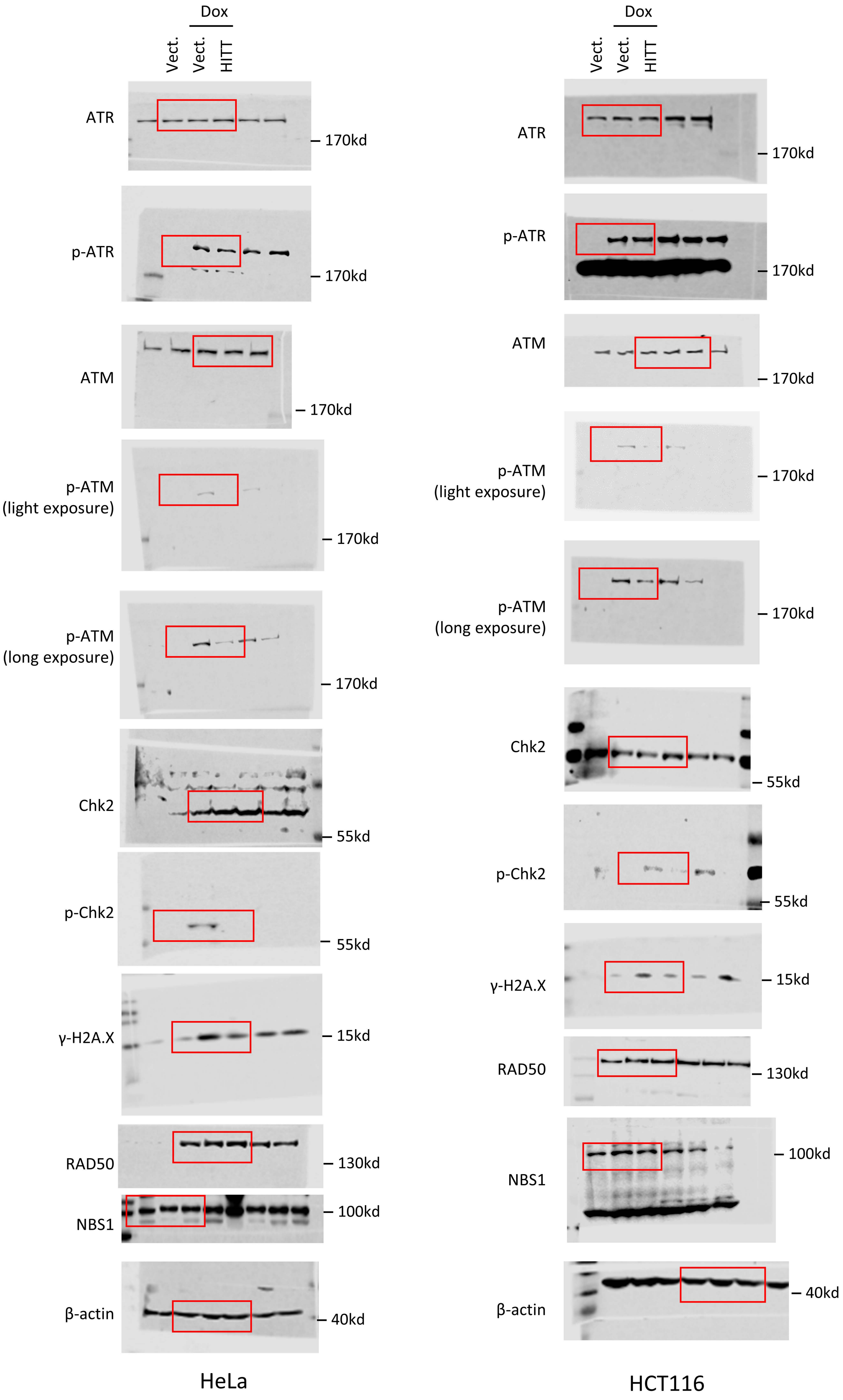
Dox
Vect.
Vect.
HITT
ATR
170kd
p-ATR
170kd
ATM
170kd
p-ATM
(light exposure)
170kd
p-ATM
(long exposure)
170kd
Chk2
55kd
p-Chk2
55kd
γ-H2A.X
15kd
RAD50
130kd
NBS1
100kd
β-actin
40kd
HeLa
HCT116

Fig.2C

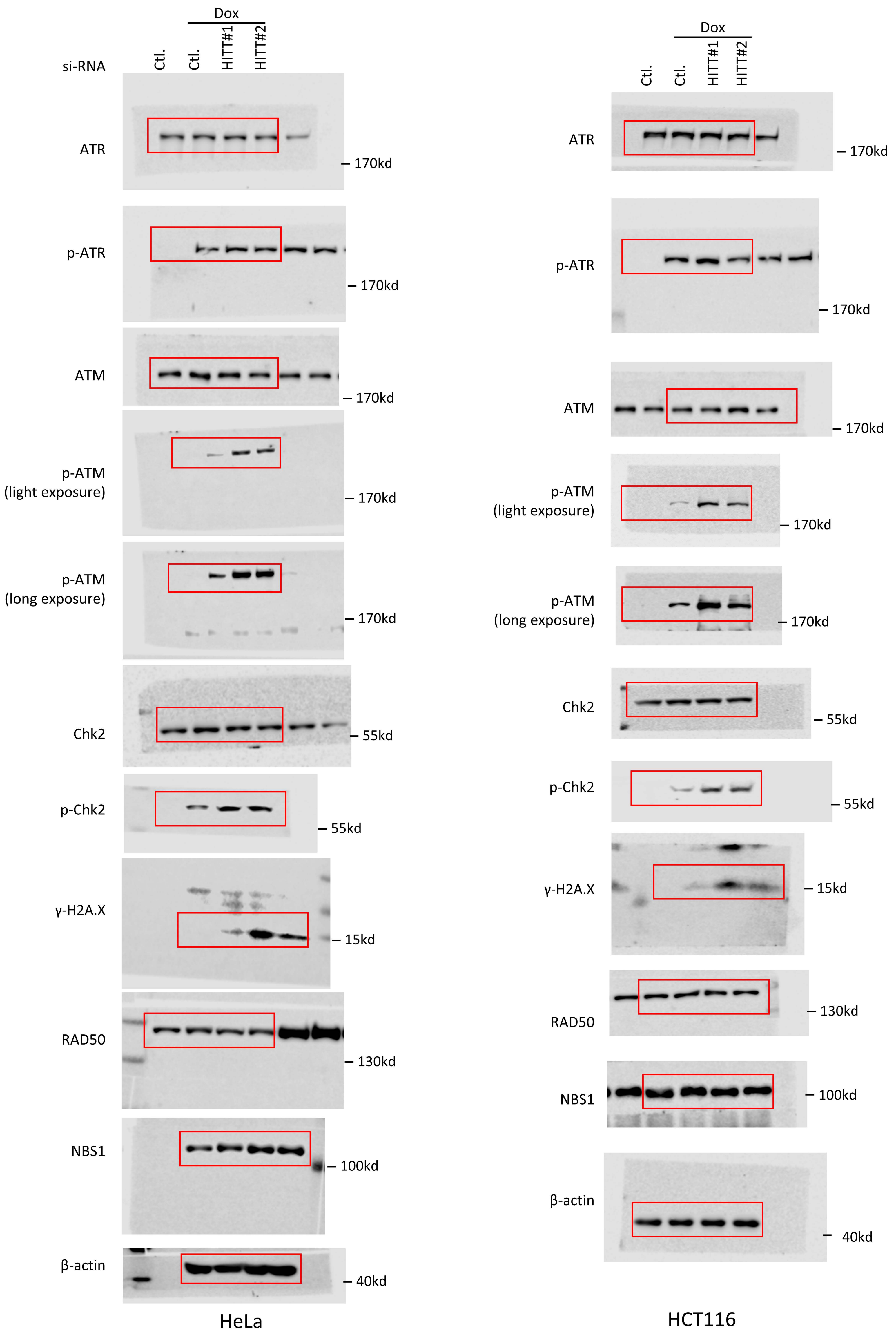

Dox
si-RNA
Ctl.
Ctl.
HITT#1
HITT#2
ATR
170kd
p-ATR
170kd
ATM
170kd
p-ATM
(light exposure)
170kd
p-ATM
(long exposure)
170kd
Chk2
55kd
p-Chk2
55kd
γ-H2A.X
15kd
RAD50
130kd
NBS1
100kd
β-actin
40kd
HeLa
Dox
Ctl.
Ctl.
HITT#1
HITT#2
ATR
170kd
p-ATR
170kd
ATM
170kd
p-ATM
(light exposure)
170kd
p-ATM
(long exposure)
170kd
Chk2
55kd
p-Chk2
55kd
γ-H2A.X
15kd
RAD50
130kd
NBS1
100kd
β-actin
40kd
HCT116

Fig.2D

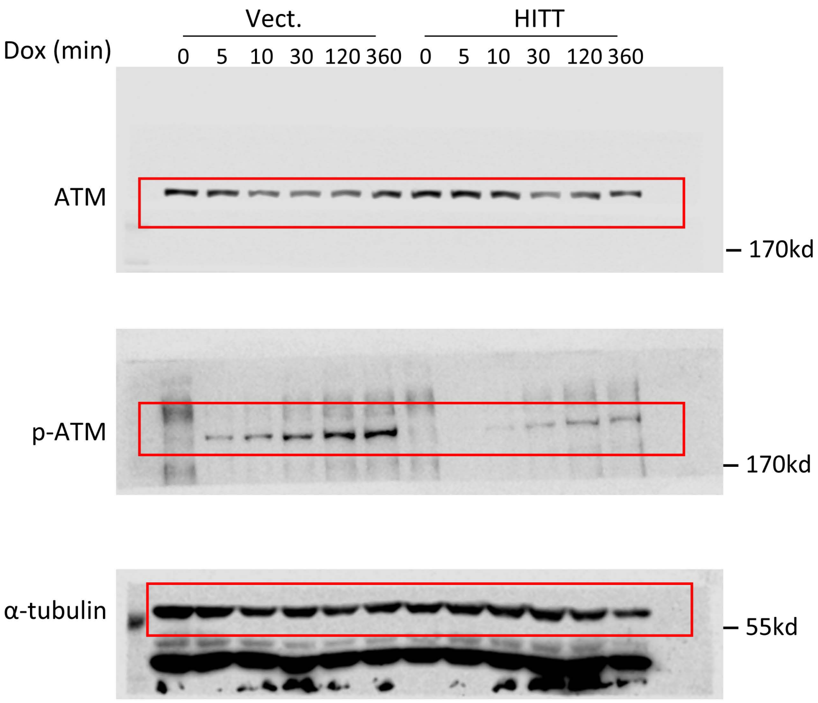
Vect.
HITT
Dox (min)
0 5 10 30 120 360 0 5 10 30 120 360
ATM
170kd
p-ATM
170kd
α-tubulin
55kd

Fig.2E

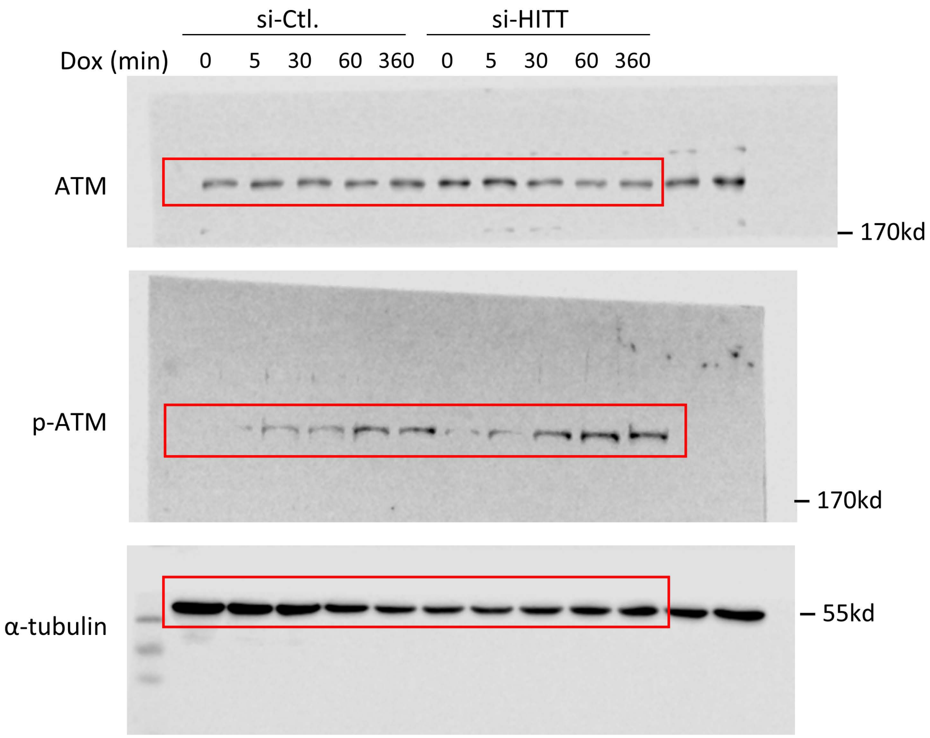

si-Ctl.
si-HITT
Dox (min)
0
5
30
60
360
0
5
30
60
360
ATM
170kd
p-ATM
170kd
α-tubulin
55kd

Fig.3A

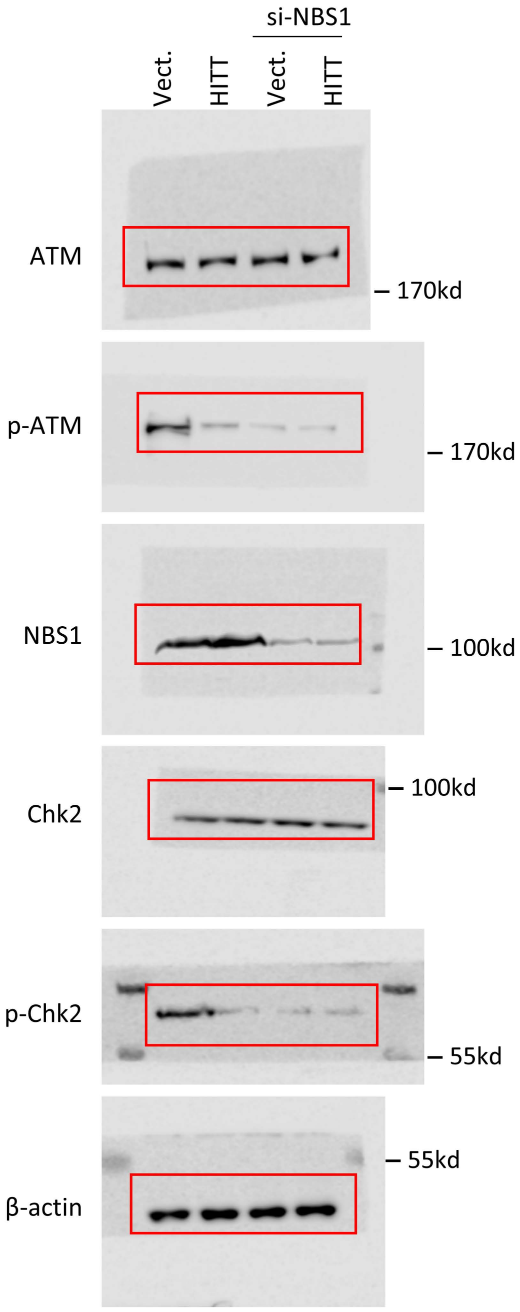
si-NBS1
Vect.
HITT
Vect.
HITT
ATM
170kd
p-ATM
170kd
NBS1
100kd
Chk2
100kd
p-Chk2
55kd
β-actin
55kd

Figure 3C

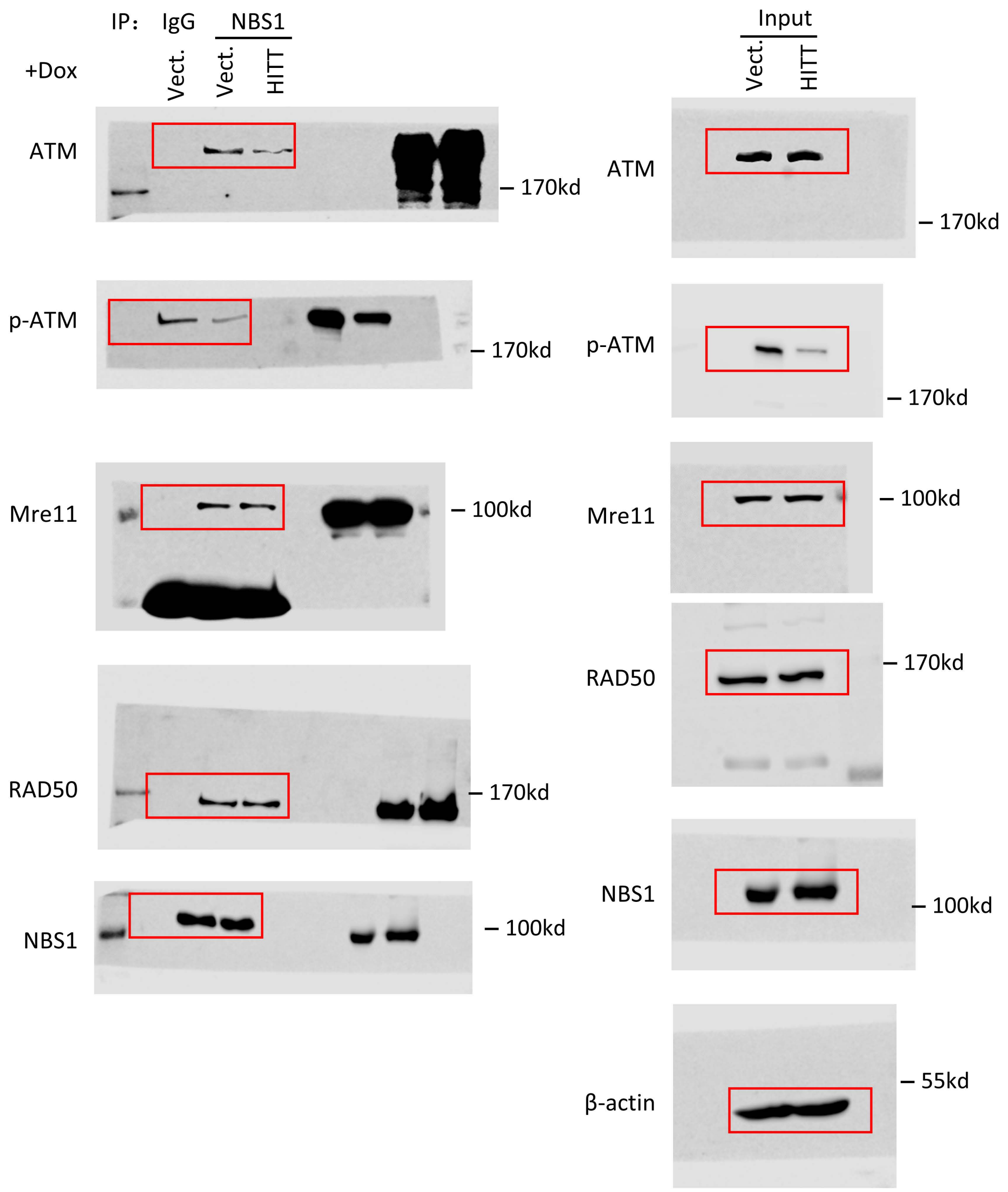

IP: IgG NBS1
+Dox
Vect.
Vect.
HITT
ATM
170kd
p-ATM
170kd
Mre11
100kd
RAD50
170kd
NBS1
100kd
Input
Vect.
HITT
ATM
170kd
p-ATM
170kd
Mre11
100kd
RAD50
170kd
NBS1
100kd
β-actin
55kd

Figure 3D

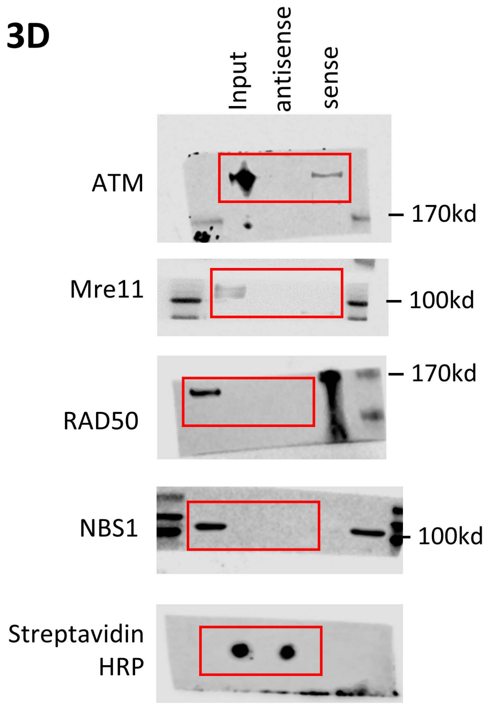
Input
antisense
sense
ATM
170kd
Mre11
100kd
170kd
RAD50
NBS1
100kd
Streptavidin
HRP

Figure 3H

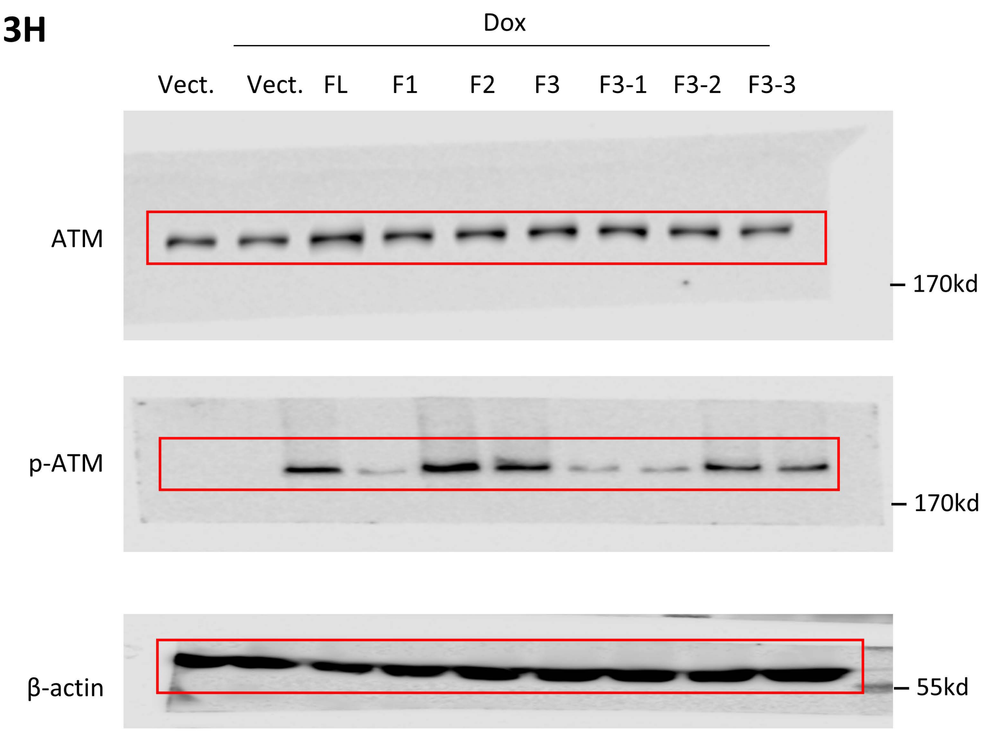
Dox
Vect.
Vect.
FL
F1
F2
F3
F3-1
F3-2
F3-3
ATM
p-ATM
β-actin
170kd
170kd
55kd

Figure 3K

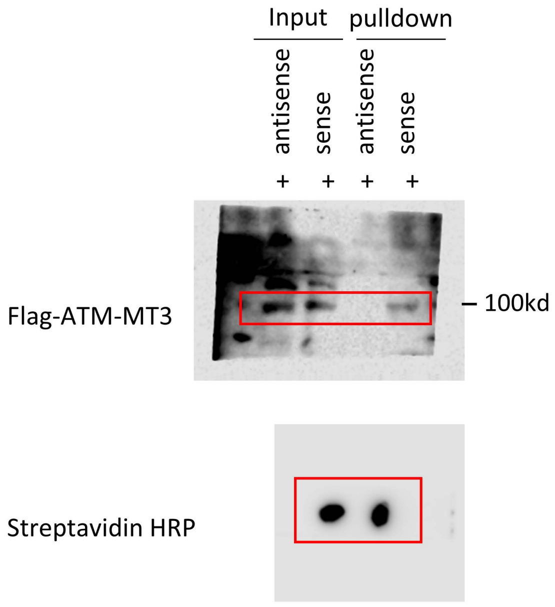

Input
pulldown
antisense
sense
antisense
sense
+
+
+
+
Flag-ATM-MT3
100kd
Streptavidin HRP

Figure 3L

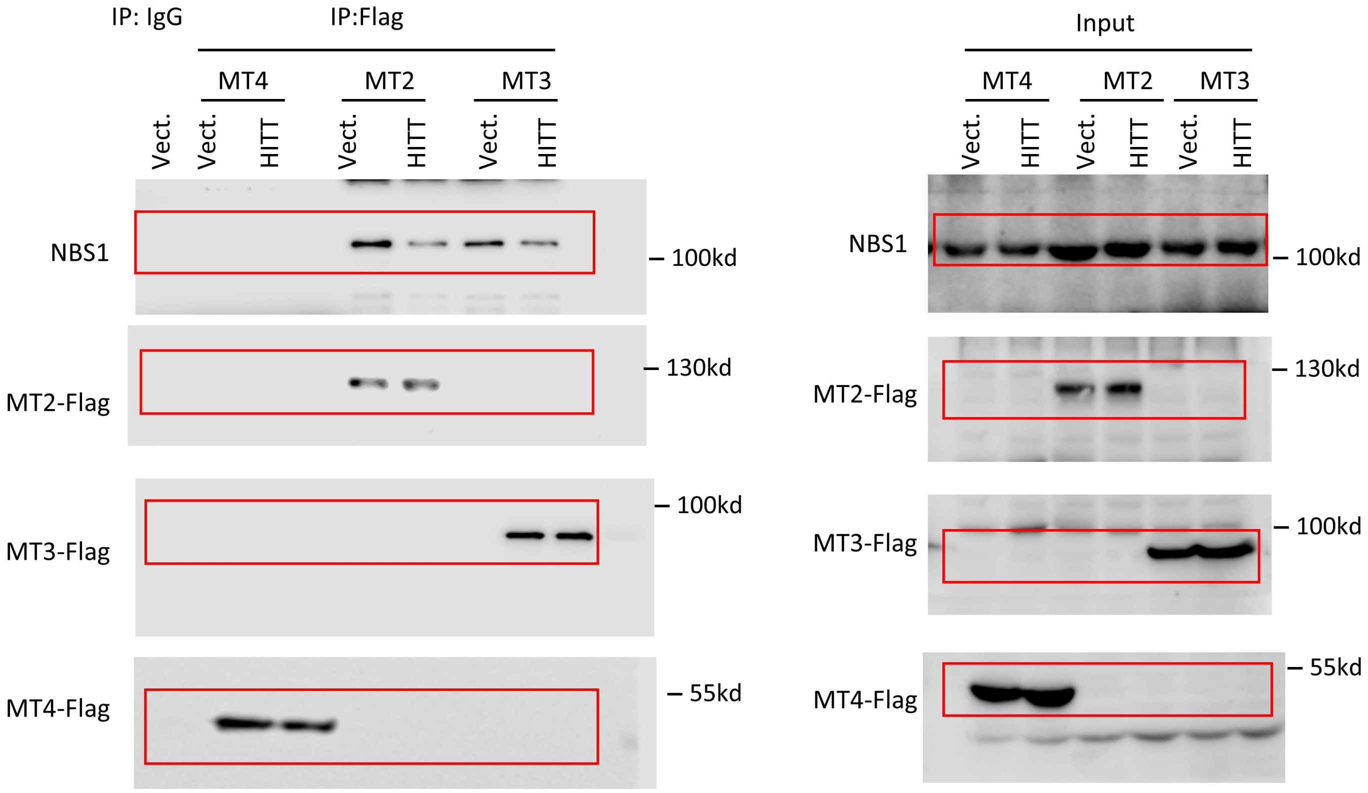
IP: IgG
IP:Flag
MT4
MT2
MT3
Vect.
Vect.
HITT
Vect.
HITT
Vect.
HITT
NBS1
MT2-Flag
MT3-Flag
MT4-Flag
100kd
130kd
100kd
55kd
Input
MT4
MT2
MT3
Vect.
HITT
Vect.
HITT
Vect.
HITT
NBS1
MT2-Flag
MT3-Flag
MT4-Flag
100kd
130kd
100kd
55kd

Figure 4B

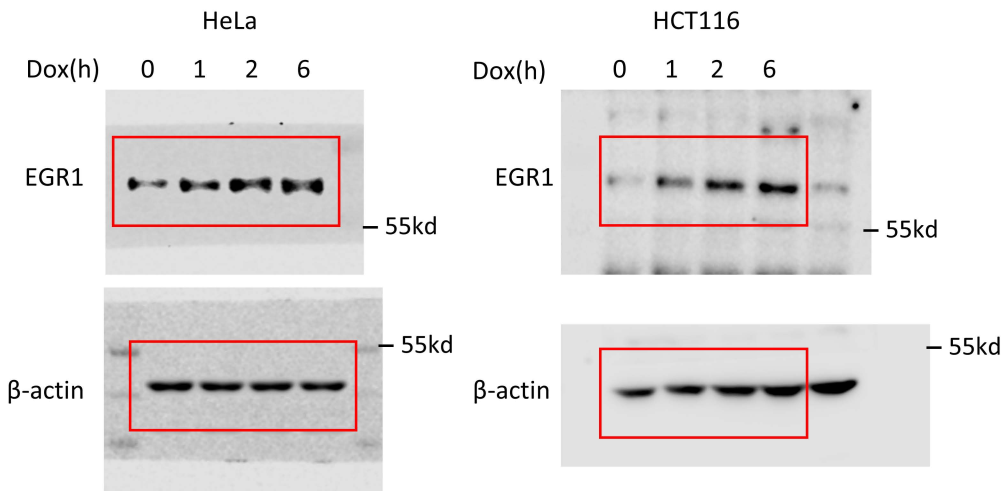

HeLa
Dox(h) 0 1 2 6
EGR1
55kd
55kd
β-actin
HCT116
Dox(h) 0 1 2 6
EGR1
55kd
55kd
β-actin

Figure 4C

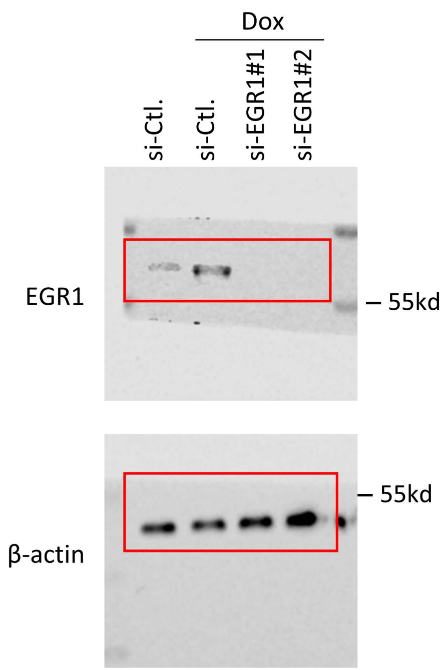

Figure 4D

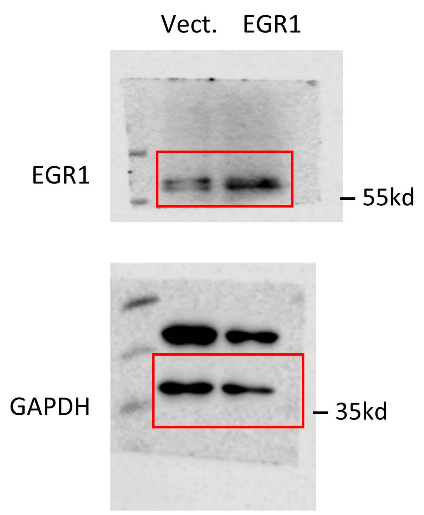

Figure 4F

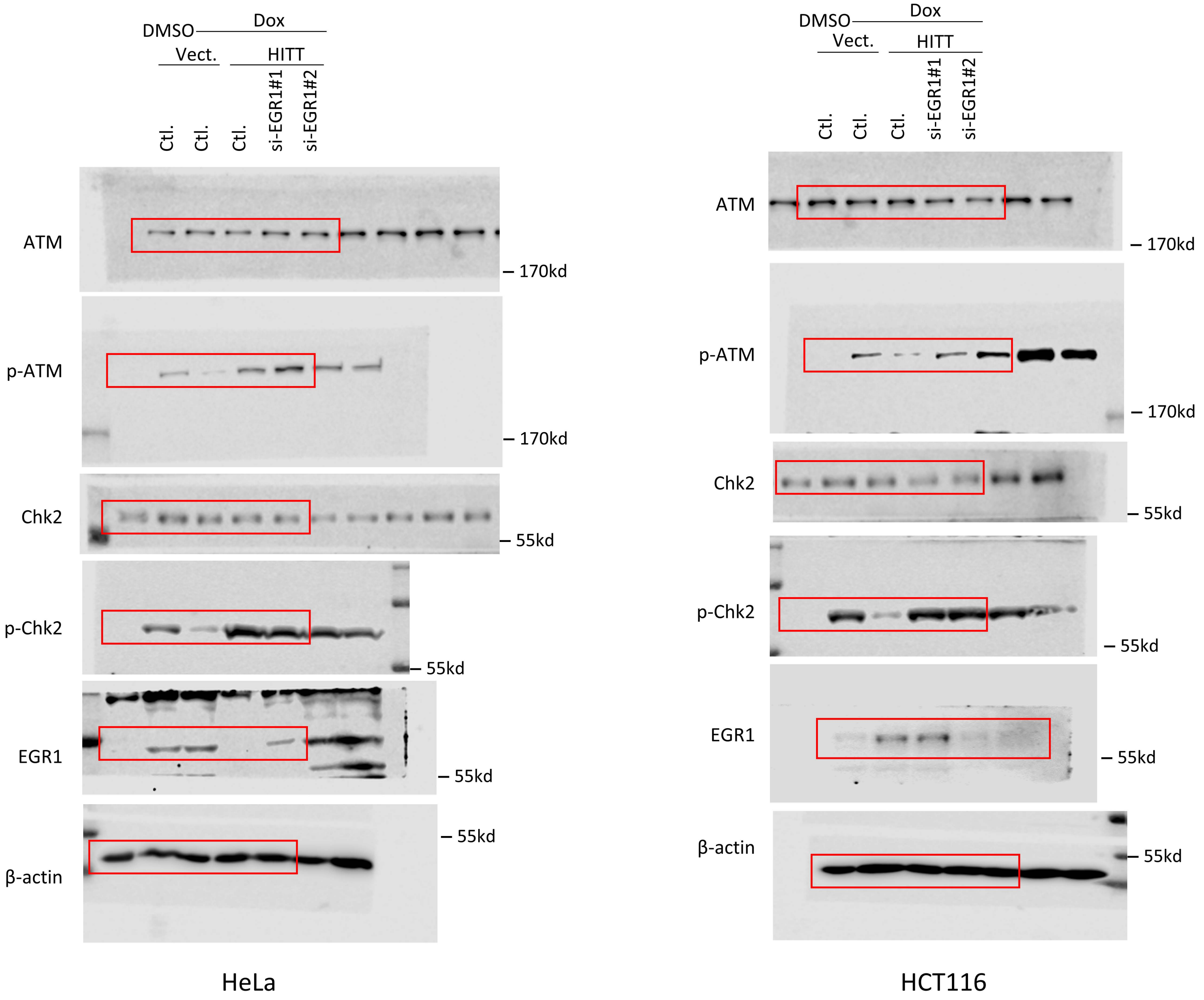

DMSO
Dox
Vect.
HITT
Ctl.
Ctl.
Ctl.
si-EGR1#1
si-EGR1#2
ATM
170kd
p-ATM
170kd
Chk2
55kd
p-Chk2
55kd
EGR1
55kd
β-actin
55kd
HeLa
DMSO
Dox
Vect.
HITT
Ctl.
Ctl.
Ctl.
si-EGR1#1
si-EGR1#2
ATM
170kd
p-ATM
170kd
Chk2
55kd
p-Chk2
55kd
EGR1
55kd
β-actin
55kd
HCT116

Figure 6F

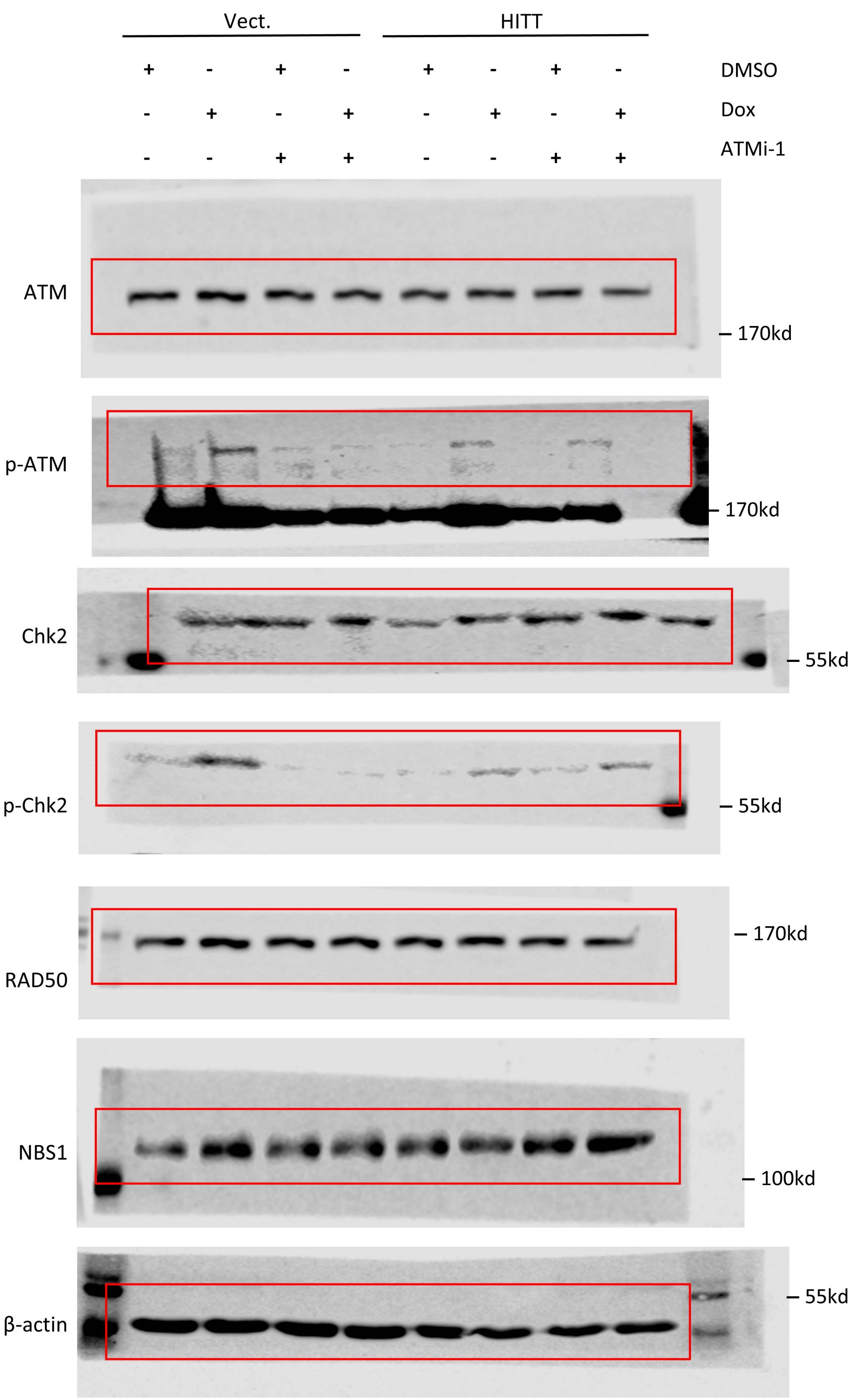
Vect.
HITT
+ - + - + - + -
DMSO
- + - + - + - +
Dox
- - + + - - + +
ATMi-1
ATM
170kd
p-ATM
170kd
Chk2
55kd
p-Chk2
55kd
RAD50
170kd
NBS1
100kd
β-actin
55kd

Figure S1F

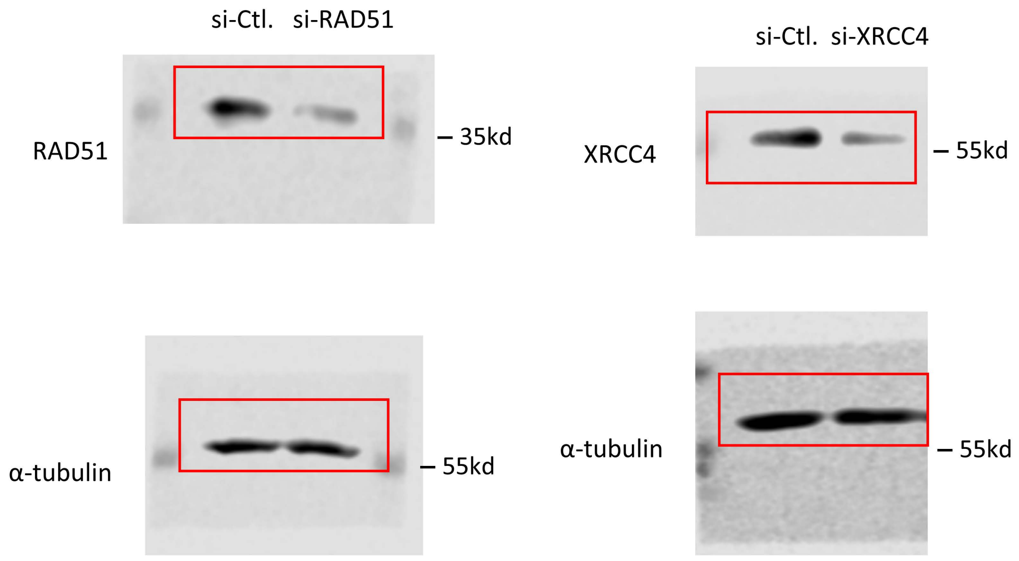
si-Ctl.
si-RAD51
RAD51
35kd
α-tubulin
55kd
si-Ctl.
si-XRCC4
XRCC4
55kd
α-tubulin
55kd

Figure S2C

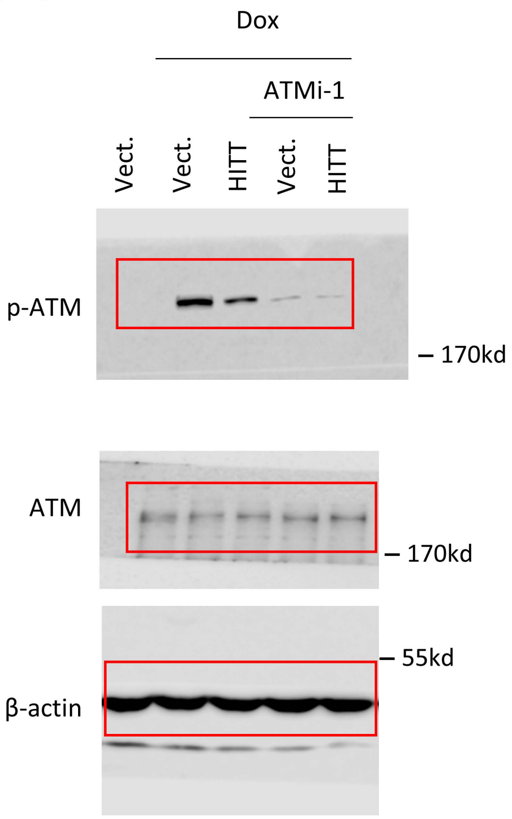
Dox
ATMi-1
Vect.
Vect.
HITT
Vect.
HITT
p-ATM
170kd
ATM
170kd
55kd
β-actin

Figure S2D

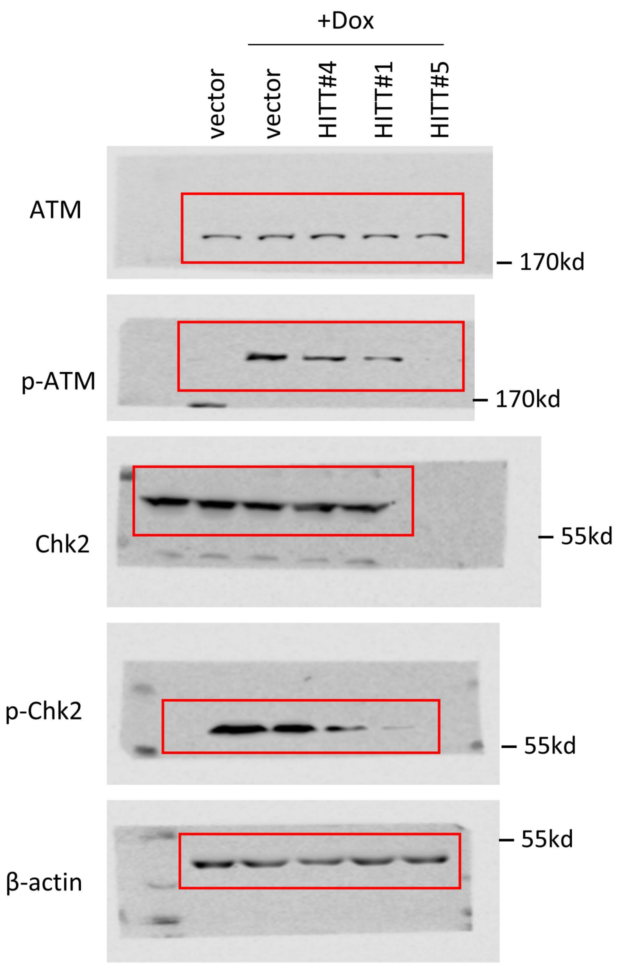

+Dox
vector
vector
HITT#4
HITT#1
HITT#5
ATM
170kd
p-ATM
170kd
Chk2
55kd
p-Chk2
55kd
β-actin
55kd

Figure S2E

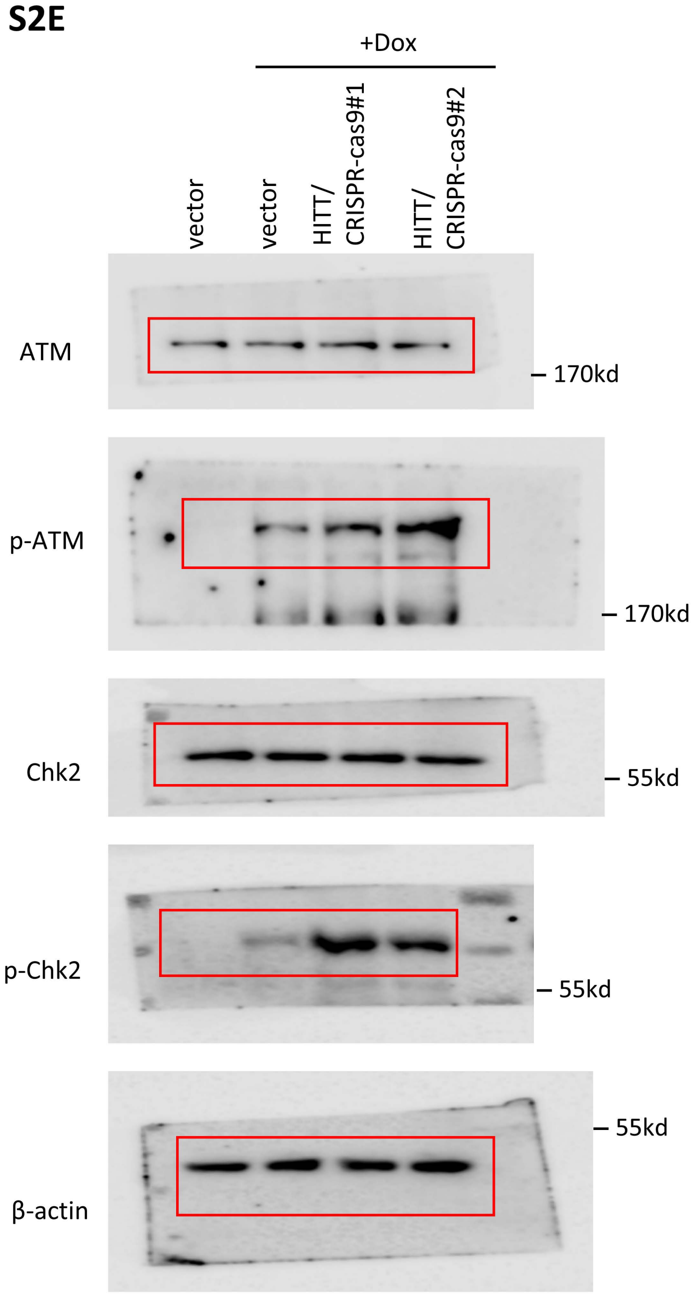
+Dox
vector
vector
HITT/
CRISPR-cas9#1
HITT/
CRISPR-cas9#2
ATM
170kd
p-ATM
170kd
Chk2
55kd
p-Chk2
55kd
β-actin
55kd

Figure S2F

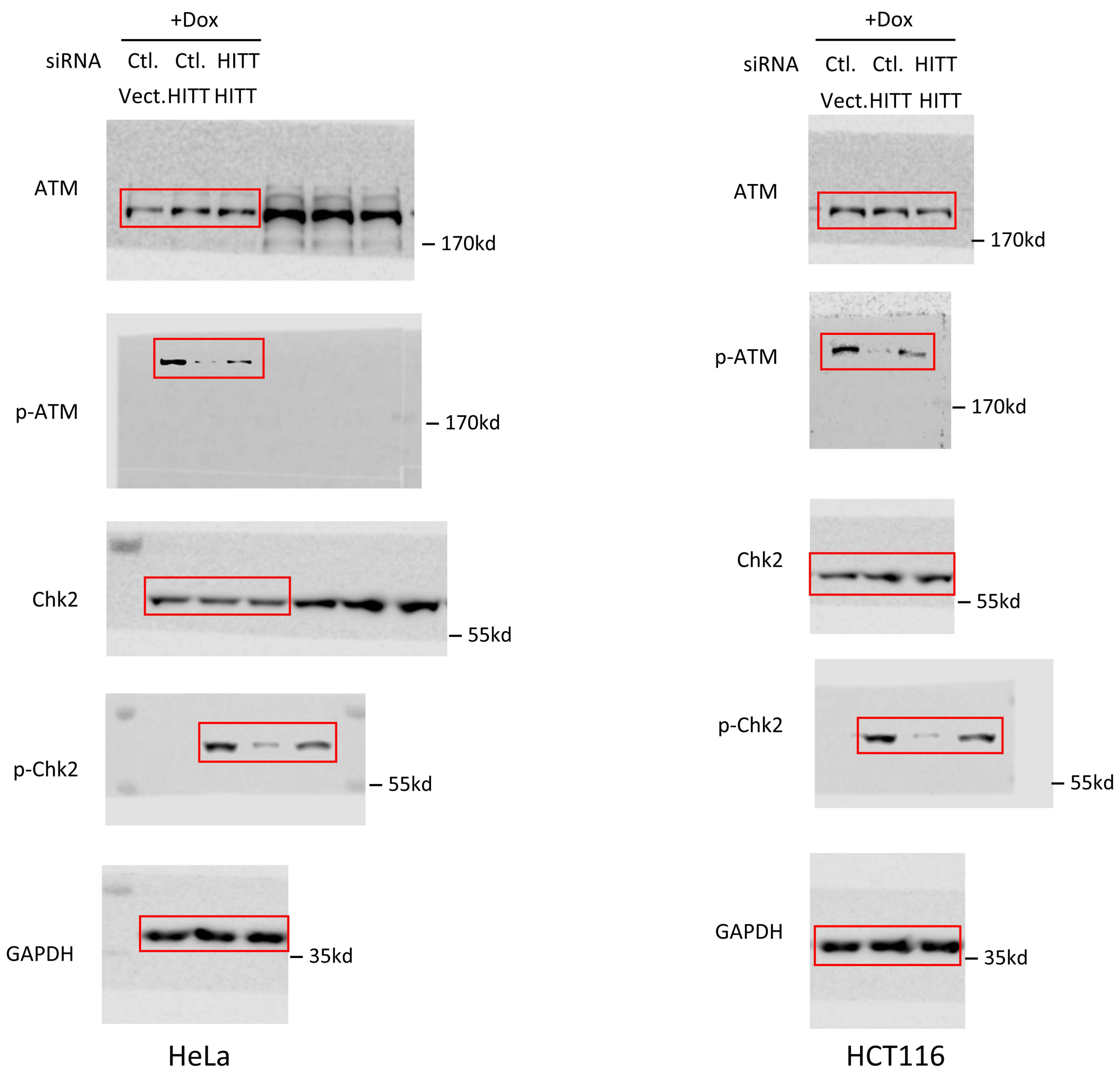
+Dox
siRNA
Ctl.
Ctl.
HITT
Vect.
HITT
HITT
ATM
170kd
p-ATM
170kd
Chk2
55kd
p-Chk2
55kd
GAPDH
35kd
HeLa
+Dox
siRNA
Ctl.
Ctl.
HITT
Vect.
HITT
HITT
ATM
170kd
p-ATM
170kd
Chk2
55kd
p-Chk2
55kd
GAPDH
35kd
HCT116

Figure S3B

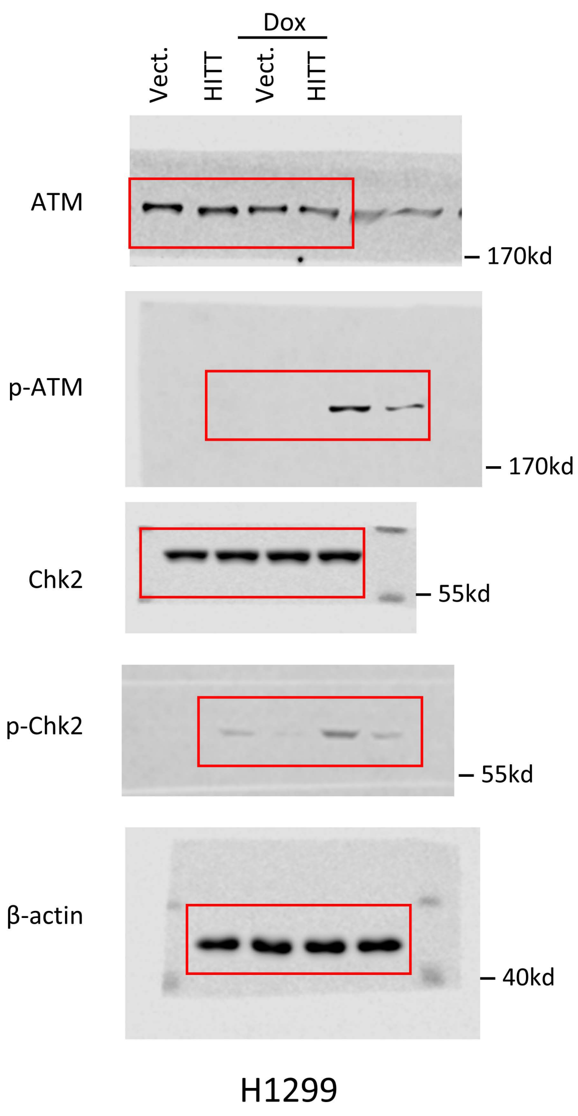

Dox
Vect.
HITT
Vect.
HITT
ATM
170kd
p-ATM
170kd
Chk2
55kd
p-Chk2
55kd
β-actin
40kd
H1299

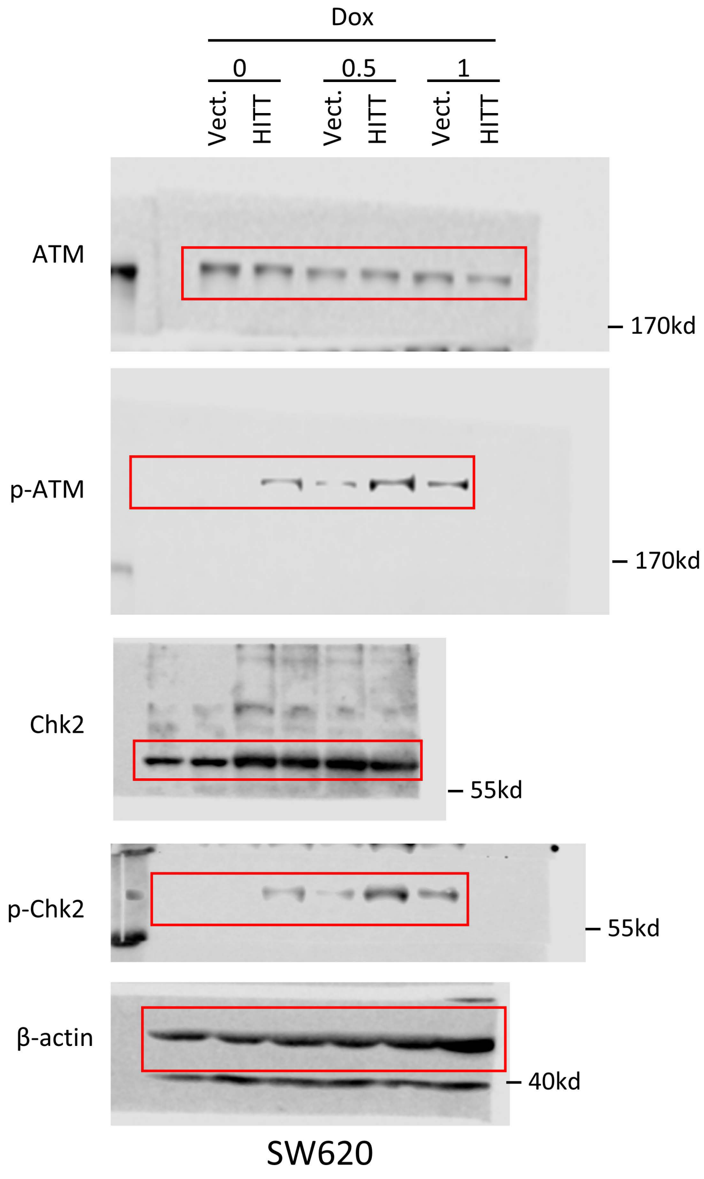

Dox
0
0.5
1
Vect.
HITT
Vect.
HITT
Vect.
HITT
ATM
170kd
p-ATM
170kd
Chk2
55kd
p-Chk2
55kd
β-actin
40kd
SW620

Figure S3C

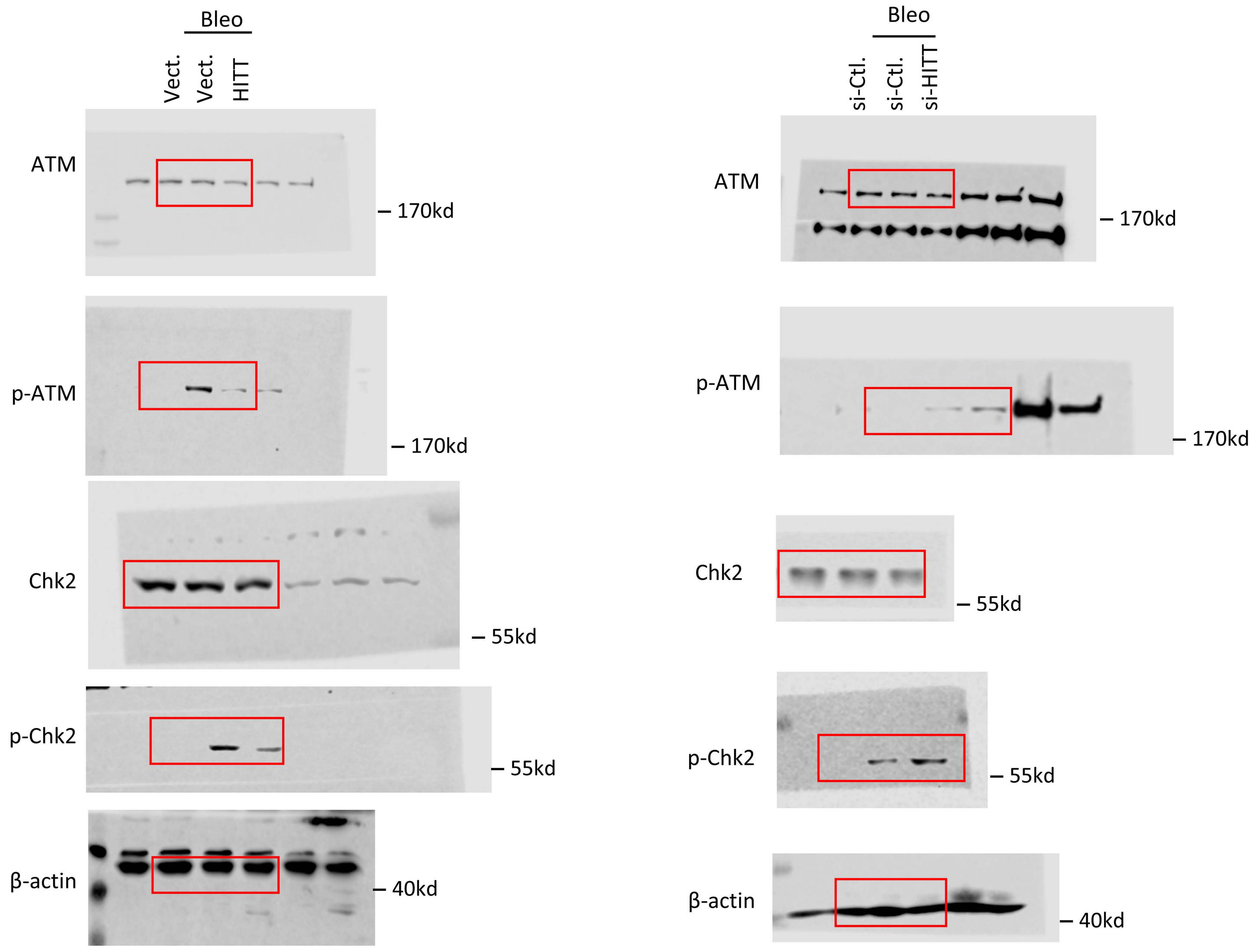
Bleo
Vect.
Vect.
HITT
ATM
170kd
p-ATM
170kd
Chk2
55kd
p-Chk2
55kd
β-actin
40kd
Bleo
si-Ctl.
si-Ctl.
si-HITT
ATM
170kd
p-ATM
170kd
Chk2
55kd
p-Chk2
55kd
β-actin
40kd

Figure S3D

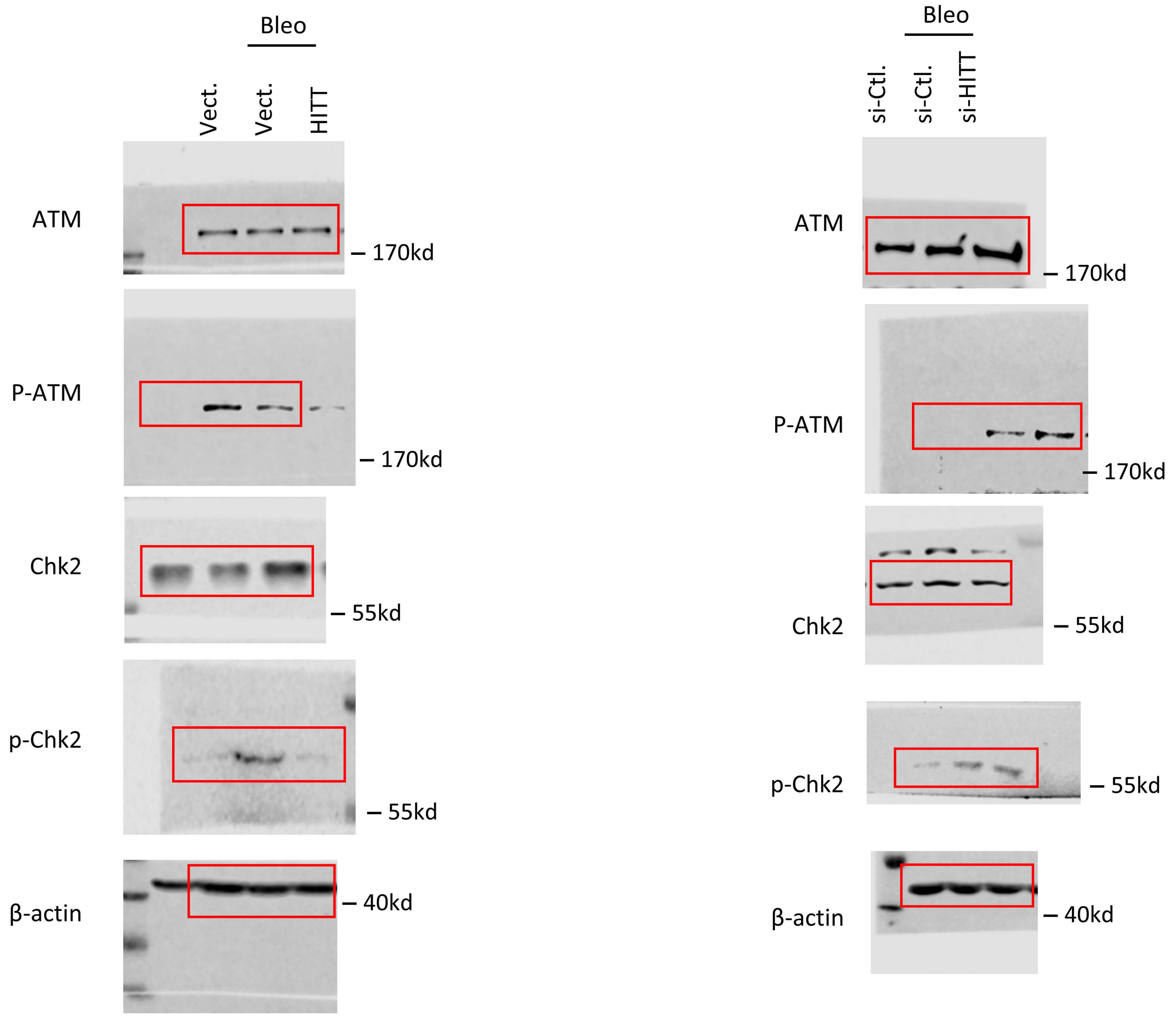
Bleo
Vect.
Vect.
HITT
ATM
170kd
P-ATM
170kd
Chk2
55kd
p-Chk2
55kd
β-actin
40kd
Bleo
si-Ctl.
si-Ctl.
si-HITT
ATM
170kd
P-ATM
170kd
Chk2
55kd
p-Chk2
55kd
β-actin
40kd

Figure S3E

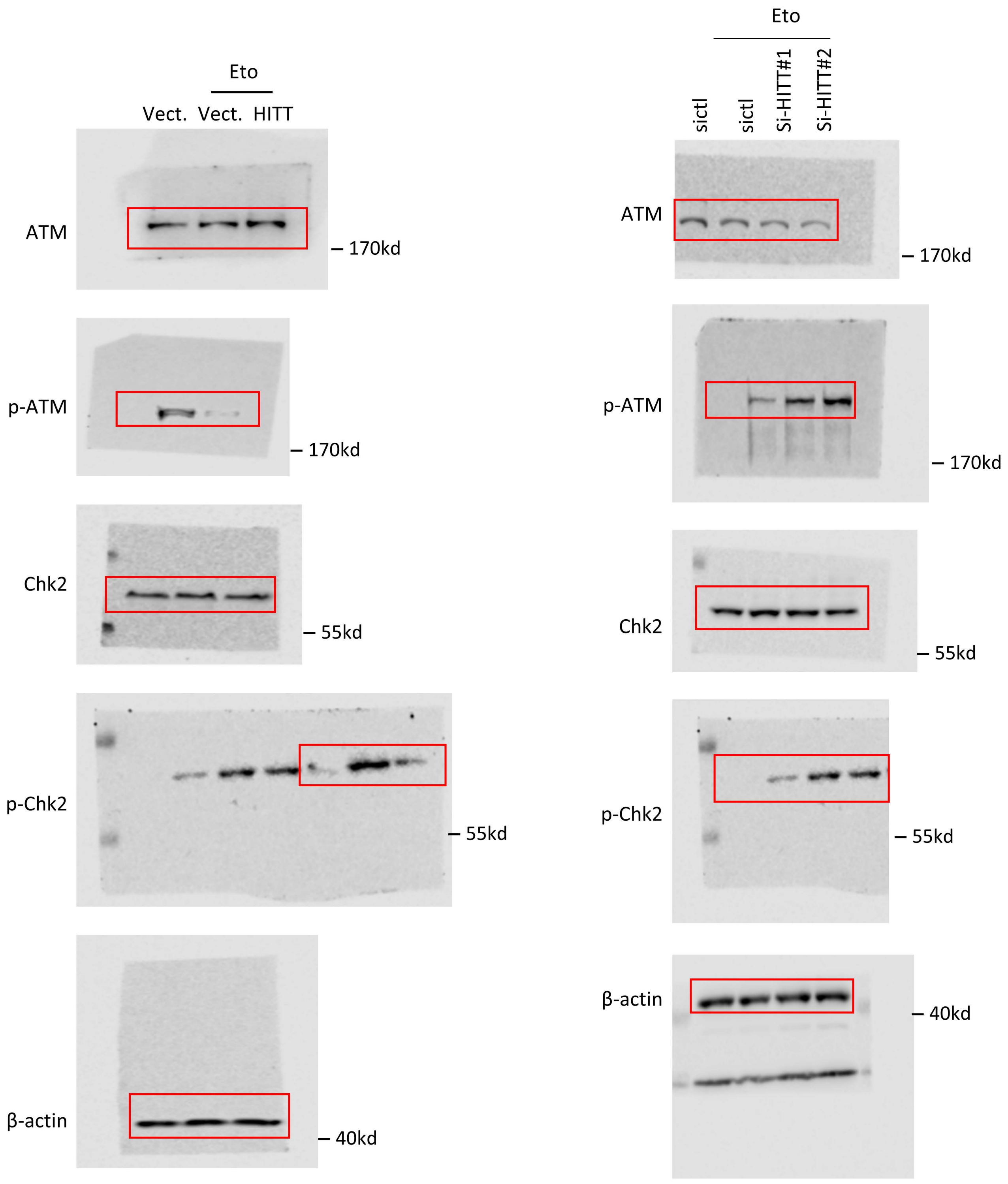
Eto
Vect.
Vect.
HITT
ATM
170kd
p-ATM
170kd
Chk2
55kd
p-Chk2
55kd
β-actin
40kd
Eto
sictl
sictl
Si-HITT#1
Si-HITT#2
ATM
170kd
p-ATM
170kd
Chk2
55kd
p-Chk2
55kd
β-actin
40kd

Figure S4C

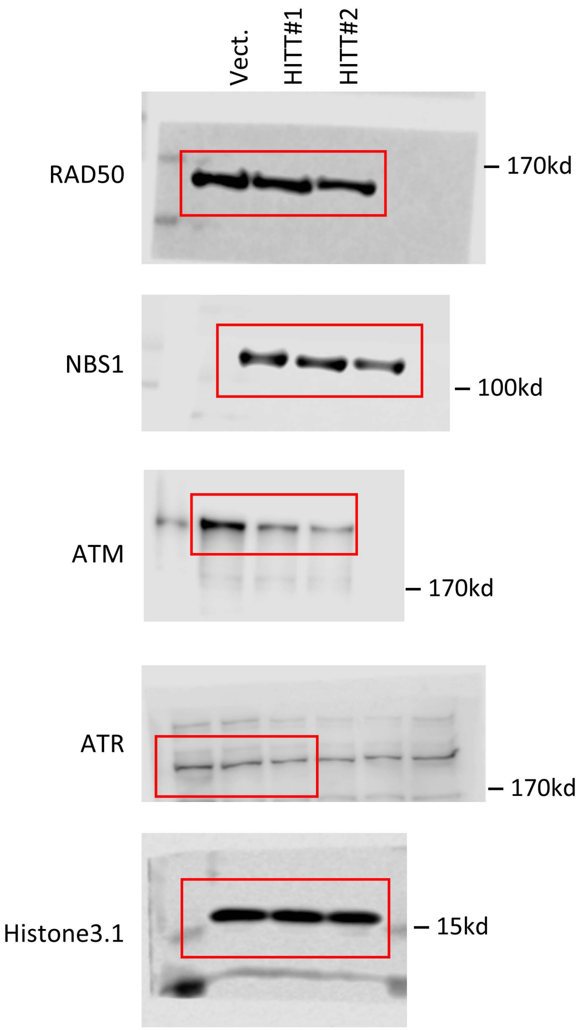

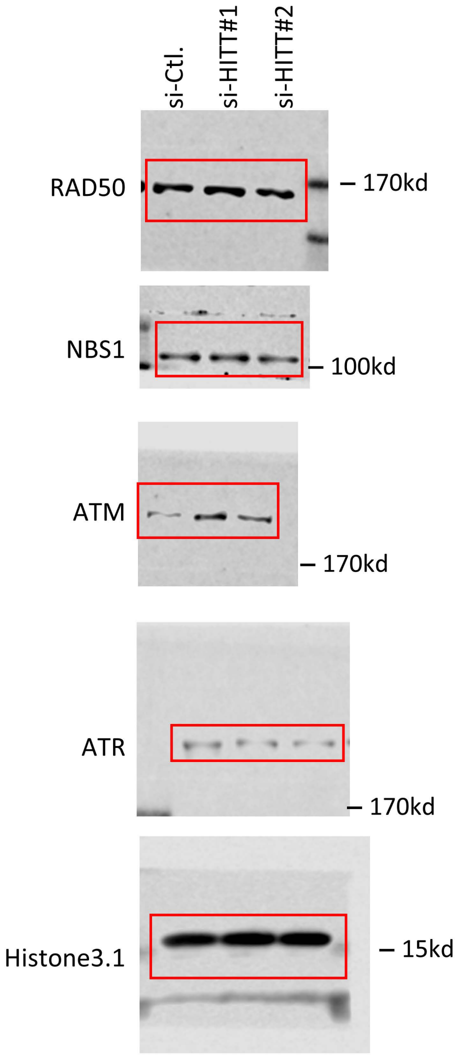

Chromatin-fraction

Figure S5C

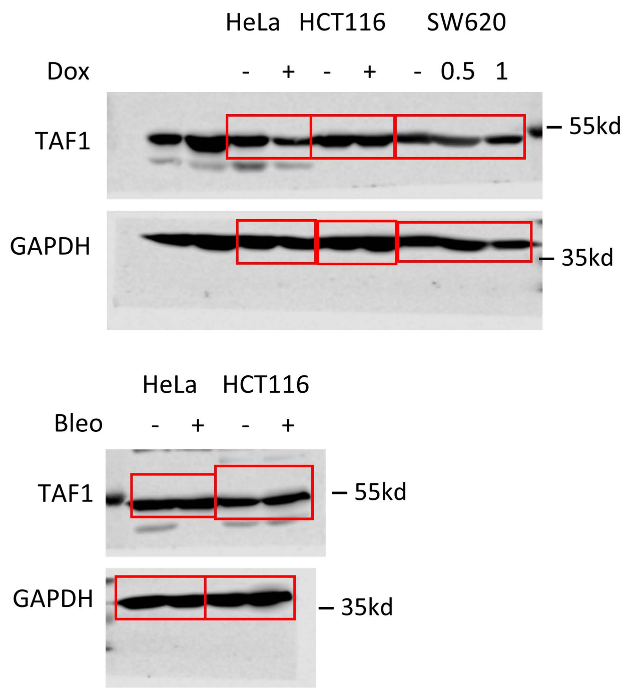
HeLa HCT116 SW620
Dox - + - + - 0.5 1
TAF1
55kd
GAPDH
35kd
HeLa HCT116
Bleo - + - +
TAF1
55kd
GAPDH
35kd

Figure S5D

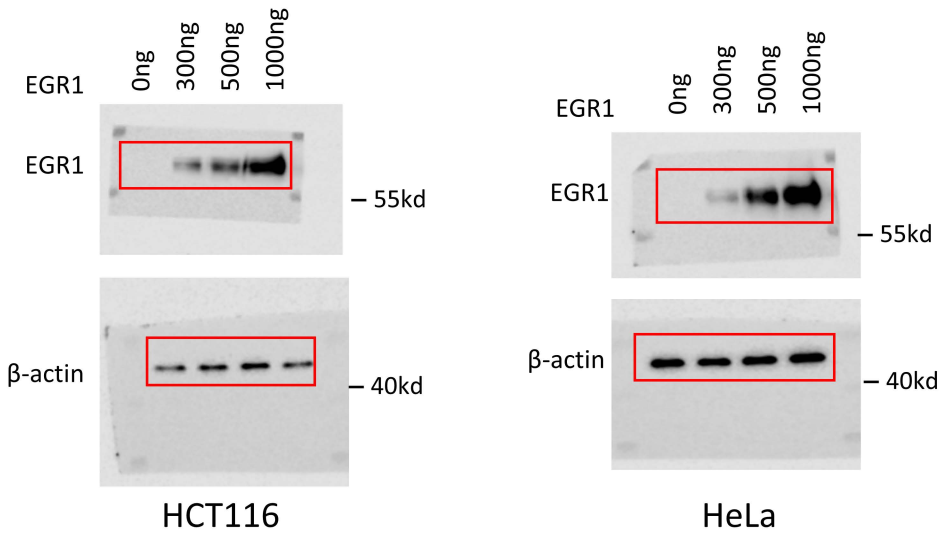
EGR1
0ng
300ng
500ng
1000ng
EGR1
55kd
β-actin
40kd
HCT116
EGR1
0ng
300ng
500ng
1000ng
EGR1
55kd
β-actin
40kd
HeLa

Figure S5E

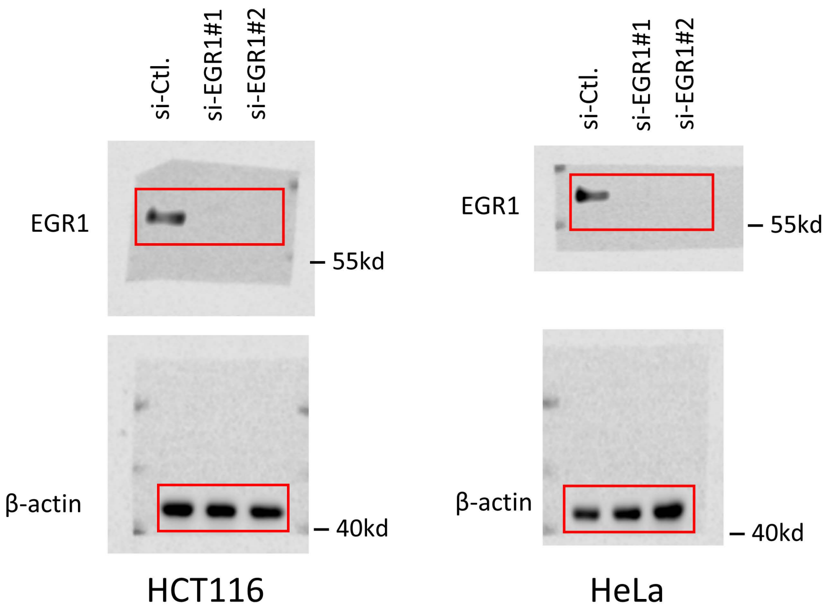
si-Ctl.
si-EGR1#1
si-EGR1#2
EGR1
55kd
β-actin
40kd
HCT116
si-Ctl.
si-EGR1#1
si-EGR1#2
EGR1
55kd
β-actin
40kd
HeLa

Figure S5F

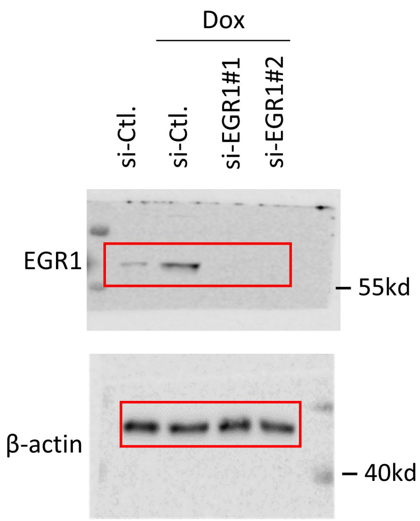

Figure S6B

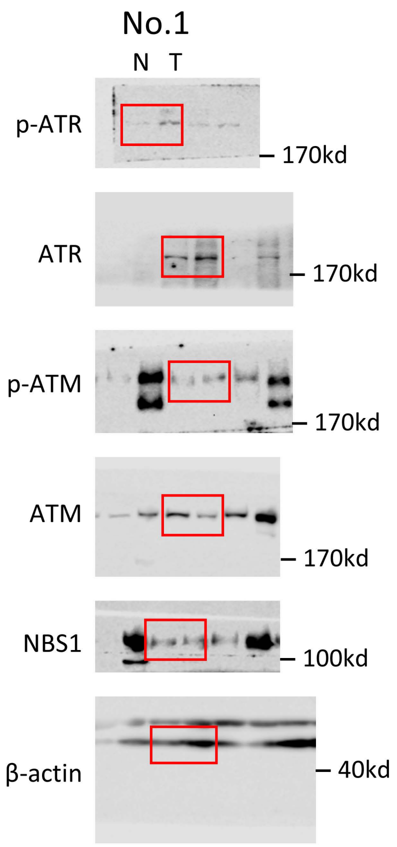
No.1
N T
p-ATR
170kd
ATR
170kd
p-ATM
170kd
ATM
170kd
NBS1
100kd
β-actin
40kd
No.2
N T
p-ATR
170kd
ATR
170kd
p-ATM
170kd
ATM
170kd
NBS1
100kd
β-actin
40kd
No.3
N T
p-ATR
170kd
ATR
170kd
p-ATM
170kd
ATM
170kd
NBS1
100kd
β-actin
40kd
